# Cytotoxic activity of selinexor in BRAF^V600E^-driven pediatric low-grade glioma 3D cultures

**DOI:** 10.1186/s40478-026-02461-1

**Published:** 2026-10-22

**Authors:** Constantia Zeiser, Marta Emperador, Sonja Herter, Lisa Sigaud, Alexandra Stroh-Dege, Anna Loboda, Anna Kolodziejczak, Simon Zeuner, Lea Joy Digel, Karen Frese, Bruno Chilian, Micaela Freitas, Jens M. Kelm, Ingrid Ǿra, Daniel Vogl, Martin Ulrich Schuhmann, Heike Peterziel, Romain Sigaud, Florian Selt, Till Milde, Olaf Witt, Ina Oehme

**Affiliations:** 1 Hopp Children’s Cancer Center Heidelberg (KiTZ), 69120 Heidelberg, Germany; 2 Clinical Cooperation Unit Pediatric Oncology, German Cancer Research Center (DKFZ) and German Cancer Consortium (DKTK), 69120 Heidelberg, Germany; 3 National Center for Tumor Diseases (NCT), NCT Heidelberg, Heidelberg University Hospital, 69120 Heidelberg, Germany; 4 Faculty of Medicine, Heidelberg University, 69120 Heidelberg, Germany; 5 Faculty of Biosciences, Heidelberg University, 69120 Heidelberg, Germany; 6 Department of Pediatric Oncology, Hematology, Immunology and Pulmonology, Heidelberg University Hospital, 69120 Heidelberg, Germany; 7 Department of Medicine III, Heidelberg University, German Centre for Cardiovascular Research (DZHK), partner site Heidelberg/Mannheim, 69120 Heidelberg, Germany; 8 TRI Thinking Research Instruments GmbH, Große Freiheit 77, 22767 Hamburg, Germany; 9 Precomb Therapeutics AG, Hombrechtikon, Switzerland; 10 Lund University, Lund, Sweden; 11 Karolinska University Hospital, Stockholm, Sweden; 12 Department of Neurosurgery, Section of Pediatric Neurosurgery, University Hospital Tuebingen, Hoppe-Seyler-Str. 3, Baden-Württemberg 72076 Tuebingen, Germany; 13 Department of Neurosurgery, Centre of Neurofibromatosis and Schwannomatosis, Centre for Rare Diseases, Division of Pediatric Neurosurgery, University Hospital Tübingen, Tübingen, Germany; 14 Department of Pediatrics and Adolescent Medicine, University Hospital Jena, Friedrich Schiller University Jena and Comprehensive Cancer Center Central Germany (CCCG), Jena, Germany

**Keywords:** Brain tumor, Pediatric low-grade glioma, 3D culture, Ex vivo drug profiling, MAPK pathway, Exportin-1 (XPO1), Synergy screening, Targeted therapy, Functional precision oncology, Drug repurposing

## Abstract

Pediatric low-grade gliomas (pLGGs) are the most common primary brain tumors in children and frequently harbor activating RAS/MAPK pathway alterations, including BRAF^V600E^. Although MEK inhibitors (MEKi) show clinical activity, responses are often incomplete and tumors commonly regrow after treatment discontinuation, highlighting the need for therapies that induce sustained regression. To systematically identify cytotoxic treatments, we initially screened 87 clinically relevant drugs in BRAF^V600E^-mutant BT40 3D multicellular aggregates and subsequently evaluated the prioritized XPO1-targeting strategy in BRAF^V600E^-mutant DKFZ-BT314 spheroids in proliferative (ON) and senescent (OFF) states and in fresh pLGG tissue cultures. High-content imaging with machine-learning-based analysis identified XPO1 inhibition as a vulnerability across models. The FDA-approved selective XPO1 inhibitor selinexor was active alone and in combination with MEKi. MEK-XPO1 co-inhibition reduced viable 3D culture area by more than 30% and suppressed regrowth for at least 10 d, even after medium change. DRAQ7 positivity and caspase-3/7 activation demonstrated cell death in the 3D cultures, while complementary 2D analyses showed PARP cleavage, nuclear fragmentation, and caspase activation. Transcriptomic analyses of pleomorphic xanthoastrocytoma patient samples and BRAF^V600E^-induced senescence models revealed upregulation of MAPK, TGFβ, TP53, and FOXO pathway genes and anti-apoptotic BCL2 family members, including MCL-1. XPO1 inhibition reduced MCL-1 protein levels, consistent with disruption of anti-apoptotic signaling. Ex vivo drug sensitivity profiling of fresh tumor tissue from 20 patients from the LOGGIC Core BioClinical Data Bank demonstrated sensitivity to XPO1 inhibition at clinically achievable concentrations. In an exploratory BT40 zebrafish embryo xenograft experiment, 48 h of combination treatment numerically attenuated tumor growth compared with solvent treatment, although the difference was not statistically significant. Overall, our findings support XPO1 inhibition, alone or combined with MEK inhibition, as a candidate therapeutic strategy for BRAF^V600E^-driven pLGG that warrants further preclinical evaluation.

**Supplementary Information:**

The online version contains supplementary material available at https://doi.org/10.1186/s40478-026-02461-1.

## Introduction

Pediatric low-grade gliomas (pLGGs) represent the most prevalent form of primary central nervous system (CNS) tumors in children aged 0–14 years, accounting for 30–40% of all cases [55, 56]. pLGGs are a heterogeneous group of tumors defined as grade 1 or 2 according to the fifth edition of the World Health Organization (WHO) classification of tumors of the central nervous system (2021) [5]. The current standard of care (SOC) primarily uses maximal safe surgical resection when feasible. This approach has been shown to result in a favorable overall survival rate of 82% after 20 years. However, progression-free survival (PFS) in children requiring postsurgical adjuvant chemotherapy remains comparatively low at 58%, and a significant number of children experience long-term morbidity [1]. Although gross total resection is the most important factor in determining progression-free survival, the preferred adjuvant therapies for relapsed or progressive pLGG include chemotherapy with carboplatin or vinca alkaloids and, in some cases, photon or proton radiotherapy [62]. From a molecular perspective, pLGG can be conceptualized as a single pathway disease, as the majority of cases exhibit an activating alteration in the RAS-mitogen-activated protein kinase (RAS/MAPK) pathway. The most prevalent alterations include *KIAA1549::BRAF*-fusion, *BRAF*^*V600E*^ mutation, *neurofibromin 1 (NF1)* mutations, and *fibroblast growth factor receptor 1 (FGFR1)* alterations [61]. This resulted in the pursuit of precision medicine treatment modalities that target the RAS/MAPK pathway. MEK inhibitors (MEKi; trametinib, binimetinib) have demonstrated robust inhibition of the MAPK pathway [65, 74] and have been investigated in numerous clinical trials [58, 60, 67], (ClinicalTrials.gov ID NCT02285439). Although patients often benefit from MEKi during treatment, resulting in stable disease, tumor rebound may occur once treatment is stopped, which is a significant clinical challenge [6, 24, 67]. In pLGGs, activating BRAF fusions—most commonly *KIAA1549::BRAF* —represent the predominant MAPK-driving alteration, occurring in the majority of cases, whereas the BRAF^V600E^ point mutation is less frequent but clinically more concerning due to its association with higher-risk disease and increased likelihood of progression [44]. BRAF^V600E^–mutant tumors exhibit strong adaptive MAPK reactivation and rapid ERK rebound following inhibitor withdrawal [38, 49]. Recently, Selt et al. [66] developed patient-derived and engineered model systems that specifically capture the biology of BRAF^V600E^–mutant pediatric LGGs. These include primary cultures in which oncogene-induced senescence (OIS) is transiently bypassed through a reversible Simian Vacuolating Virus 40 Large T Antigen (SV40-TAg) system, enabling the controlled expansion of otherwise growth-restricted BRAF^V600E^ tumor cells while preserving their native signaling characteristics. Importantly, upon release of the bypass, the cells re-engage MAPK-driven senescence programs, providing a physiologically relevant platform to study BRAF^V600E^-dependent tumor biology, adaptive signaling, and therapeutic vulnerabilities in vitro.

In 2023, the combination of dabrafenib and trametinib has been approved as firstline adjuvant therapy of BRAF^V600E^ mutant pLGG and to be superior compared with chemotherapy in a randomized trial [11]. Furthermore, Tovorafenib has demonstrated significant activity in relapsed BRAF-altered pLGG resulting in its approval by the FDA [37]. The activation of the MAPK pathway in pLGGs has been demonstrated to induce oncogene-induced senescence (OIS) [69]. Given that senescent cells do not respond to anti-proliferative treatments, this can be proposed as an explanation for the observed treatment resistance [66]. Therefore, there is an ongoing need to identify drug combinations that, when used with MEKi treatment, will fully and specifically eradicate tumor cells. Selt et al. recently demonstrated that BCL-X_L_ is critical for the survival of senescent pilocytic astrocytoma (PA) tumor cells and provided proof-of-principle for the use of BCL-X_L_-dependent senolytics [66].

Selective inhibitors of nuclear export (SINEs) represent a novel class of anticancer agents. In 2019, the U.S. Food and Drug Administration (FDA) approved selinexor, an orally bioavailable SINE compound, for the treatment of refractory multiple myeloma. This was followed by approval for the treatment of relapsed/refractory diffuse large B-cell lymphoma in 2020 [4, 8]. Selinexor suppressed tumor growth in an orthotopic glioblastoma patient-derived xenograft (PDX) model [31] and demonstrated excellent blood-brain barrier penetrance in a phase 2 study for recurrent glioblastoma (KING trial) [45]. A pediatric phase I trial of selinexor in children and adolescents with recurrent CNS and solid tumors established a recommended phase II dose of 35 mg/m^2^ administered once weekly. Although no objective responses were observed, prolonged disease stabilization was reported in a subset of patients, including several with high-grade glioma, supporting further evaluation of selinexor in pediatric brain tumors (NCT02323880) [30]. Based on these findings, a phase I/II trial evaluating selinexor in combination with radiotherapy for newly diagnosed pediatric high-grade glioma was initiated (COG ACNS1821; NCT05099003). A cross-sectional analysis of 40 clinical trials evaluating selinexor across multiple cancer types demonstrated clinical activity, with improvements in progression-free survival and objective response rates reported in several studies. However, treatment was also associated with toxicity, highlighting the need for strategies that improve the therapeutic index of selinexor, such as rational combination approaches [22]. While selinexor has been investigated as monotherapy and in combination with other treatment modalities, its potential to synergize with MAPK pathway inhibition has not yet been systematically explored. To our knowledge, no study has evaluated selective inhibitors of nuclear export (SINEs) in combination with MAPK pathway inhibitors as a therapeutic strategy for pLGG. The advanced clinical development of selinexor therefore makes it an attractive candidate for combination approaches in pLGG.

To identify novel treatment options like SINEs, functional precision oncology is becoming increasingly important in addition to alteration-targeted treatment. A large number of compounds can be screened using metabolic activity assays with more physiological patient-derived 3D cultures [47, 51, 59]. Although more challenging with three-dimensional cultures than metabolic assays, live cell imaging-based features provide effective and complementary readouts for assessing drug efficacy [9, 16, 33]. In this study, we identify a novel cytotoxic drug class that enhances MEKi treatment, XPO1 inhibition (XPO1i). We first performed high-content drug screening in BT40 3D multicellular aggregates using 87 clinically relevant compounds, tested alone and in combination with MEK inhibition. The prioritized XPO1-targeting strategy was subsequently evaluated in patient-derived DKFZ-BT314 spheroids in both proliferative (ON) and senescent (OFF) states. To assess the clinical relevance of the identified vulnerability beyond established culture models, we additionally performed ex vivo drug-sensitivity profiling of fresh tumor tissue cultures from 20 patients with pLGG. Complementary mechanistic experiments and an exploratory zebrafish xenograft study were used to characterize treatment-induced cell death and obtain an initial indication of in vivo activity.

## Materials and methods

### Cell culture and patient-derived fresh tissue culture

The pleomorphic xanthoastrocytoma cell line BT40 [10] (BRAF^V600E^ mutation and CDKN2A^del^; kindly provided by Prof. Houghton, University of Texas Health Science Center at San Antonio, USA) and the in-house generated patient-derived PA cell line DKFZ-BT314 [66] (BRAF^V600E^ mutation; generated in the CCU Pediatric Oncology, Heidelberg, Germany) were cultured under standard conditions as already described [65]. Depending on the inducible expression of SV40 large T antigen, the DKFZ-BT314 model can reflect two states, proliferation (DKFZ-BT314_ON) and oncogene-induced senescence (DKFZ-BT314_OFF). DKFZ-BT317_OFF cells were seeded in ABM basal medium with AGM SingleQuot Kit Supplements & Growth Factors. Routine testing using the Multiplex cell Contamination Test (McCT) [63] and DNA fingerprinting authentication (DSMZ) or Multiplex Cell line authentication test (MCA) [14] proved the absence of mycoplasma, viral contaminants, and cross-contamination.

Fresh tissue for ex vivo DSP was obtained through the LOGGIC Core BioClinical Data Bank (Suppl. Table 1) or as part of the INFORM (INdividualized therapy FOr high-Risk childhood Malignancies) program as described by Peterziel et al. [59]. Tumor pieces were homogenized and enzymatically digested, the released DNA was digested with DNase I, and the cell suspension was passed through a strainer to obtain single cells in solution for viability counting. Tumor 3D cultures were kept in serum free tumor stem medium (Neurobasal A (1:2), DMEM-f12, (1:2) supplemented with HEPES Buffer (1:100), MEM sodium pyruvate (1:100), MEM non-essential amino acids (1:100), L-Glutamine (1:100) Penicillin-Streptomycin solution (1:100), B27 Supplement minus vitamin A (1:50), H-EGF (1:5000), H-FGF (1:5000), H-PDGF-AA (1:2000)) and subcultured by dissociation with TrypLE express (Life Technologies).

### Seeding and drug treatment in vitro

Adherent cells were detached using trypsin (Gibco, Thermo Fisher Scientific Inc., Waltham, MA, USA). Cell count and viability were assessed using a ViCELL XR automated cell counter (Beckman Coulter Life Sciences, Indianapolis, IN, USA), and only cells with viabilities exceeding 90% were used for further experiments. All drug screening experiments were performed in round-bottomed 384-well plates (3830, Corning, Corning, NY, USA or BioS). Cells were seeded 24 h before treatment in 25 µL at 500/1500 cells per well (BT40/ DKFZ-BT314). To facilitate formation of three-dimensional cultures, cells were collected by centrifugation (400 × g, 3 min). Wet dispensing of the treatments (Supplementary Tables 2 and 3) was performed using the mosquito^®^ (SPT Labtech, Melbourn, UK) and the TECAN D300e digital drug dispenser (Tecan, Männedorf, Switzerland). To maintain DKFZ-BT314_ON in their proliferative state, at least every 72 h 1 µg/ml doxycycline (Santa Cruz; sc-337691) together with 0.3% Tween^®^ 20 (MP1TWEEN201, MP Biomedicals, Irvine, CA, USA) was added to the appropriate wells using the TECAN D300e. DKFZ-BT314_OFF were withdrawn from doxycycline 5 d prior experiment initiation to allow the cells to revert to their OIS state. DKFZ-BT317_OFF were seeded without doxycycline in 384-well plate format (3000 cells/well). On 4th day after seeding cells were imaged using the ImageXpress Micro Confocal High-content microscope (Molecular Devices, San José, CA, USA) and treated with selinexor, ulixertinib and mirdametinib (DMSO and BztCl as negative and positive control, respectively).

### Image-based readout

To evaluate the effect of treatment on 3D culture size, the 3D cultures were stained by adding 25 µL of medium with tetramethylrhodamine ethyl ester (TMRE, Supplementary Table 4), a non-cytotoxic fluorescent stain detecting only viable cells. Subsequently, the 3D cultures were imaged by a 20X Plan Apo objective at the ImageXpress Micro Confocal High-content microscope (Molecular Devices, San José, CA, USA) both before treatment and after 6 d of treatment. Wells with impurities (e.g., dust) were manually sorted out. The area of the 3D cultures was calculated from the brightfield images and from the maximal intensity projection (MIP) of the fluorescent images using CellProfiler Version 4.2.5 [70] (Supplementary Table 5). For proliferative BT40 and DKFZ-BT314_ON, the change from baseline was calculated by the following formula: Change_from_Baseline = (spheroid_area_day6/spheroid_area_day0)*100)−100. For the non-proliferative DKFZ-BT314_OFF, which are not increasing in 3D culture size due to senescence, percent inhibition of the change from baseline was calculated using the following formula, with MAX representing the mean of all healthy controls (0.1% DMSO), MIN representing the mean of all dead controls (250 nM staurosporine), and x representing individual data points: Percent_inhibition = 100 * [(x−MAX)/(MIN−MAX)]. Data are displayed as residual metabolic activity % = 100% – percent inhibition %. To classify the response to treatment, the response evaluation criteria in solid tumors (RECIST) [21] were adopted for in vitro 3D culture area. Progressive disease (PD) was defined as a minimum 20% increase in 3D culture area, while partial response (PR) corresponded to at least a 30% reduction in 3D culture area. Stable disease (SD) was defined by a change from baseline in the 3D culture area ranging from a 30% decrease to a 20% increase. 3D culture circularity was determined from fluorescence images using CellProfiler. 3D culture boundaries were segmented based on the fluorescent signal, and compactness was calculated as follows: Compactness=Perimeter^2^/ (4π × Area). Circularity = 1/Compactness, where a value of 1.0 represents a perfect circle and lower values indicate increasing deviation from circularity.

### Metabolic activity assay

Metabolic activity was evaluated by adding 25 µl CellTiter-Glo 2.0 Cell Viability Assay (Promega, Madison, WI, USA) to each well. The plates were shaken for 5 min, followed by a 15-min light-protected incubation at room temperature. The TECAN Spark plate reader (TECAN, Männedorf, Switzerland) was used to quantify bioluminescence, which correlates with ATP levels in the cells. The percent inhibition of the metabolic activity was determined using the formula as described above. To initially evaluate the sensitivity to single treatment with the MEK inhibitors binimetinib (Selleckchem, S7007) and trametinib (Selleckchem, S2673), BT40 multicellular aggregates were treated in a ½ logarithmic distribution over concentration ranges from 1 nM to 10 µM and 0.01 nM to 100 nM, respectively. After 6 d, concentrations resulting in 25%/50%/75% inhibition of metabolic activity (IC_25_/IC_50_/IC_75_) were determined using the metabolic activity assay.

### Drug combination screening

Combination drug screens (imaging and metabolic activity) were performed by treating BT40 multicellular aggregates in technical duplicates with an extended version of our in-house drug library [23, 59], both as a monotherapy and in combination with a fixed concentration of binimetinib and trametinib (IC_25_). The drug library included 87 clinically relevant drugs, most of them either European Medicines Agency (EMA) or U.S. Food and Drug Administration (FDA) approved or in late-stage clinical development, in five concentration steps, covering clinically achievable C_max_ [35] (Supplementary Table 2). Validation experiments were performed with two concentrations of binimetinib (IC_25_ and IC_50_) in BT40 multicellular aggregates and DKFZ-BT314_ON/OFF spheroids in five technical duplicates.

To assess treatment combinatorial impact over an extended period (14–16 d) and following drug withdrawal, we applied a dynamic imaging-based protocol in collaboration with PreComb Therapeutics AG (3DTwin^®^, Hombrechtikon, Switzerland). Briefly, BT40 multicellular aggregates were treated with mono- and combination therapies for 4 d. Medium exchange with fresh medium on top of a minimum rest of 15 µl was performed with the INTEGRA ASSIST PLUS pipetting robot and the INTEGRA VOYAGER electronic multichannel pipette (INTEGRA Biosciences GmbH, Biebertal, Germany) to culture the 3D models off treatment for 3 d. Subsequently, drugs were dosed a second time for additional 4 d, followed by a treatment-free (with minimum rest) follow-up off treatment period of 3/5 d. The 3D cultures were imaged every second or third day with the VAIDR V5 automated brightfield and fluorescence microscope or with the ImageXpress Micro Confocal High-content microscope.

### Trypan blue cell viability assay

Cells were seeded as monolayer in 6-well plates at 2,00,000 cells (BT-40) per well and allowed to attach overnight before being treated in technical duplicates for 6 d. The attached cells were collected with their supernatant, which included the dead cells, centrifuged at 500 × g for 5 min, and resuspended in 1 ml medium. Cell viability was assessed with the ViCELL XR automated cell counter as described above.

### Confocal fluorescence microscopy

For high-content fluorescence microscopy (HCM), 2500 cells per well were seeded as a monolayer in a flat-bottom 384-well plate (3540, Corning, Corning, NY, USA) or 500 (BT40) to 1500 (BT314) cells per well in round bottom plates (3D culture). After 24 h, treatment was carried out using the TECAN D300e drug dispenser. At the endpoint of the experiment (3 or 6 d of treatment), cells were stained with fluorescence dyes and imaged with an ImageXpress Micro Confocal High-content microscope at 10x magnification as indicated in Supplementary Table 4. CellProfiler and CellProfiler Analyst Version 3.0.4 [11] was used for automated image analysis, as described in Supplementary Table 6.

### Caspase 3/7 activity assay

To measure caspase-3/7 activity, the Caspase-3/7 Fluorometric Assay (BioVision, Abcam, Cambridge, UK) was performed following the manufacturer’s instructions. Briefly, 2.5 Mio. cells were seeded as monolayers in 10 cm dishes and allowed to attach overnight before treatment for 72 h. Protein lysates of all adherent and detached cells were taken in cell lysis buffer (#BV-1067-100, Axxora (Enzo Life Sciences GmbH, Lörrach, Germany) and stored overnight at − 80 °C. Protein content was assessed using the colorimetric Pierce BCA Protein Assay-Kit (Thermo Fisher Scientific Inc.) and samples were diluted to equal protein amounts of 10 µg of protein in reaction buffer (#JM-1068-20, MBL International, Woburn, MA, USA). 2x reaction buffer containing 10 mM dithiothreitol (DTT) and caspase-3/7 substrate DEVD-AFC were added. Fluorescent readings were taken in technical duplicates in black 96-well plates (655076, Greiner Bio-One GmbH, Frickenhausen, Germany) at a FLUOstar OPTIMA plate reader (BMG LABTECH GmbH, Ortenberg, Germany). Fluorescence was recorded every 10 min for 3 h at 37 °C and excitation/emission filters at 380/520 nm. Caspase-3/7 activity was quantified by subtracting the fluorescence of the blank control from that of the other probes. The logarithm (base 10) of the fold change to the solvent control (0.1% DMSO) was then plotted for analysis.

### Western blotting

Cells were seeded as monolayers in 10 cm-dishes at 1 million cells per dish and allowed to attach overnight before treatment. After 72 h, cells were collected, including the supernatant, and centrifuged at 500 × g for 10 min. Cells were lysed in sodium dodecyl sulfate (SDS) lysis buffer (Tris 0.5 M, hydrochloric acid (HCl) (pH6.8), SDS 2%, 87%-glycerol 10%, dithiothreitol (DTT) 1 mM) for 15 min on ice, followed by protein denaturation at 98 °C for 10 min. Debris was pelleted by centrifugation at 20 000 × g for 15 min at 4 °C, and the supernatant was collected. Samples of equal protein amounts then underwent electrophoretic separation by SDS-PAGE and protein transfer to polyvinylidene fluoride (PVDF) membranes using a semidry electroblot chamber (Trans-Blot^®^ Turbo™ Transfer System, Bio-Rad Feldkirchen, Germany). Transfer of the proteins was assessed by ponceau-red staining. After application of blocking solution for 1 h (Tris-buffered saline, nonfat dry milk 20%, fetal bovine serum (FBS) 20%, bovine serum albumin 3%, normal goat serum 1%, Tween 20 0.2%), membranes were incubated with primary antibodies overnight at 4 °C followed by peroxidase-conjugated secondary antibody. The following primary antibodies were used: anti-GAPDH (JC1682928, Millipore, Burlington, MA, USA), anti-BID (2002, Cell Signaling), anti-PARP (poly(ADD)ribose polymerase) (9542, Cell Signaling) and anti-MCL-1 (D35A5, 5453T Cell Signaling).

### Zebrafish embryo xenograft model

 In vivo zebrafish embryo xenograft was performed as previously described [52, 80] under institutional approval of the Regierungspräsidium Karlsruhe (35-9185.64), which conform to the Guide for the Care and Use of Laboratory Animals published by The US National Institute of Health (NIH Publication No. 85−23, revised 1996). AB strain wild-type zebrafish (Danio rerio) embryos were used. Before xenotransplantation, drug toxicity was assessed by determining the maximum tolerated dose (MTD) as previously published [29]. It is estimated that the extent of compound absorption by zebrafish larvae is 1/10 to 1/20 of the cell culture treatment concentration [25]. We applied the RECIST (Response Evaluation Criteria in Solid Tumors) criteria adapted to zebrafish for the definition of partial response (PR, tumor size ≥ 30% below basal value) and progressive disease (PD, tumor size ≥ 20% above basal value) [80].

### Graphics and statistics

Absolute/relative inhibiting concentrations (IC_25_, IC_50_ and IC_75_ values) were calculated using GraphPad Prism 5 software (Version 5.01, GraphPad Software, San Diego, CA, USA). To assess the effects of the drug combination screen, dose-response curves and multiparametric modified drug sensitivity scores (DSS_asym_) were calculated by the iTReX application (interactive Therapy Response eXploration, https://itrex.kitz-heidelberg.de) [23] through asymmetric curve fitting. To assess potential benefits of drug combinations, the differential combination DSS (dcDSS_asym_) was applied, calculated as previously described [64].

Western blot bands were quantified using ImageJ (Version 1.53c, National Institutes of Health, Bethesda, MD, USA). Caspase activity assays were analyzed with GraphPad Prism (Version 10.1.2., GraphPad Software, San Diego, CA, USA).

Custom Python code for DSP-LOGGIC processing and visualization in heatmaps was generated with assistance from ChatGPT (version GPT-5.2, OpenAI) and subsequently reviewed and adopted by the authors. All other statistics and graphical representations were done using RStudio (version 2023.06.1 + 524, RStudio PBC, Boston, MA, USA) with R (version 4.3.1, R Foundation for Statistical Computing, Vienna, Austria) and R packages ComplexHeatmap (version 2.16.0), dplyr (version 1.1.1), drc (version 3.0.1), ggplot2 (version 3.4.2) and tidyr (version 1.3.0). Normal distribution of analyzed data was confirmed with quantile-quantile plots (Q-Q plots), followed by ANOVA with Tukey’s honestly significant difference (HSD) post-test, calculated using the core ‘stats’ package. **** *p* < 0.0001, *** *p* < 0.001, ** *p* < 0.01, * *p* < 0.05.

Bliss synergy scores for the indicated drug combinations were calculated using the online platform SynergyFinderPlus (https://synergyfinder.org/) [81] based on percent inhibition, as described above. Briefly, MAX corresponded to complete inhibition (1000 nM staurosporine), MIN to no inhibition (0.1% DMSO), and x represented the number of dead (DRAQ7-positive) cells per well relative to the total cell count. The Highest Single Agent (HSA) model is designed to assess the efficacy of a drug combination in comparison to the response of the most effective single agent [20]. To calculate synergy using the Highest Single Agent (HSA) model, inhibition of 3D culture growth was determined using the following formula: inhibition = (x−MAX)/(MIN−MAX), where *MAX* and *MIN* correspond to the mean 3D culture areas of healthy (0.1% DMSO) and dead (250nM staurosporine) controls, respectively, for each cell line and time point, and *x* represents individual data points. HSA scores were defined as the difference between the inhibition by the drug combination and the maximal inhibition achieved by either single agent alone: HSA = inhib_combi – max(inhib_drugA, inhib_drugB). Combinations are considered synergistic when the combined effect exceeded that of the more effective single agent.

### Analysis of gene expression data from R2

Data were retrieved from R2: Genomics Analysis and Visualization Platform (http://r2.amc.nl). The specific datasets used were ‘Exp PLGG (BT314) - Selt − 6 - MAS5.0 - u133p2‘ and ‘Cell line Childhood Cancer (public) - Versteeg − 86 - MAS5.0 - u133p2’ and compared using the R2 MegaSampler tool.

Furthermore ‘Tumor Pediatric Inform - Pilot/Registry − 2900 - tpm - gencode19’ for the patient samples. For the analysis, datasets were reduced to genes of interest as depicted (Suppl. Data Table). Group means were calculated and tested for differences in R2 using ANOVA followed by Tukey’s honestly significant difference post-test.

## Results

### High-content imaging screen identifies favorable MEK inhibitor combination partners in BT40 BRAF^V600E^ cells

**Fig. 1:**
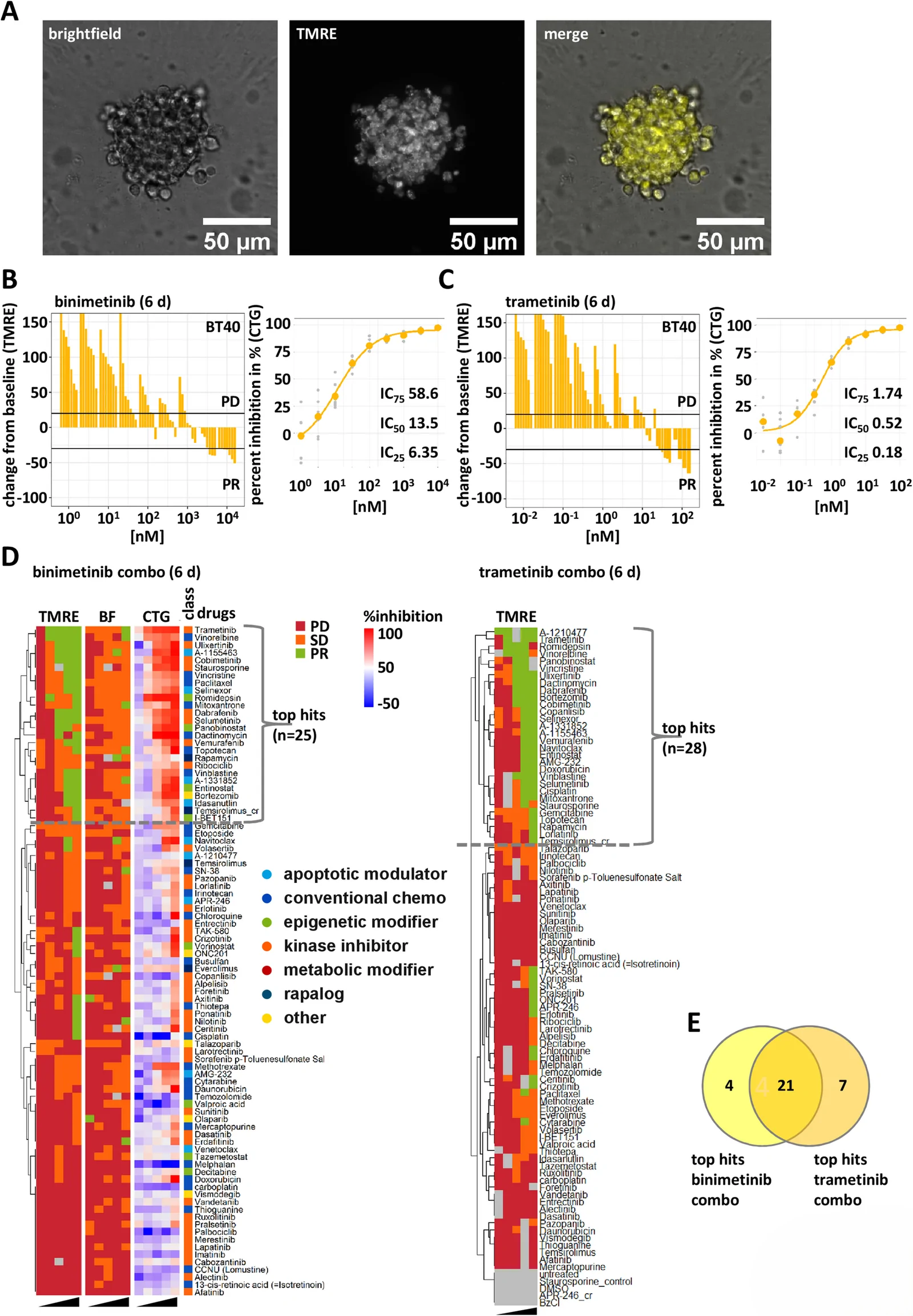
High-content imaging drug screen identifies MEKi combination partners.** A** Representative high-content microscope images of a three-dimensional BT40 multicellular aggregate cultured for 6 d with DMSO and TMRE. From left to right: brightfield image, maximum-intensity projection of TMRE fluorescence, and merged image. Complete TMRE staining (yellow) indicates full cell viability within the whole aggregate after 6 d. **B-C** BT40 multicellular aggregates treated for 6 d with increasing concentrations of binimetinib (**B**) or trametinib (**C**). Bars show change from baseline of TMRE-positive aggregate area; each bar represents an independent technical replicate (*n* = 7). Horizontal lines indicate response thresholds according to RECIST-like criteria, modified for aggregate growth. Dose-response curves show metabolic activity measured using CellTiter-Glo^®^ 2.0. Data are shown as percent inhibition relative to DMSO control. Each grey dot represents a technical replicate (*n* = 7); yellow dots the mean value. Calculated concentrations resulting in 25%, 50%, and 75% inhibition of metabolic activity (IC_25_, IC_50_, IC_75_) are indicated on the graph. **D** Drug sensitivity profiling of BT40 multicellular aggregates with 87 drugs combined with 6.35 nM binimetinib (left) or 0.18 nM trametinib (right) for 6 d (IC_25_). Drugs were tested at five concentrations and classified using the indicated RECIST-like thresholds applied descriptively to change in 3D culture area. A reduction of at least 30% in comparison to the baseline was defined as a partial response (PR; green color in heatmap). An increase in size of at least 20% was classified as progression (PD; red color), while the range between a 20% increase and a 30% decrease was designated as stable disease (SD; orange). Corresponding brightfield-based area measurements and percent inhibition of metabolic activity of the binimetinib screen are shown for comparison. **E** Overlap of top hits from the two independent screens. Abbreviations: MEKi, MEK inhibitor; TMRE, tetramethylrhodamine ethyl ester; IC, inhibitory concentration; PD, progressive disease; PR, partial response; SD, stable disease; RECIST, Response Evaluation Criteria in Solid Tumors

The objective of this study was to identify drug combinations that reduce viable tumor-cell area in 3D culture and induce cytotoxic rather than exclusively cytostatic responses in pLGG. The experimental workflow was designed as a staged discovery-to-validation strategy. BRAF^V600E^-mutant BT40 cells served as the initial high-content screening model for identifying candidate cytotoxic treatments. Prioritized findings were subsequently evaluated in the patient-derived DKFZ-BT314_ON and DKFZ-BT314_OFF spheroid models to determine whether treatment activity extended to both proliferative and senescent states. Finally, ex vivo drug-sensitivity profiling of fresh pLGG tissue cultures from 20 patients was used to assess whether the identified XPO1 vulnerability was also detectable in patient-proximal material. We examined 3D-grown BRAF^V600E^-mutant BT40 cells stained with TMRE and quantified the change in viable 3D culture area 6 d after treatment relative to baseline (Fig. 1A). Morphological analysis demonstrated model-dependent 3D architectures (Suppl. Figure 1). DKFZ-BT314_ON and DKFZ-BT314_OFF formed compact or moderately compact spheroids, whereas BT40 formed stable but irregular multicellular aggregates with comparatively low and variable circularity. We therefore refer to BT40 3D cultures as multicellular aggregates throughout and do not interpret this model as reproducing the architecture or diffusion barriers of compact spheroids. The aggregates remained structurally stable during the assay period, permitting longitudinal image-based measurement of viable area (Suppl. Data). Binimetinib and trametinib affected metabolic activity at low nanomolar concentrations, whereas higher concentrations were required to reduce viable aggregate area by more than 30% (Fig. 1B, C). At clinically achievable concentrations, both MEKi predominantly stabilized BT40 aggregate area, motivating a combination screen designed to identify agents that induce regression. We combined each MEKi with 87 clinically relevant drugs and quantified TMRE-positive area after 6 d (Fig. 1D; Suppl. Figure 2). The binimetinib and trametinib screens identified 25 and 28 top hits, respectively, with 21 overlapping compounds (Fig. 1E). Brightfield area and CellTiter-Glo measurements were consistent with the TMRE-based ranking. Seven distinct drug classes were demonstrated among the drug hits, e.g., histone deacetylase (HDAC) inhibitors, topoisomerase inhibitors, and vinca alkaloids, as well as combinations with well-known activities, such as those involving BCL-2 family members, BRAF, and mechanistic target of rapamycin (mTOR) inhibitors [11, 48]. These results validate our screening approach’s ability to identify promising drug combinations (Table 1). Of the 21 drug hits, 18 were assigned to the specified seven drug classes. Despite already well-characterized drugs, we found a so far undescribed combination partner, the XPO1 inhibitor (XPO1i) selinexor. Thus, the BT40 multicellular-aggregate screen identified MEK-XPO1 co-inhibition as a candidate cytotoxic combination at clinically achievable concentrations.

**Table 1:** In-class effects of combination drug hits

Drug target class	Drug name
BCL-2 family	A-1,155,463; A-1,331,852
BRAF	dabrafenib; vemurafenib
Histone deacetylase (HDAC)	entinostat; panobinostat; romidepsin
MEK/ERK	cobimetinib; selumetinib; trametinib; ulixertinib
Mechanistic target of rapamycin (mTOR)	rapamycin; temsirolimus
Topoisomerase	mitoxantrone; topotecan
Vinca alkaloids	vinblastine; vinorelbine; vincristine

### MEK and XPO1 co-inhibition reduces viable 3D culture area in proliferating and senescent pLGG models

**Fig. 2:**
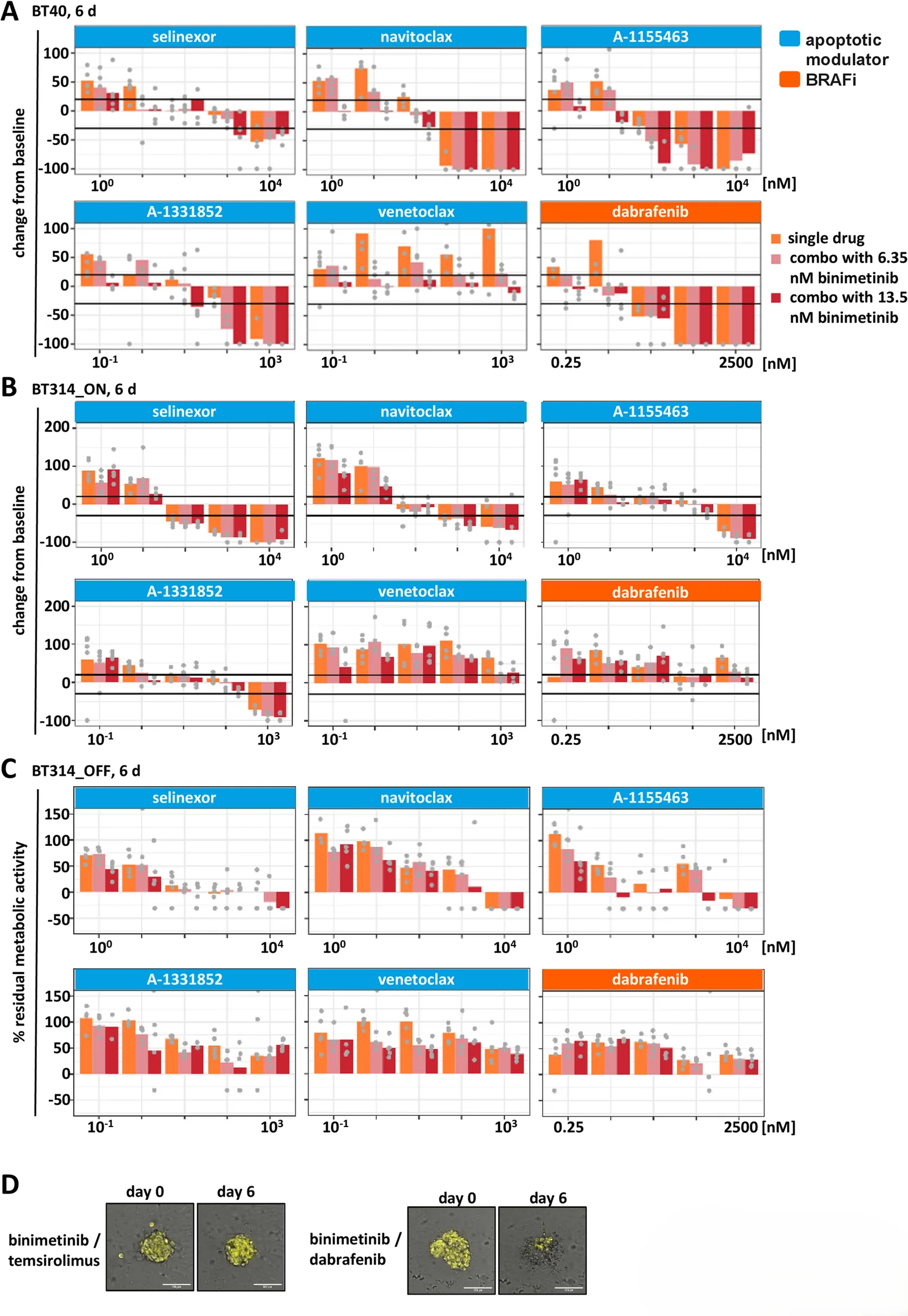
High-content validation screen in proliferating and senescent pLGG 3D culture models.** A-B** Image-based analysis of drug responses in proliferating BT40 multicellular aggregates (**A**) and DKFZ-BT314_ON spheroids (**B**) after 6 d of treatment with single agents or in combination with binimetinib. Values represent change from baseline in TMRE-positive 3D culture area derived from the maximal intensity projection images. Grey dots show technical replicates (*n* = 5) and bars show means. Horizontal lines denote response thresholds based on RECIST-like criteria. All concentrations are given in nM. **C** Residual metabolic activity in senescent DKFZ-BT314_OFF spheroids after 6 d of treatment. Each bar represents a technical replicate (*n* = 5). All concentrations are given in nM. **D** Representative high-content microscopy images of BT40 multicellular aggregates illustrating cytostatic and cytotoxic treatment responses. Shown are merged brightfield images with maximal intensity projections of TMRE fluorescence (yellow). Abbreviations: MEKi, MEK inhibitor; BCL-X_L_i, BCL-X_L_ inhibitor; XPO1i, exportin inhibitor; pLGG, pediatric low-grade glioma; IC, inhibitory concentration; TMRE, tetramethylrhodamine ethyl ester

To validate these findings in an independent BRAF^V600E^-mutant cell line, we tested the XPO1i alone and in combination with MEKi and compared its efficacy with drugs known to show good combination effects (BCL-2 family, BRAFi, mTORi), as well as with the top-hit chemotherapeutics mitoxantrone and vinblastine, using the DKFZ-BT314-ON/OFF BRAF^V600E^ mutant patient derived PA model in comparison to the BT40 BRAF^V600E^ PXA model. The DKFZ-BT314_OFF cells represent a state of cellular senescence, while the doxycycline-induced SV40 DKFZ-BT314_ON model represents a state of proliferative activity [66]. The drugs were tested in combination with binimetinib for their capacity to induce 3D culture size regression through the image-based TMRE change from baseline analyses for BT314-ON and BT40 cells (Fig. 2A, B; Suppl. Figure 3). Given that senescent cells do not proliferate and hence, spheroids do not increase in size, calculations were performed to determine both the residual metabolic activity and the change from baseline for the DKFZ-BT314-OFF model (Fig. 2C, Suppl. Figure 3). Representative high-content microscopy images of treated 3D cultures highlight the advantages of fluorescence-based TMRE readouts compared to brightfield imaging, enabling clear discrimination between viable and non-viable regions within the 3D cultures and facilitating the discrimination between cytostatic (same size) and cytotoxic (decreased size) treatment effects (Fig. 2D). Selinexor at 1000 nM and lower demonstrated efficacy at clinically achievable concentrations (C_max_ 1071 nM), and the combination with binimetinib maintained or even enhanced this effect in the tested models (Fig. 2; Suppl. Figure 3). The known combination drugs validated the assay, demonstrating that the BCL-X_L_ inhibitors displayed efficacy across all three models, with a notable impact on viability (measured as residual metabolic activity) observed in the senescent model (DKFZ-BT314_OFF). BRAFi, both alone and in combination with binimetinib, demonstrated a robust cytotoxic response in the BT40 multicellular aggregate model (Fig. 2A, D; Suppl. Figure 3). In the DKFZ-BT314_ON and OFF models, the effects were less pronounced (Fig. 2B–C; Suppl. Figure 3). All three tested mTOR inhibitors inhibited further growth but did not reduce the initial 3D culture size (Suppl. Figure 3). Vinblastine, a chemotherapeutic agent utilized in pLGG treatment, demonstrated efficacy in the proliferating DKFZ-BT314_ON cells. To further evaluate XPO1 inhibition as a combination partner for MAPK pathway inhibitors in pLGG, BT40 cells and the senescence-associated pLGG model BT317_OFF, which harbors a KIAA1549::BRAF fusion, were treated with selinexor in combination with the MEK inhibitor mirdametinib or the ERK inhibitor ulixertinib. In line with our findings in BT314_OFF cells treated with binimetinib or trametinib, selinexor also exhibited single-agent activity in the senescence-associated BT317_OFF model and further enhanced the effects of alternative MAPK pathway inhibitors in both BT40 and BT317_OFF cells (Suppl. Figure 4).

### MEK and XPO1 co-inhibition induces cell death in 3D cultures and complementary 2D assays

**Fig. 3:**
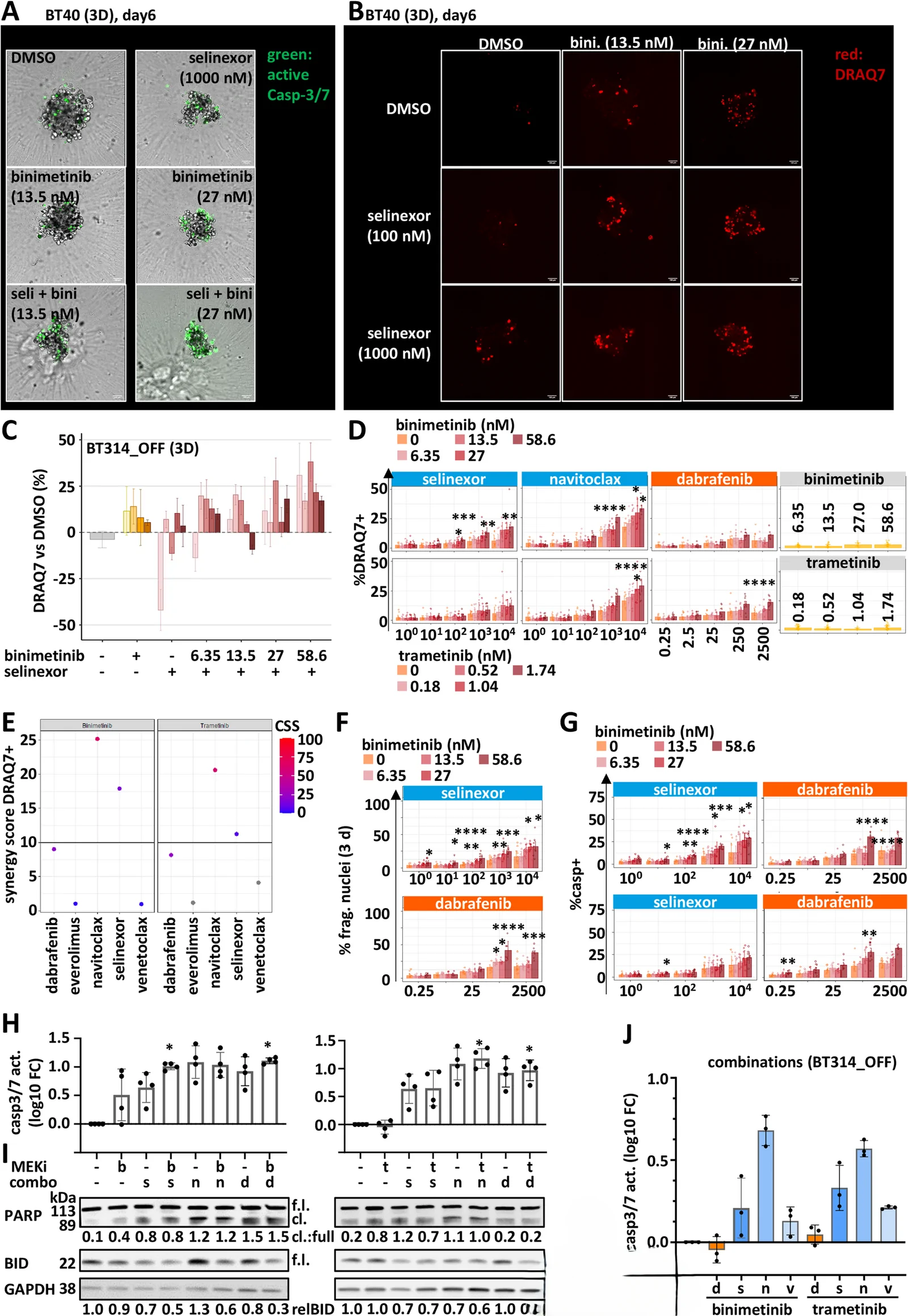
MEKi and selinexor induce cell death in pLGG 3D cultures, with complementary mechanistic analyses in 2D.** A** Representative high-content microscopy images of active caspase-3/7 (CellEvent™ Caspase-3/7 Green Detection Reagent) in BT40 multicellular aggregates after 6 d of the indicated treatments. Green fluorescence indicates cells with activated Caspase-3/7. Merged fluorescence and brightfield images are shown. Scale bars, 100 μm. **B** Representative DRAQ7 images of BT40 multicellular aggregates after 6 d of the indicated treatments. Red fluorescence indicates membrane-compromised and dead cells. Scale bars, 100 μm. **C** DRAQ7-positive cells in 3D-cultured DKFZ-BT314_OFF spheroids after the indicated treatments for 72 h. Data are presented as the percentage of DRAQ7-positive cells relative to DMSO-treated controls. Bars represent the mean, and error bars indicate standard deviation (SD) of replicates. **D** Percentage of DRAQ7-positive cells (late-stage cell death) relative to total cell number. BT40 cells were seeded as a monolayer and treated for 72 h with single agents or in combination with either binimetinib (upper panel) or trametinib (lower panel) at different concentrations (IC_25_, IC_50_, 2x IC_50_, IC_75_). Data represents the mean ± SD of three independent biological replicates with small dots indicating individual wells. All concentrations indicated on the x-axis are expressed in nM. **E** BLISS synergy scores calculated with SynergyFinderPlus based on the percentage of dead cells measured by DRAQ7 staining in **D**. **F** Fragmented nuclei and **G** caspase-positive cells following 72 h treatment in BT40 monolayers. Data represents the mean ± SD of three independent biological replicates with small dots indicating individual wells. All concentrations indicated on the x-axis are expressed in nM. **H** Caspase-3 activity measured using a fluorescent DEVD-based substrate in cell lysates of BT40 cells, seeded as monolayers, after 48–72 h of treatment (cells were collected when they started to detach). Values are normalized to the solvent control. Bars represent the mean ± SD of three biological replicates. Treatments: binimentinib (‘b’, 58.6 nM), trametinib (‘t’, 1.74nM), selinexor (‘s’, 100 nM), navitoclax (‘n’, 100 nM), dabrafenib (‘d’, 250 nM). **I** Western blot analysis of cleaved PARP and BID following 72 h of treatment (same as in **H**). Shown are representative blots from biological replicates. **J** Caspase-3/7 activity measured using a fluorescent DEVD-based substrate in cell lysates of DKFZ-BT314_OFF after 48–72 h of treatment (cells were collected once detachment was observed). Values are normalized to the solvent control. Bars represent the mean ± SD of three biological replicates. D, DMSO; b, binimetinib; t, trametinib; d, dabrafenib; n, navitoclax; s, selinexor; v, venetoclax. Abbreviations: MEKi, MEK inhibitor; IC, inhibitory concentration; CSS, combination sensitivity score

Because the primary screen was performed in 3D culture, we first evaluated whether selinexor-based treatment induced cell death in the same culture format. In BT40 multicellular aggregates, treatment with selinexor alone or together with MEK inhibition increased caspase-3/7 activity and DRAQ7 positivity. In 3D-cultured DKFZ-BT314_OFF spheroids, selinexor and the MEKi combinations likewise increased DRAQ7-positive cells (Fig. 3A-C). These 3D data provide the principal evidence that loss of viable 3D culture area reflects cell death rather than solely reduced metabolic activity. We then used 2D monolayer cultures for complementary mechanistic assays that require efficient recovery of sufficient cell numbers and benchmarked the effect against known interaction partners: The BRAFi dabrafenib was approved for the treatment of low-grade glioma with BRAF^V600E^ mutations in combination with trametinib. The mTORi everolimus was approved for pediatric brain tumors. The BCL-X_L_ affine BH3 mimetic navitoclax (phase 2 clinical trial) and the BCL2-selective venetoclax, which has been approved for the treatment of certain blood cancers. (Fig. 3; Suppl. Figure 5). In BT40 monolayers, DRAQ7 staining (Fig. 3D, E), trypan blue exclusion (Suppl. Figure 6 **A**), nuclear fragmentation (Fig. 3F; Suppl. Figure 6B), and caspase-3/7 activity (Fig. 3G) demonstrated that selinexor induces cytotoxic cell death; in several assays, the effect was enhanced by binimetinib or trametinib. The BLISS synergy analysis indicated the presence of synergistic interactions between the MEKi combinations and navitoclax and selinexor (Fig. 3E). PARP and BID cleavage provided additional mechanistic support in 2D lysates. The 2D experiments are therefore interpreted as complementary mechanistic analyses and not as substitutes for the 3D treatment-response data. Taken together, the results show that XPO1 inhibition, alone or in combination with MEK inhibition, induces cell death in the tested pLGG 3D cultures, while the 2D assays further characterize the associated apoptotic response. Furthermore, the effect was comparable to the combination treatment with navitoclax or dabrafenib, which have been described as effective in previous studies.

### XPO1 inhibition, alone or with MEK inhibition, suppresses growth of 3D cultures and patient-derived fresh tissue tumoroids in vitro; exploratory assessment in zebrafish xenografts in vivo

**Fig. 4:**
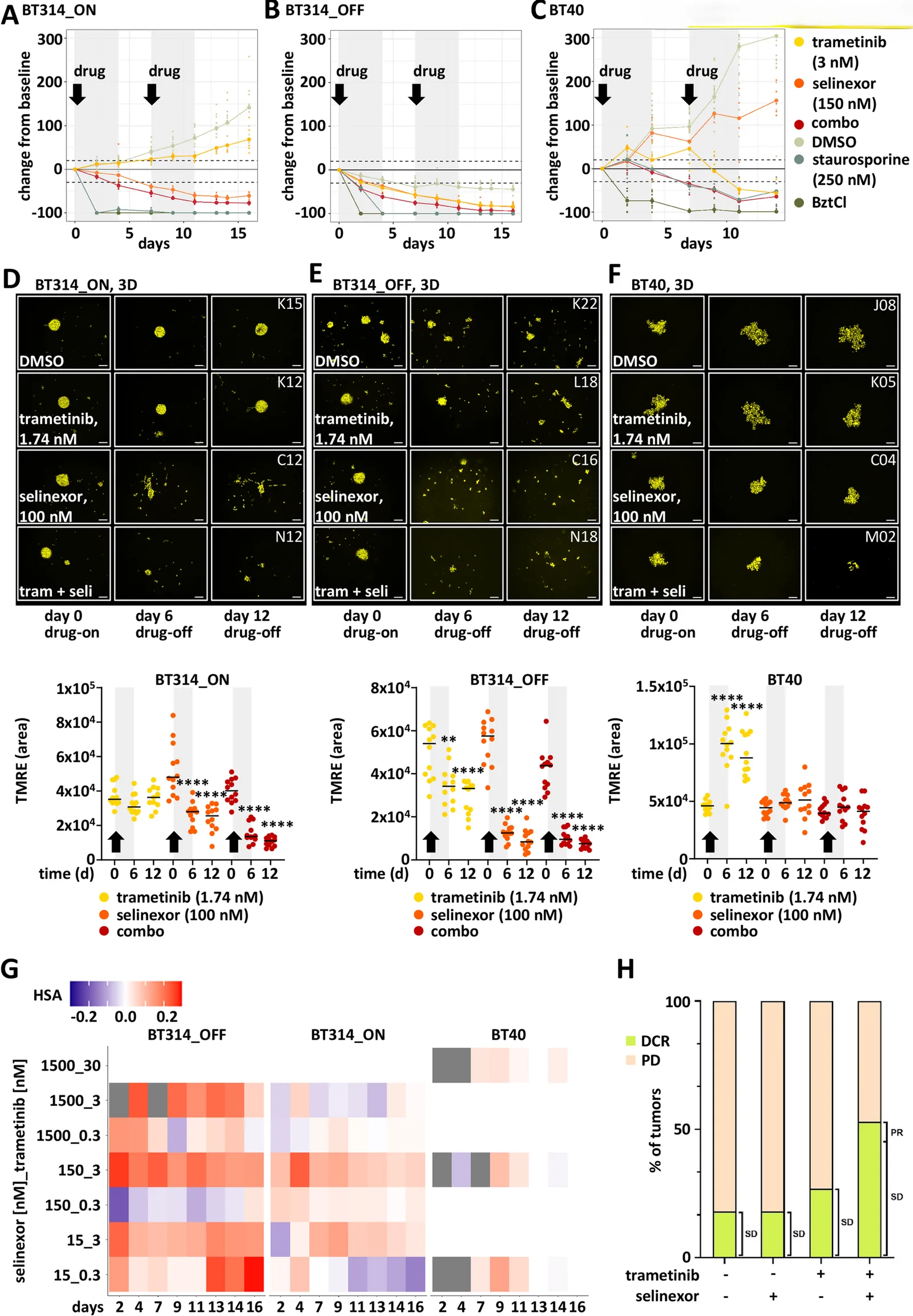
Selinexor and MEKi suppress pLGG 3D culture growth in vitro; exploratory assessment in zebrafish xenografts.** A-C** DKFZ-BT314_ON spheroids (**A**), DKFZ-BT314_OFF spheroids (**B**), and BT40 multicellular aggregates (**C**) treated as indicated (treatment on indicated as grey-shaded areas). Curves depict mean change from baseline of TMRE-positive 3D culture area across five technical replicates, horizontal lines represent response thresholds based on RECIST-like criteria. **D-F** Representative images and quantification of DKFZ-BT314_ON spheroids (**D**), DKFZ-BT314_OFF spheroids (**E**), and BT40 multicellular aggregates (**F**) cultured for up to 12 d and treated as indicated during the first 6 d. **G** HSA interaction scores calculated from inhibition of 3D culture growth. **H** Change in tumor volume in BT40 zebrafish embryo xenografts from day 1 to day 3 post-injection. Embryos (at least 10 per group) were treated for 48 h with solvent control, 1 µM selinexor, 20 nM trametinib, or the combination. The overall group comparison did not reach statistical significance (ANOVA, *p* = 0.058). Abbreviations: MEKi, MEK inhibitor; TMRE, tetramethylrhodamine ethyl ester; HSA, Highest Single Agent

As XPO1 inhibition with selinexor, alone and in combination with MEK inhibition, showed activity across all three pLGG 3D culture models, we further characterized the durability of this response using alternating on- and off-treatment phases to simulate the clinical setting. Viable 3D culture area was monitored relative to baseline. Selinexor alone produced a sustained reduction (PR) in the DKFZ-BT314_ON/OFF models, and trametinib plus selinexor reduced viable area in all three models (Fig. 4A–C). With trametinib single treatment the 3D culture sizes reached SD, when the drug was on (grey areas), but the 3D cultures continued to grow reaching PD, when the treatment was paused (white area), indicating a potential re-growth capacity (Fig. 4A, C). To further challenge the robustness of the response, cells were treated once and the medium was refreshed after 6 d without re-dosing. While trametinib-treated DKFZ-BT314_ON 3D cultures tended to regrow (Fig. 4D), selinexor-treated 3D cultures, either alone or in combination with trametinib, maintained their reduced size for up to 12 d across all three models (Fig. 4D–F). More sophisticated assays would be required to demonstrate prevention of regrowth conclusively [38]. HSA analysis indicated combinatorial activity across clinically achievable concentrations, most prominently in senescent DKFZ-BT314_OFF cells (Fig. 4G). We additionally explored the treatment in BT40 zebrafish embryo xenografts.

We transplanted fluorescently labeled BT40 cells into the yolk sac of zebrafish embryos and started treatment 24 h after injection for 48 h with solvent control, trametinib, selinexor and combination (zebrafish-MTD trametinib 100 nM, zebrafish-MTD selinexor 1 µM; [29]). Whereas more than 80% of tumors in the solvent control group showed progressive growth (31 of 37 tumors), progression was observed in only 7 of 15 tumors in the combination-treatment group. Thus, after only 48 h of treatment, the combination numerically attenuated tumor growth and achieved a disease control rate of 53% (Fig. 4H; Suppl. Figure 7). Although the difference did not reach statistical significance (ANOVA, *p* = 0.058), the numerical reduction in tumor growth may indicate an early treatment effect within the brief 48-hour exposure period. Nevertheless, these findings remain descriptive and hypothesis-generating and do not establish in vivo efficacy.

**Fig. 5:**
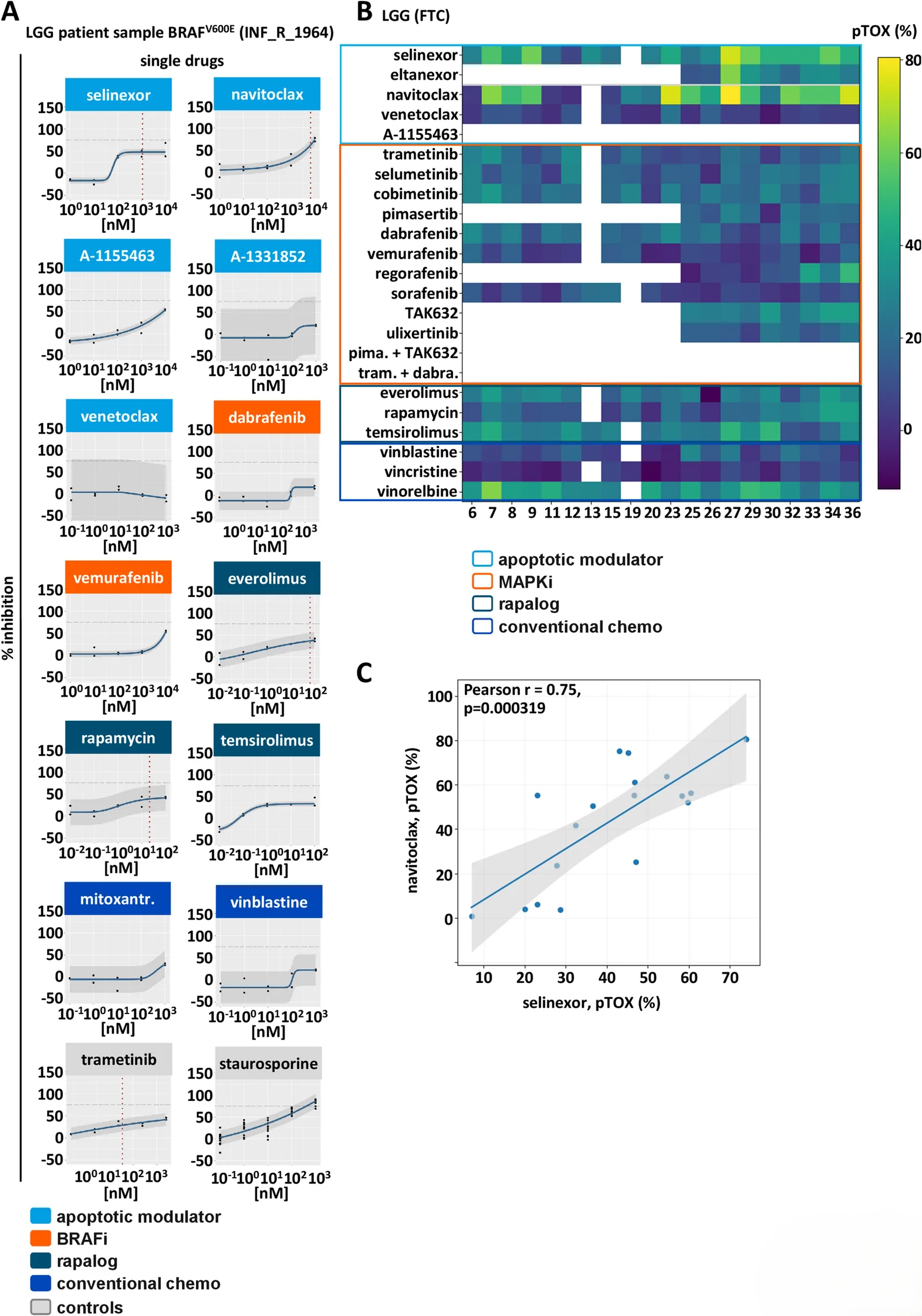
Ex vivo DSP of pLGG patient-derived tumoroid cultures.** A** Dose-response curves displaying the percent inhibition of metabolic activity in a patient-derived short-term culture (INF_R_1964) collected through the INFORM program. Fresh-tissue tumoroids were directly screened and treated for 72 h with the indicated drugs. **B** Heatmap depicting % inhibition at respective Cmax (‘pTOX’) across 20 pediatric low-grade glioma samples collected within the LOGGIC Core BioClinical Data Bank. Patient-derived ex vivo fresh tissue tumoroid cultures (FTCs) were directly established and exposed for 72 h to the indicated drugs. Drug sensitivity was assessed by measuring metabolic activity using CellTiter-Glo 2.0. A-1,155,463: no clinical C_max_ available; pMAX shown in Supplementary Fig. 8A. **C** Scatter plot showing the correlation between selinexor and navitoclax responses across samples for pTOX. Each dot represents one sample. The blue line indicates the linear regression fit, and the shaded grey area represents the 95% confidence interval of the regression line estimated by bootstrap resampling. Pearson correlation coefficients (r) and corresponding p-values are shown. Abbreviations: DSP, Drug sensitivity profiling; pLGG, pediatric low-grade glioma; INFORM, INdividualized therapy FOr high-Risk childhood Malignancies; FTC, fresh tissue tumoroid cultures; LOGGIC, Low-Grade Glioma in Children

Notably, the ex vivo drug sensitivity profile of a fresh tissue tumoroid culture derived from a LGG patient sample harboring a BRAF^V600E^ mutation obtained through the international precision oncology program INFORM [59] was analyzed to confirm the sensitivity of the culture to selinexor and in comparison, with well-known treatments (e.g., navitoclax, dabrafenib, and mTORi). The analysis confirmed sensitivity to selinexor, with approx. 50% inhibition at C_max_ (Fig. 5A). Furthermore, we applied the drug sensitivity profiling (DSP) to 20 fresh tissue pLGG samples (Suppl. Table 1), collected and tested within the LOGGIC Core BioClinical Data Bank. Marked inhibition (> 50%) at the respective C_max_ (pTOX) was observed for selinexor in 5 of 20 samples (25%), making it the second most potent drug after navitoclax (Fig. 5B); this was further reflected by the high correlation between selinexor and navitoclax responses (Fig. 5C). As corresponding clinical pharmacokinetic data (C_max_) are not available for preclinical compounds such as A-1,155,463, the maximal percentage inhibition achieved at the highest concentration tested was additionally evaluated. This analysis yielded results comparable to those obtained using clinically relevant Cmax values (Suppl. Figure 8A, B). Comparison of all correlations in a matrix revealed the highest correlation between XPO1 inhibition and BCL-X_L_ antagonists (Suppl. Figure 8C). In conclusion, selinexor alone and in combination with MEKi suppressed viable 3D culture area in cell-line models and showed activity in patient-derived fresh tissue cultures in vitro and ex vivo.

### The combination of selinexor and MEKi diminishes anti-apoptotic BCL-2 family member MCL-1

**Fig. 6:**
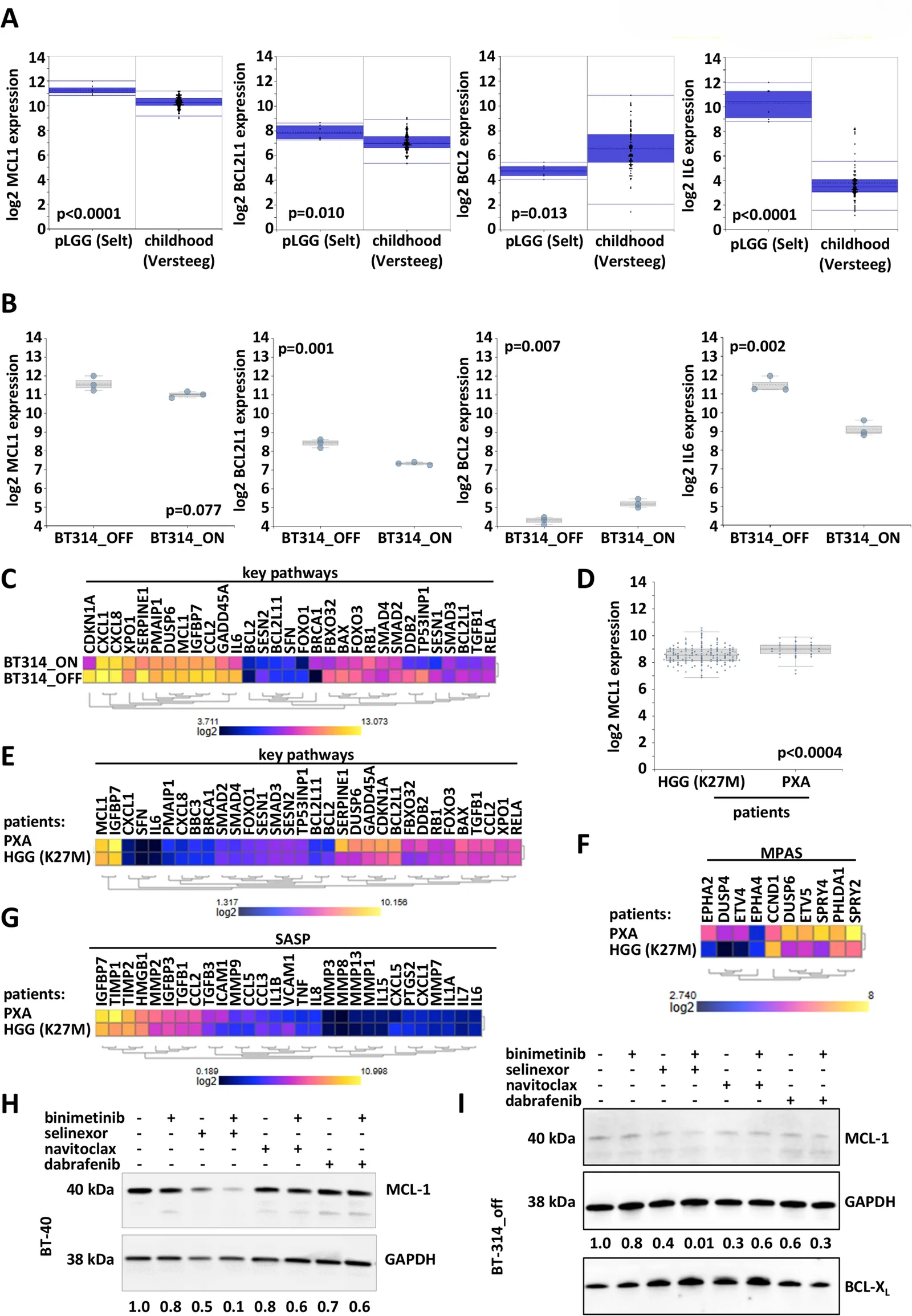
Expression of key pathway genes in pLGG.** A** Gene expression of senescent LGG models (“Selt”, *n* = 6) and predominantly proliferative pediatric tumor cell lines (“Versteeg”, *n* = 86), obtained from the R2 Genomics Analysis and Visualization Platform (http://r2.amc.nl). Statistics were calculated by ANOVA. **B-C** Gene expression data in DKFZ-BT314 senescent and proliferating models from the R2 Genomics Analysis and Visualization Platform (http://r2.amc.nl), “Exp PLGG (BT314), Selt”. Statistical analysis was performed using ANOVA. **C** Heatmap of key pathway genes showing unsupervised clustering based on log₂-normalized expression values. **D-G** Gene expression data in INFORM patient samples from the R2 Genomics Analysis and Visualization Platform (http://r2.amc.nl), “Tumor Pediatric Inform - Pilot / Registry − 2900 - tpm - gencode19”. Statistical analysis was performed using ANOVA. **E-G** Heatmaps of key pathway genes (**E**), MAPK Pathway Activity Score (MPAS) genes (**F**) and SASP genes (**G**) showing unsupervised clustering based on log₂-normalized expression values. **H-I** Western blot analysis of relative MCL-1 protein levels in BT40 (**H**) and DKFZ-BT314_OFF (**I**) cells following 72 h treatment as indicated (58.6 nM binimetinib, 100 nM selinexor, 100 nM navitoclax, 25 nM dabrafenib, 1000 nM staurosporin). GAPDH served as a loading control. Numbers indicate the expression relative to solvent (-) and normalized to GAPDH expression. Shown are representative blots from biological replicates. Abbreviations: BCL-2, Anti-apoptotic B-cell lymphoma 2; pLGG, pediatric low-grade glioma; MCL-1, Myeloid leukemia cell differentiation protein; GAPDH, Glyceraldehyde 3-phosphate dehydrogenase

Exportin 1 (*XPO1*) is a nuclear receptor exporter that plays a role in the active transport of a number of cargo proteins, including prominent cancer related proteins, such as p53, FOXO3, IkB and p21 [28]. Additionally, it interacts with various mRNA-binding chaperones, such as elF4E, which facilitates the nuclear exit of subsets of mRNA encoding oncogenes, including MCL-1 and BCL-2 [76]. Gene expression analysis with datasets available on R2 (R2: Genomics Analysis and Visualization Platform (http://r2.amc.nl)) revealed that the rather senescent MAPK-driven LGG models (‘Selt’), as indicated by IL6 upregulation, express significantly more *MCL1* and BCL-X_L_ (*BCL2L1*), but less *BCL2* compared to a panel of mostly proliferative pediatric tumor cell lines (‘Versteeg’), including medulloblastomas, neuroblastomas, leukemia and sarcomas (Fig. 6A). In the LGG models, the DKFZ-BT314 cells demonstrate high expression of *MCL1* in the OFF (senescent) and ON mode (Fig. 6B). Senescent DKFZ-BT314_OFF cells are characterized by significantly higher expression of BCL-X_L_ (*BCL2L1)* but lower *BCL2* expression compared with the proliferative ON state (Fig. 6B). Analysis of a panel of key pathway genes (Suppl. Data Table) revealed upregulation of genes involved in SASP (*CXCL8*,* IL6*), senescence (*SERPINE1*,* CDKN1A*), FOXO1/3, NF-κB (*RELA*), TGF-β, and p53 signaling (*GADD45A*,* TP53INP1*) and MAPK activation signature (MPAS) [77] in DKFZ-BT314_OFF cells compared with the ON model (Fig. 6C; Suppl. Figure 8D, E). In contrast, transcriptional activation of pro-apoptotic BH3-only genes (*BCL2L11*/BIM and *PMAIP1*/NOXA) was absent, indicating that BH3-mediated apoptosis is rather suppressed in oncogene-induced senescence. To further extend these observations using patient-derived material, the same panel of key pathway genes was analyzed in the INFORM dataset, comparing proliferative K27M-mutant high-grade glioma patients with PXA patient samples. PXA samples showed significantly higher expression of *MCL1* (Fig. 6D) as well as increased expression of senescence markers (*SERPINE1*, *CDKN1A*), p53 pathway genes (*GADD45A*, *DDB2*), and *BCL2L1*, whereas SASP markers (*IL6*, *IL8*) were not upregulated (Fig. 6E). Furthermore, PXA samples were characterized by upregulation of the MAPK activation signature (MPAS) [77] (Fig. 6F). A more detailed analysis of the SASP signature, as published by Coppé [17], revealed upregulation of selected SASP factors, including *TIMP1/2*, *MMP2*, *TGFB3*, and *ICAM1*, in PXA patient-derived samples compared with K27M-mutant high-grade glioma samples (Fig. 6G). Thus, both PA and PXA models share high expression of *MCL1*, *BCL2L1*, *DUSP6*, *SERPINE1*, *IGFBP7*, *GADD45A*, *FBXO32*, and *DDB2*, many of which are transcriptional targets of p53 and FOXO signaling pathways and are known cargos of XPO1 (Suppl. Data Table). Furthermore, MCL-1 protein levels were reduced in PXA BT40 and PA DKFZ-BT314_OFF cells following treatment the XPO1 inhibitor selinexor, either alone or in combination with MEKi binimetinib (Fig. 6H, I). Administering selinexor alone resulted in a 0.5-fold reduction in MCL-1 levels (BT-40) or a 0.4-fold reduction (DKFZ-BT314_OFF). Combining binimetinib and selinexor led to a significantly greater reduction in MCL-1 levels, to 0.1-fold (BT-40) or 0.01-fold (DKFZ-BT314_OFF), compared to untreated cells (Fig. 6H, I). Notably, MCL-1 protein levels were relatively low, whereas BCL-X_L_ was more prominently expressed, consistent with the gene expression data described above. However, unlike MCL-1, BCL-X_L_ expression remained unchanged following selinexor treatment (Fig. 6I). In conclusion, combined XPO1 and MEK inhibition reduced levels of the anti-apoptotic protein MCL-1, thereby potentially supporting the induction of cell death.

## Discussion

Although the biological background of most pLGGs, involving activation of the MAPK pathway, is well understood, clinical treatment remains challenging. As demonstrated in various clinical studies and confirmed by our cell culture experiments, complete responses are rarely achieved through MEKi or BRAFi monotherapy. Furthermore, rapid tumor regrowth after treatment discontinuation in a subset of patients underscores the limitations of these approaches [24, 39, 54, 67, 72]. Consequently, additional vulnerabilities need to be identified to enable cytotoxicity resulting in increased depth of response and more durable tumor control. In this study, we systematically evaluated the therapeutic potential of combining MEK inhibitors with targeted and chemotherapeutic agents in BRAF^V600E^ mutant pLGG models, screening 87 clinically relevant drugs and identifying cytotoxic combination partners for MEKis.

The need for and advantages of, functional drug sensitivity profiling for precision oncology programs, in addition to approaches that solely identify genetically matched molecular targeted therapies [42, 59, 75], has already been demonstrated [46, 50, 57]. Traditional screening approaches [13, 27, 47] often rely on bulk metabolic assays, such as CTG, which fail to discriminate between cytostatic and cytotoxic responses [15, 78]. The EXALT study demonstrated that results from screening adult hematological cancers with 139 drugs using high-content microscopy and automated image analysis are clinically applicable to identify responders [40]. We identified changes in TMRE-positive 3D culture area, as measured by high-content imaging, as a robust endpoint for quantifying responses to drug treatment. TMRE area measurement provided additional information beyond brightfield imaging, enabling discrimination between viable and dead regions within the 3D cultures and facilitating the exclusion of cell debris during automated image analysis. Because TMRE is non-toxic throughout treatment, growth kinetics can be monitored in the same well over time. This is an advantage over destructive endpoint assays, such as the CTG or trypan blue exclusion tests. In our study, this approach allowed us to distinguish between drugs that predominantly inhibit growth, such as mTORi, and those that induce cell death, such as selinexor.

In our study, we identified MEKi combination partners with favorable combinatorial activity that are already well known, such as the FDA-approved combination of dabrafenib and trametinib as a systemic therapy for pediatric LGG harboring a BRAF^V600E^ mutation [7]. Aberrant activation of the MAPK pathway induces the compensatory upregulation of AKT signaling, which subsequently activates the mTOR pathway [2, 34, 48]. This provides the rationale for the clinical evaluation of mTOR inhibitors [73]. PNOC021 is the first pLGG trial to combine trametinib with the mTOR inhibitor everolimus to determine the maximum tolerated dose (NCT04485559). However, the mTORi tested in our models displayed predominantly cytostatic activity. This finding is consistent with previous reports indicating that synergistic effects may depend on anti-angiogenic mechanisms that are not captured by our cell line models [2, 38]. As senescent cells account for up to 60% of the tumor mass [68], it is crucial to investigate therapeutic strategies that specifically target OIS. Compounds with high affinity for BCL-X_L_ (BCL-X_L_i), widely recognized as a therapeutic target in pilocytic astrocytoma, effectively induced senolysis [66], reducing both metabolic activity and 3D culture size in senescent pLGG models in our studies.

Our staged discovery-to-validation strategy identified the blood-brain barrier-penetrant XPO1 inhibitor selinexor as a candidate for further preclinical evaluation. BT40 3D multicellular aggregates served as the initial discovery model, enabling systematic high-content screening of a large drug panel. Following prioritization, the activity of selinexor, alone and in combination with MEK inhibition, was also observed in the DKFZ-BT314_ON/OFF spheroid models. Combined MEK-XPO1 inhibition reduced viable 3D culture area and suppressed regrowth after medium replacement in both proliferative and senescent cultures. Furthermore, ex vivo drug-sensitivity profiling of fresh tumor tissue cultures from 20 patients with pLGG provided independent patient-derived evidence of sensitivity to XPO1 inhibition at clinically achievable concentrations. The convergence of findings across these complementary models indicates that the identified XPO1 vulnerability is not restricted to BT40 cells or to their irregular aggregate morphology.

The zebrafish xenograft experiment was limited to a treatment period of 48 h, which is substantially shorter than the multi-week treatment schedules typically employed in mammalian in vivo efficacy studies. Although comparisons across experimental systems must be made cautiously, this exposure period was also shorter than the 6d interval after which the most pronounced effects became apparent in our cell-culture experiments. The brief duration of the assay may therefore not capture treatment effects that require longer exposure. The numerical attenuation of tumor growth and disease control rate of 53% observed after 48 h provide a hypothesis-generating signal of early biological activity; however, the difference did not reach statistical significance (ANOVA, *p* = 0.058) and does not establish in vivo efficacy. Longer-term, adequately powered animal studies are required to determine the magnitude and durability of the treatment effect.

A remaining limitation is the small number of established pLGG 3D models and their model-dependent architecture. BT40 cells formed stable, irregular multicellular aggregates rather than uniformly compact, highly circular spheroids. Although the concordant activity observed in the moderate compact DKFZ-BT314 spheroid models and fresh patient-derived tissue cultures reduces the likelihood that the identified XPO1 vulnerability is specific to BT40 morphology, additional genetically and histologically diverse pLGG models will be required to establish the broader generalizability of the findings, particularly for combined MEK-XPO1 inhibition.

Exportin 1 (*XPO1*) is responsible for the nuclear-to-cytoplasmic transport of RNA species and over 200 proteins, including tumor suppressor proteins such as tumor protein p53 (*TP53*) and retinoblastoma protein (*RB*), and oncogenes such as *c-ABL* and *YAP1*. Given its role in the transport of numerous cargo proteins, XPO1 plays a pivotal role in regulating the cell cycle and cellular proliferation [3]. Selinexor (KPT-330) and related KPT-SINE compounds bind covalently to the cargo-binding groove of XPO1, thereby inhibiting its function. This causes nuclear retention of tumor suppressors and cell cycle regulators, as well as reduced cytoplasmic export of mRNAs encoding oncogenes [53], which in turn induces growth arrest and apoptosis [8, 79]. This control mechanism has been specifically demonstrated for MCL-1, a key anti-apoptotic BCL-2 family member that is particularly important for the survival of senescent tumor cells [71]. We confirmed this by gene expression analyses showing generally higher *MCL1* expression in MAPK-driven PA and PXA models/patient samples compared to other pediatric tumor cell lines or HGG patient samples. Selinexor treatment reduced MCL − 1 protein levels in BT40 cells, consistent with previous reports, as *MCL1* mRNA translation is regulated by the XPO1-dependent factor eIF4E [19, 26]. In our models, this effect was further enhanced when selinexor was combined with MEK inhibitors such as binimetinib, which is in line with a previous study, demonstrating that MAPK activation directly regulates BCL2 family transcription in pLGG cell lines [69]. However, overall MCL-1 levels were rather low in the BT314_OFF model. Another relevant mechanism involves nuclear retention of the inhibitor of NF-κB (IκB), leading to depletion of active nuclear factor kappa-light-chain-enhancer of activated B cells (NF-κB) [36, 43]. Among its multiple functions, NF-κB has been shown to upregulate MCL-1 [43] and to promote expression of proinflammatory cytokines such as IL-6 [36]. IL-6, in turn, is a central component of the senescence-associated secretory phenotype (SASP), where it maintains OIS through NF-κB- dependent signaling [12, 32]. Furthemore, XPO1 inhibition may promote nuclear retention of p53 and FOXO (among other cargos), potentially restoring apoptotic competence in these models. Pro-apoptotic FOXO3 and p53 target genes are e.g., *BCL2L11* (BIM) and *PMAIP1* (NOXA) [41]. Taken together, these findings outline a potential mechanism of action of selinexor in senescent pLGG cells.

Collectively, our findings identify combined MEK and XPO1 inhibition as a rational candidate strategy for further preclinical evaluation in BRAF^V600E^-mutant pLGG. These findings open the discussion for new pLGG treatment options involving SINEs, with second-generation agents such as eltanexor (KPT-8602) now entering clinical evaluation, including a currently ongoing phase 1/2 study (ClinicalTrials.gov NCT02649790) in patients with diverse relapsed or refractory hematologic and solid malignancies [18]. Future research should focus on validating these findings in more advanced in vivo models and clinical studies, as well as on further exploration of the underlying mechanisms to refine these combination treatments.

## Conclusions

We systematically evaluated cytotoxic drug combinations with MEK inhibition in proliferating and senescent pLGG 3D culture models using fluorescence-supported high-content imaging. The assay recapitulated the previously reported activity of combined MEK and BCL-X_L_ inhibition and identified combined MEK and XPO1 inhibition as an alternative candidate strategy. DRAQ7 and caspase-3/7 measurements directly demonstrated treatment-induced cell death in the 3D cultures, while complementary 2D experiments characterized the associated apoptotic signaling. Activity in the compact, patient-derived DKFZ-BT314 spheroid models indicated that the findings were not restricted to the irregular BT40 aggregate morphology. Moreover, ex vivo drug sensitivity profiling of fresh tumor tissue from 20 patients with pLGG provided direct patient-derived evidence of sensitivity to XPO1 inhibition. Mechanistically, selinexor reduced levels of the anti-apoptotic protein MCL-1, with a more pronounced reduction in combination with binimetinib. In the exploratory zebrafish experiment, combination treatment produced a numerical attenuation of tumor growth after only 48 h; however, the difference did not reach statistical significance and therefore does not establish in vivo efficacy. As an FDA-approved drug with demonstrated blood-brain barrier penetration, selinexor warrants further preclinical evaluation in BRAF^V600E^-mutant pLGG. Adequately powered, longer-term in vivo studies are required to establish therapeutic efficacy before clinical translation can be considered.

## Supplementary Information

Below is the link to the electronic supplementary material.

Supplementary Material 1

Supplementary Material 2

Supplementary Material 3

Supplementary Material 4

Supplementary Material 5

## Data Availability

The datasets used and/or analysed during the current study are available in R2 (Selt) and for confidential patient data from the corresponding author on reasonable request.

## Abbreviations

BCL-2: Anti-apoptotic B-cell lymphoma 2
BCL-X_L_i: BCL-X_L_ inhibitor
BF: Brightfield
BID: BH3 interacting-domain death agonist
BRAFi: BRAF type I ½ inhibitor
C_max_: Clinical achievable concentration
CNS: Central nervous system
CTG: CellTiter-Glo
DCR: Disease control rate
DSMZ: DNA fingerprinting authentication
DSS_asym_: Drug sensitivity score
DTT: Dithiothreitol
EMA: European medicines agency
ERK: Extracellular signal-regulated kinases
FBS: fetal bovine serum
FDA: United States Food and Drug Administration
FGFR1: Fibroblast growth factor receptor 1
HCl: Hydrochloric acid
HCM: High-content fluorescence microscopy
HDAC: Histone deacetylases
HSA: Highest single agent model
INFORM: INdividualized therapy FOr high-Risk childhood Malignancies
iTReX: interactive Therapy Response eXploration
IκB-α: Inhibitor of kappa B alpha
MAPK: Mitogen-activated protein kinase
MAPKi: MAPK inhibitor
MCA: Multiplex cell line authentication test
McCT: Multiplex cell Contamination Test
MCL-1: Myeloid leukemia cell differentiation protein
MEK: Mitogen-activated protein kinase kinase
MEKi: MEK inhibitor
MIP: Maximal intensity projection
MTD: Maximum tolerated dose
mTOR: mechanistic Target of Rapamycin
mTORi: mTOR inhibitor
NF-κB: nuclear factor kappa-light-chain-enhancer of activated B cells
NF1: Neurofibromin 1
OIS: Oncogene-induced senescence
OS: Overall survival rate
PA: Pilocytic astrocytoma
PARP: Poly (ADP-ribose) polymerase
PD: Progressive disease
PDX: Patient-derived xenograft
PFS: Progression-free survival
pLGG: Pediatric low-grade glioma
PR: Partial response
PVDF: Polyvinylidene fluoride
PXA: Pleomorphic xanthoastrocytoma
RAF: Rapidly accelerated fibrosarcoma
RAFi: RAF inhibitor
RB: Retinoblastoma protein
RECIST: Response evaluation criteria in solid tumors
SASP: Senescence-associated secretory phenotype
SD: Stable disease
SDS: Sodium dodecyl sulfate
SINE: Selective inhibitor of nuclear export
SOC: Standard of care
TMRE: Tetramethylrhodamine ethyl ester
TP53: Tumor protein p53
WHO: World Health Organization
XPO1: Exportin 1
XPO1i: Exportin inhibitor
