# Supplementary material for: Cytotoxic activity of selinexor in BRAFV600E-driven pediatric low-grade glioma 3D cultures
Source: Acta Neuropathol Commun. 2026 Oct 22;14:220. doi: 10.1186/s40478-026-02461-1 (PMC13689195; doi:10.1186/s40478-026-02461-1)
Supplement: Supplementary file 1 [file 40478_2026_2461_MOESM2_ESM.pdf]

BT40 uncropped – replicate 1

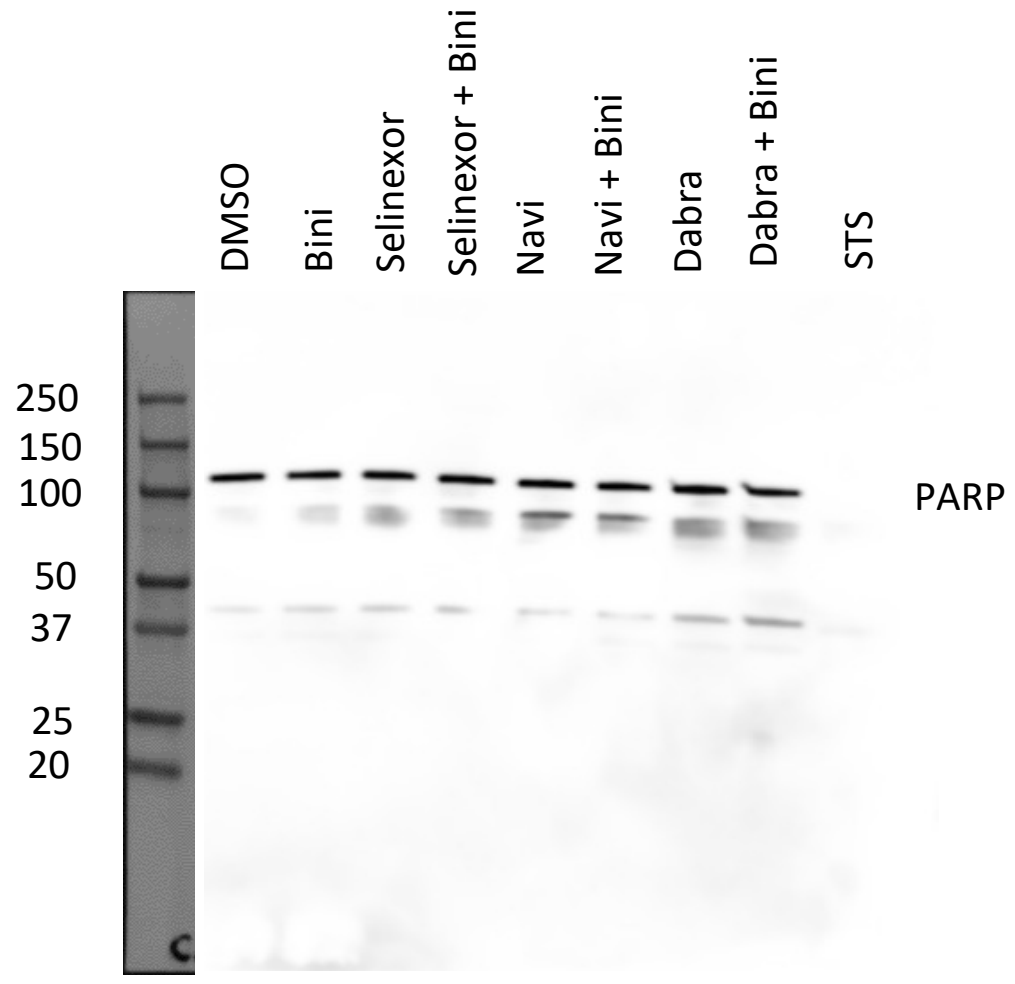

PARP; 1:1000 83/113kDa in 2%Milk in TBS-T
o.n. 4°C
goat anti mouse 1:50.000; RT 1h

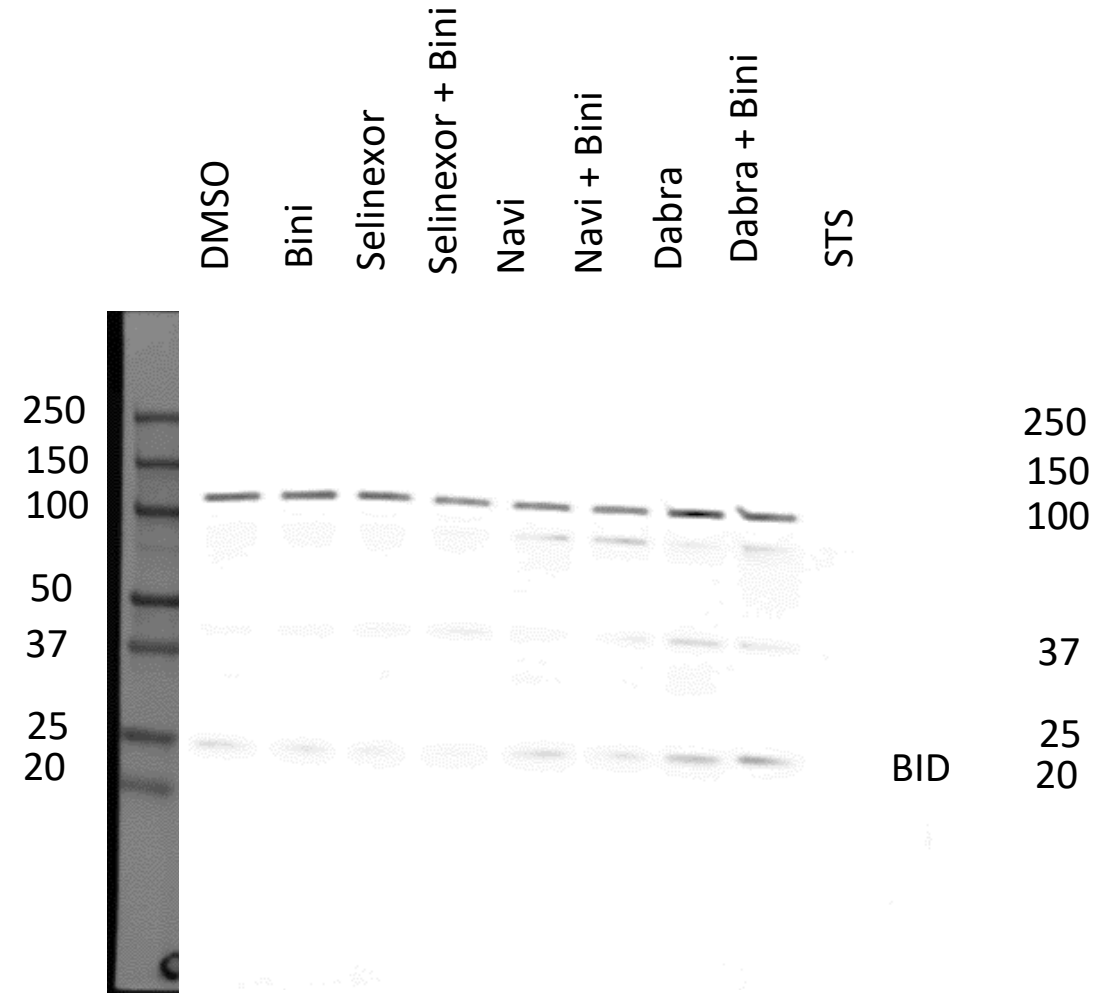

BID; 1:1000 22/15kDa in 2%Milk in TBS-T
o.n. 4°C
goat anti rabbit 1:50.000; RT 1h

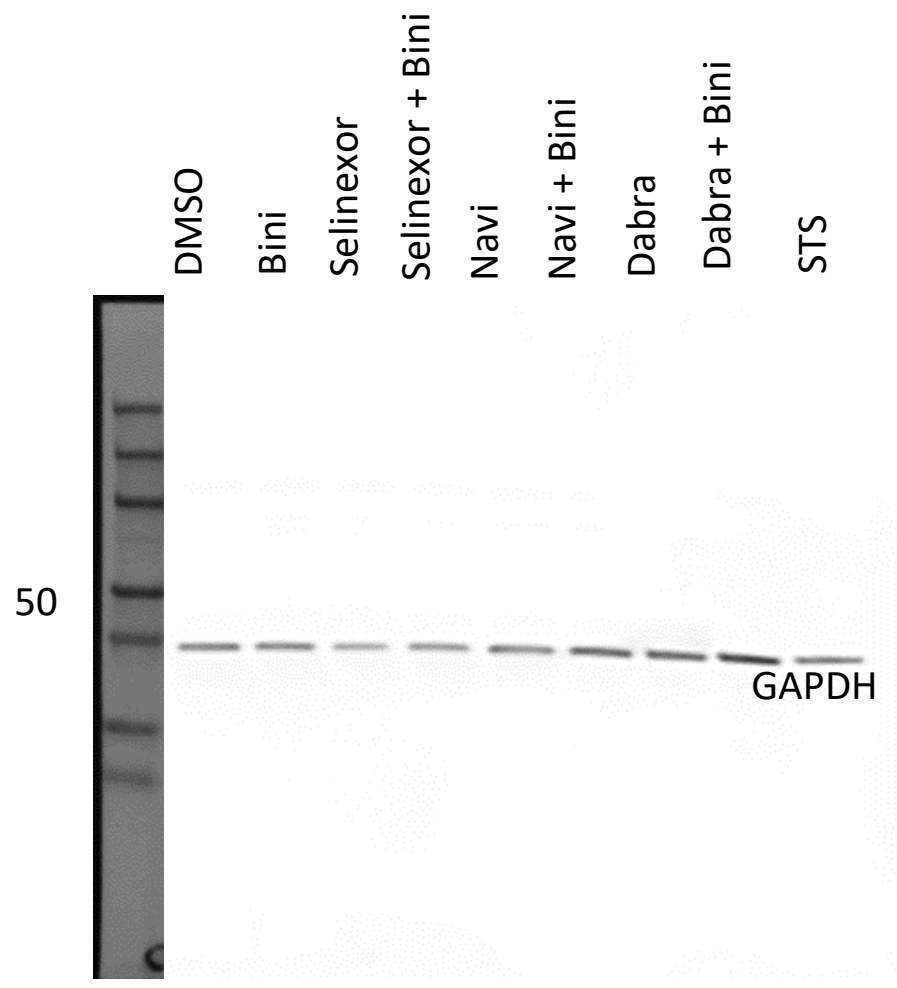

GAPDH; 1:50.000 37kDa in 2%Milk in TBS-T
o.n. 4°C
goat anti mouse 1:50.000; RT 1h

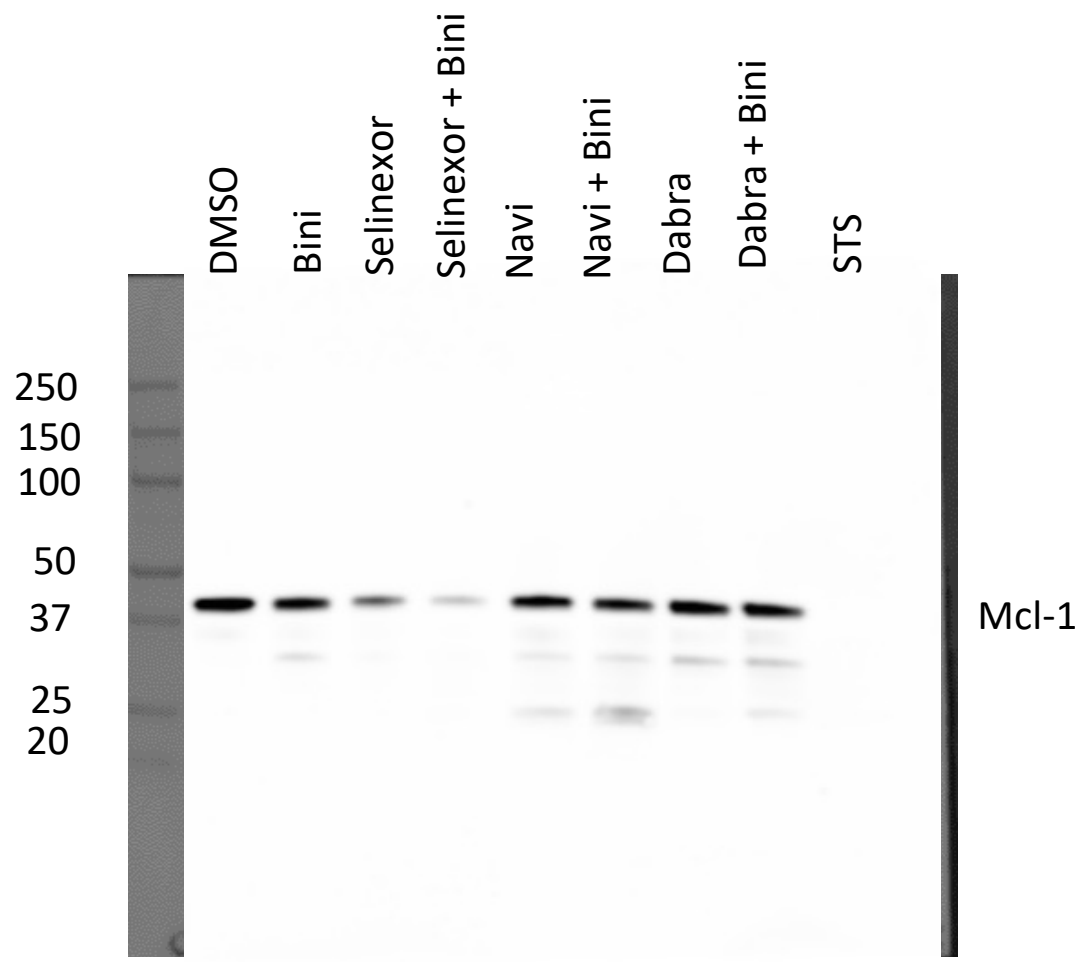

Mcl-1; 1:1000 40kDa in 2%Milk in TBS-T
o.n. 4°C
goat anti rabbit 1:50.000; RT 1h

BT40 uncropped – replicate 2

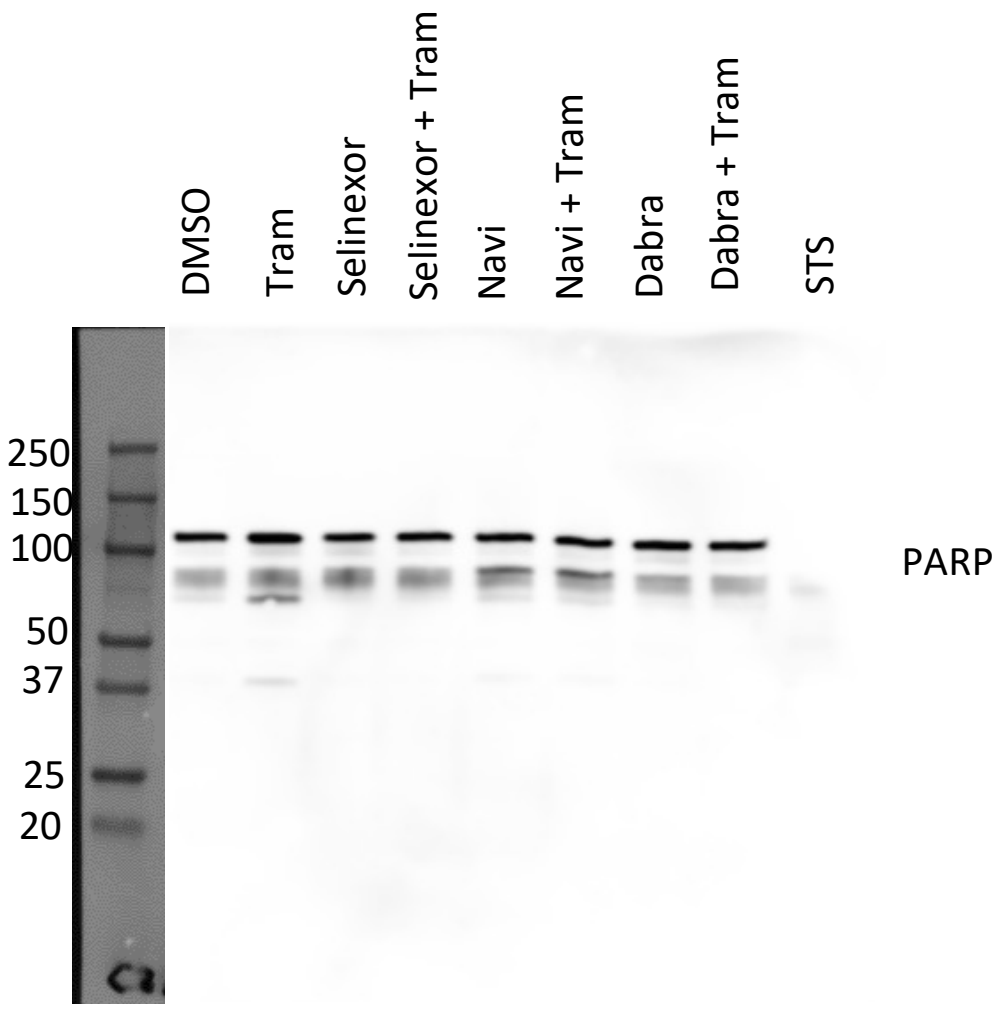

PARP; 1:1000 83/113kDa in 2%Milk in TBS-T
o.n. 4°C
goat anti mouse 1:50.000; RT 1h

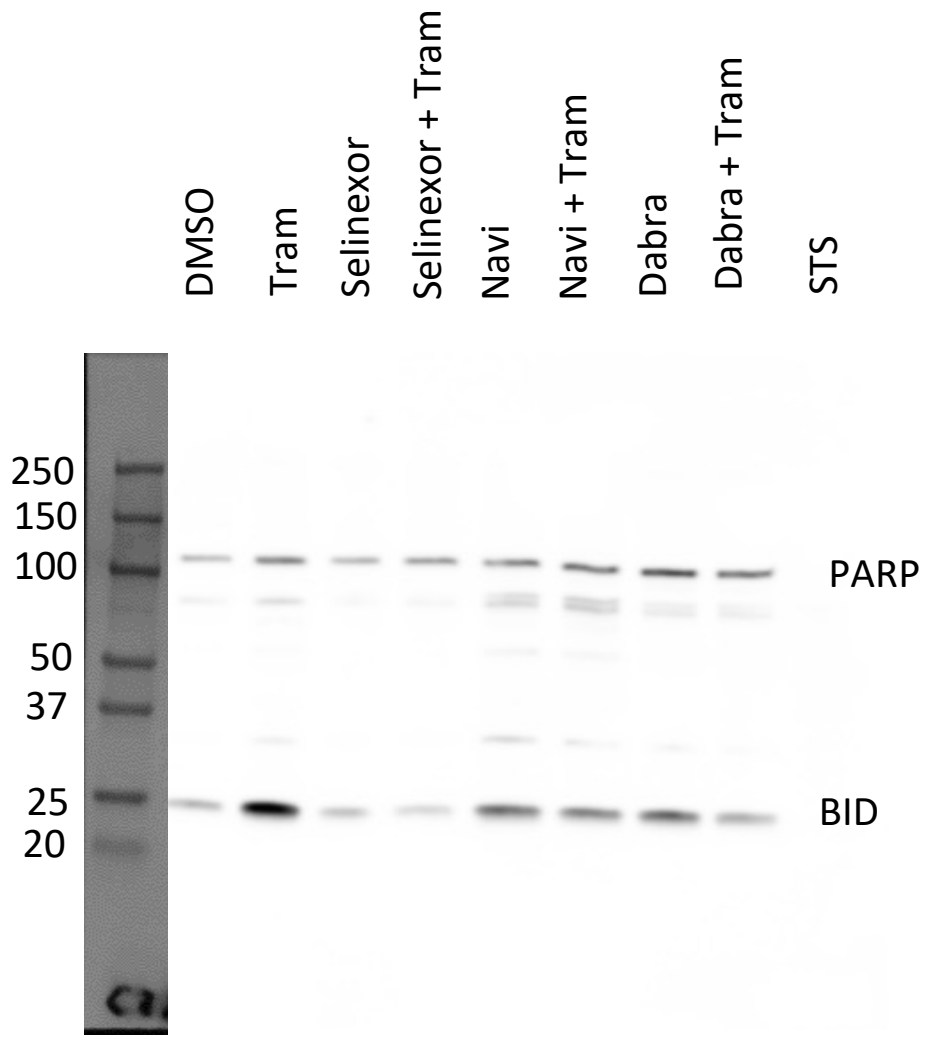

BID; 1:1000 22/15kDa in 2%Milk in TBS-T
o.n. 4°C
goat anti rabbit 1:50.000; RT 1h

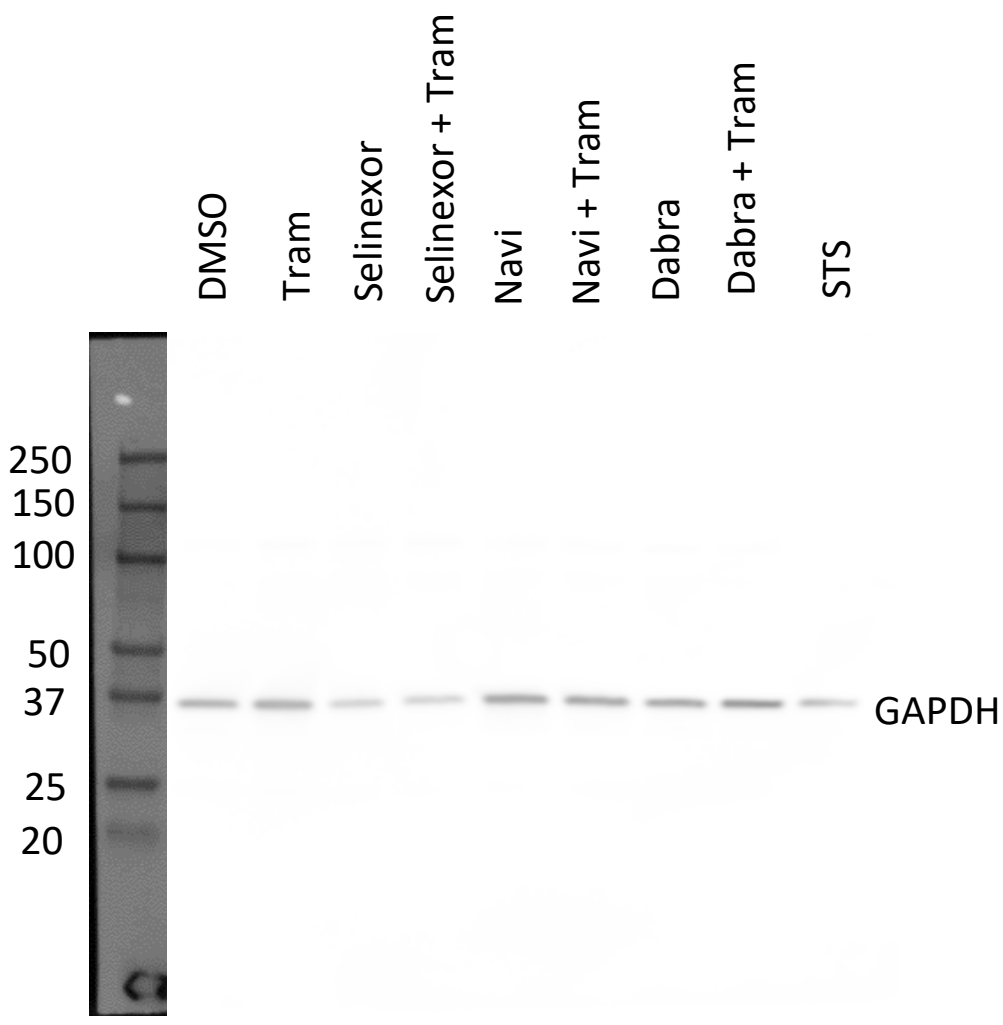

GAPDH; 1:50.000 37kDa in 2%Milk in TBS-T
o.n. 4°C
goat anti mouse 1:50.000; RT 1h

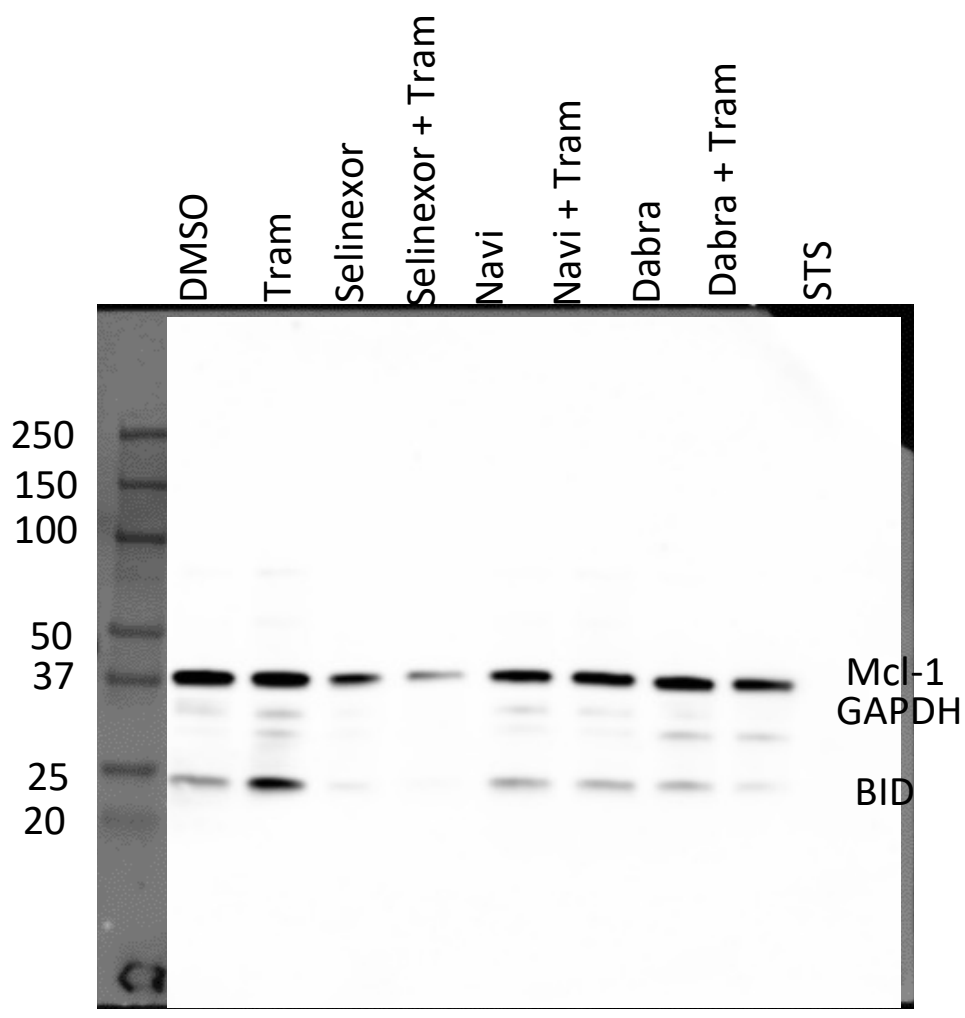

Mcl-1; 1:1000 40kDa in 2%Milk in TBS-T
o.n. 4°C
goat anti rabbit 1:50.000; RT 1h

BT40 uncropped – replicate 3

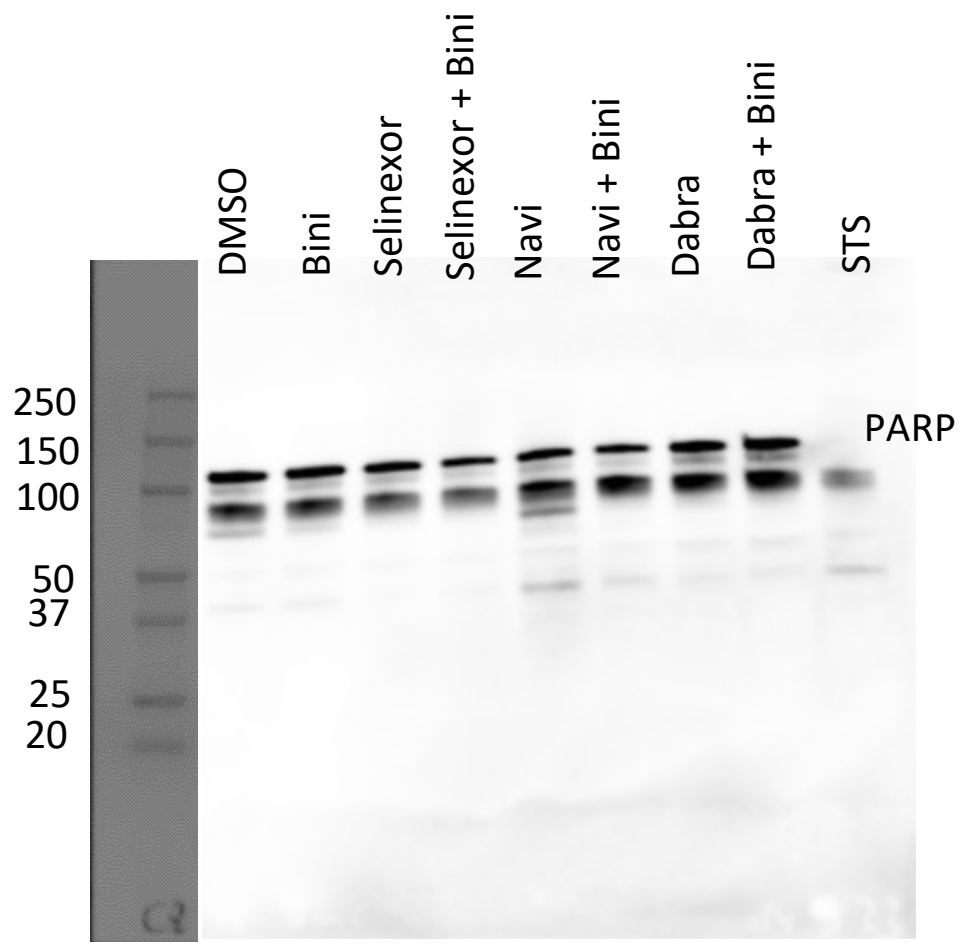

PARP; 1:1000 83/113kDa in 2%Milk in TBS-T
o.n. 4°C
goat anti mouse 1:50.000; RT 1h

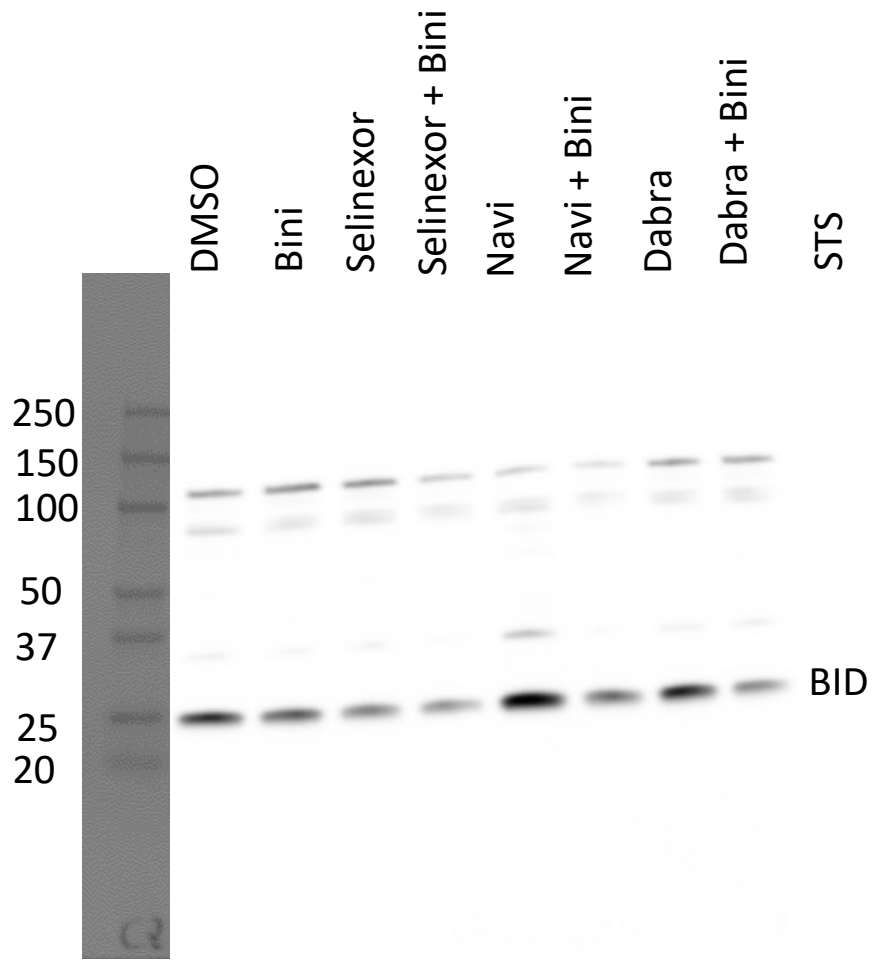

BID; 1:1000 22/15kDa in 2%Milk in TBS-T
o.n. 4°C
goat anti rabbit 1:50.000; RT 1h

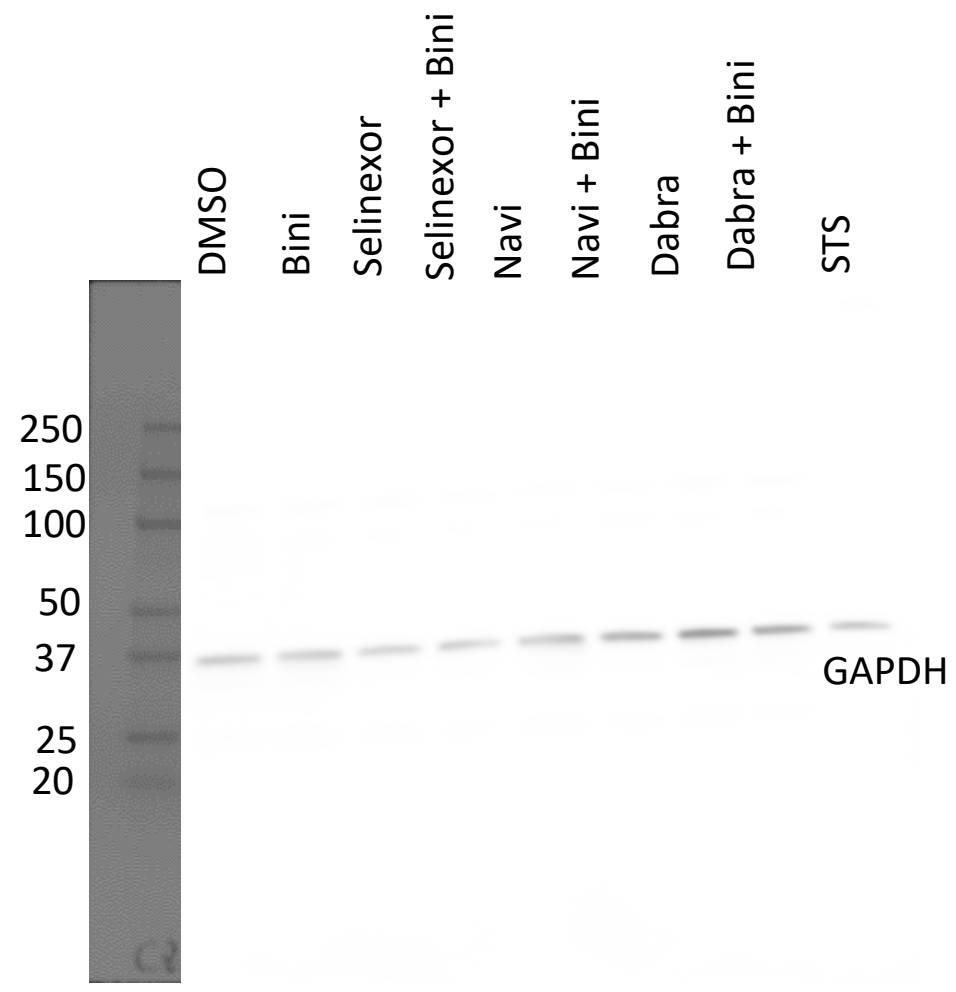

GAPDH; 1:50.000 37kDa in 2%Milk in TBS-T
o.n. 4°C
goat anti mouse 1:50.000; RT 1h

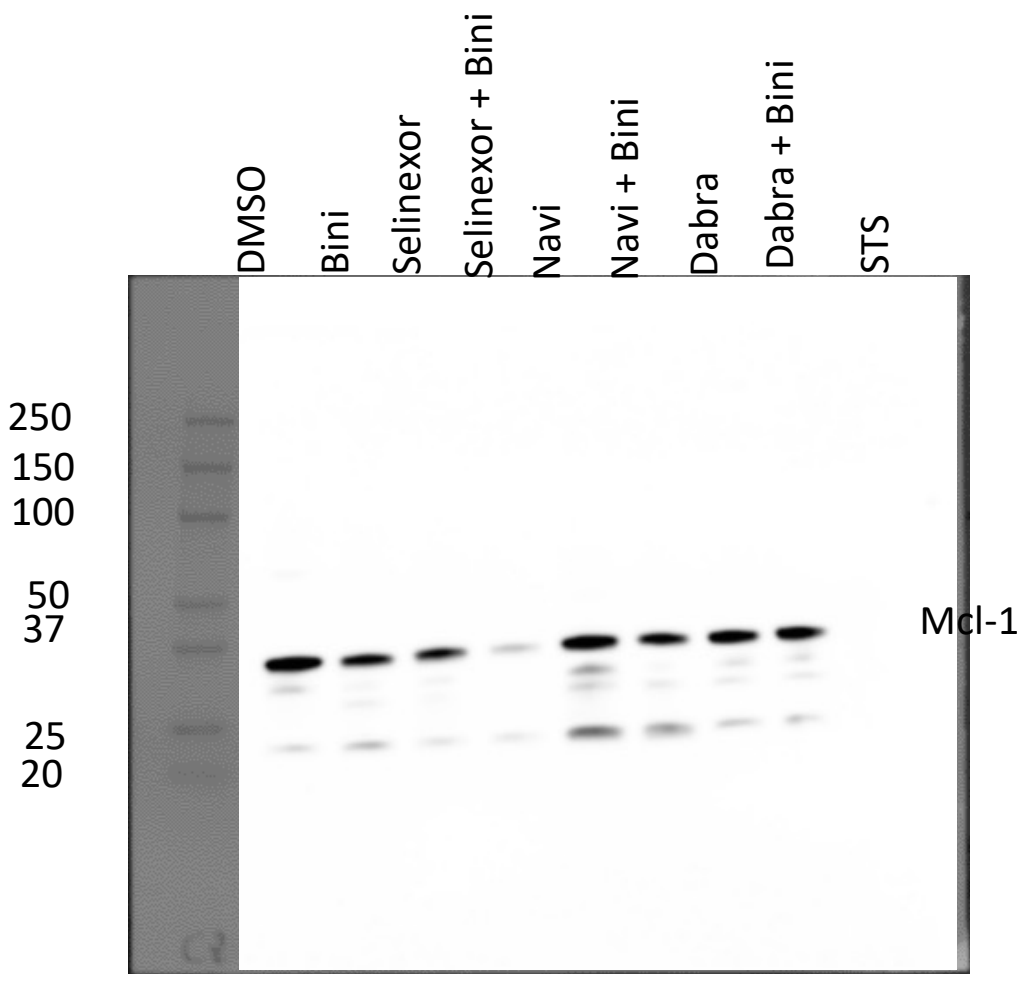

Mcl-1; 1:1000 40kDa in 2%Milk in TBS-T
o.n. 4°C
goat anti rabbit 1:50.000; RT 1h

BT40 uncropped – replicate 4

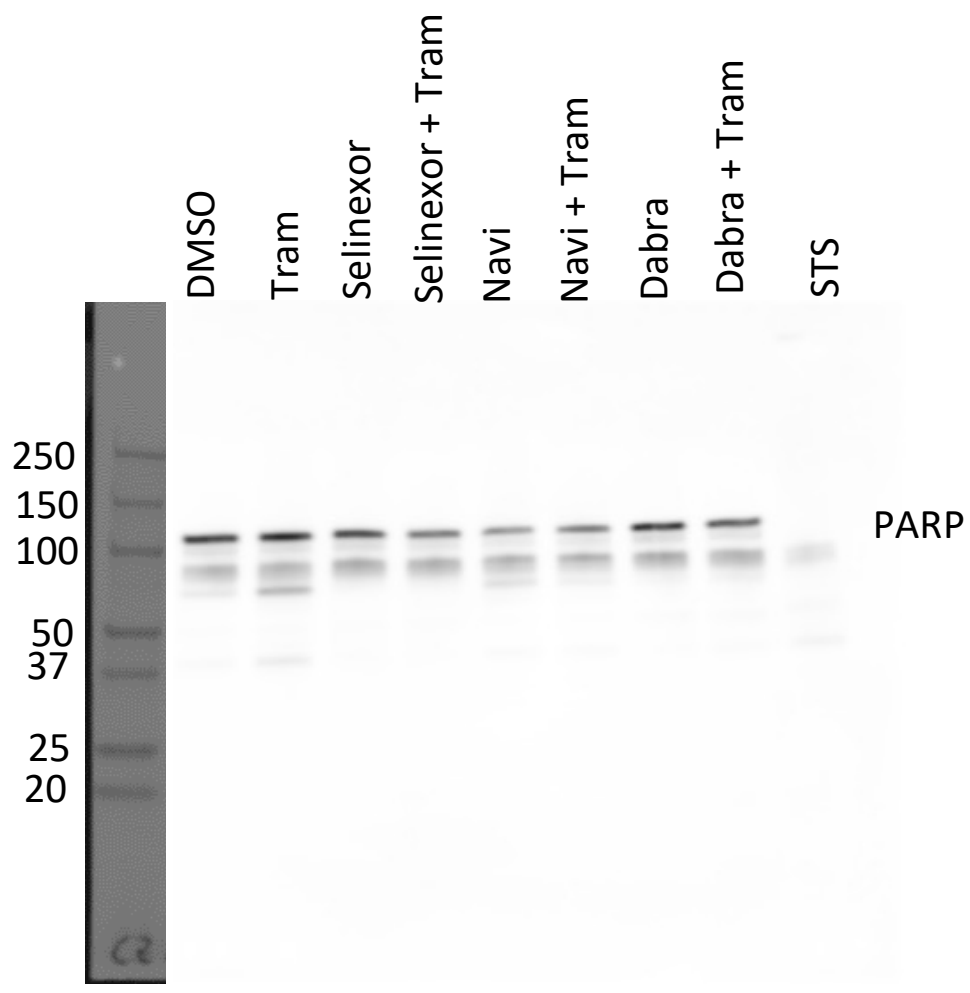

PARP; 1:1000 83/113kDa in 2%Milk in TBS-T
o.n. 4°C
goat anti mouse 1:50.000; RT 1h

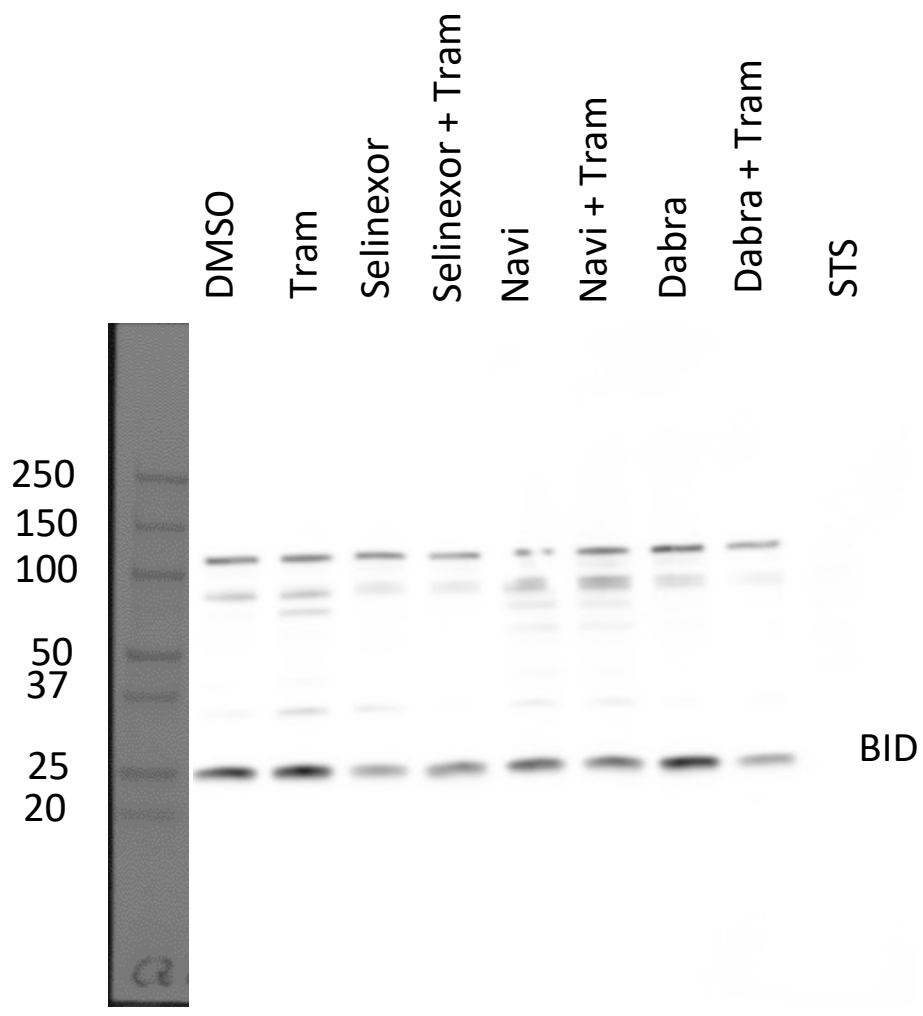

BID; 1:1000 22/15kDa in 2%Milk in TBS-T
o.n. 4°C
goat anti rabbit 1:50.000; RT 1h

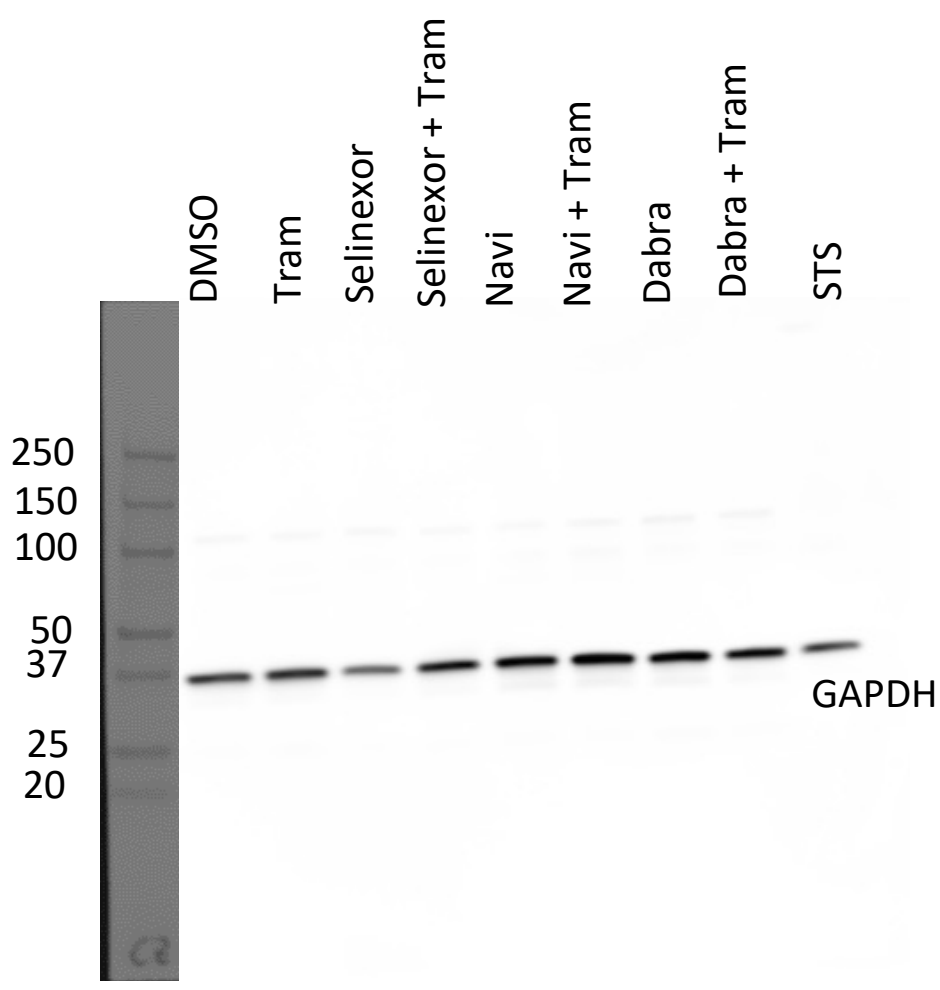

GAPDH; 1:50.000 37kDa in 2%Milk in TBS-T
o.n. 4°C
goat anti mouse 1:50.000; RT 1h

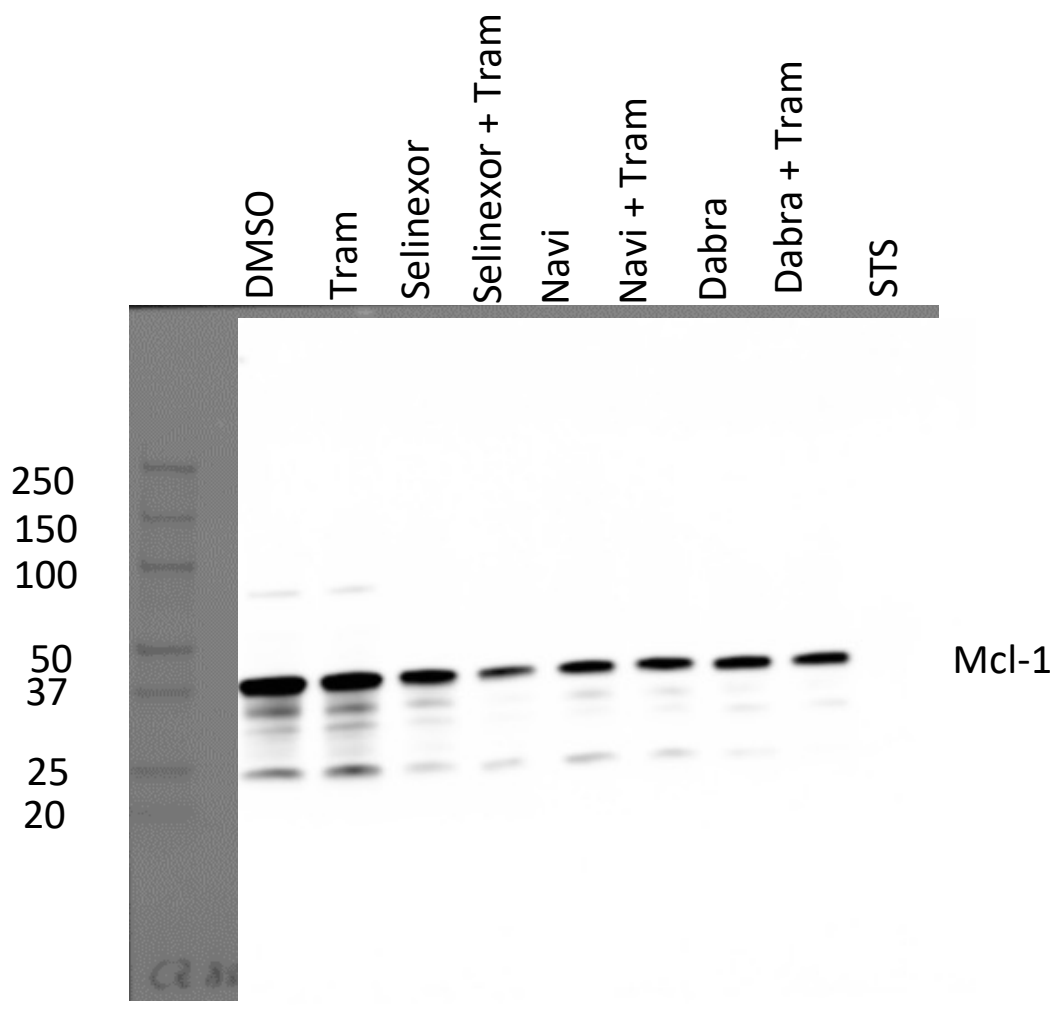

Mcl-1; 1:1000 40kDa in 2%Milk in TBS-T
o.n. 4°C
goat anti rabbit 1:50.000; RT 1h

BT314off uncropped – replicate 1,2

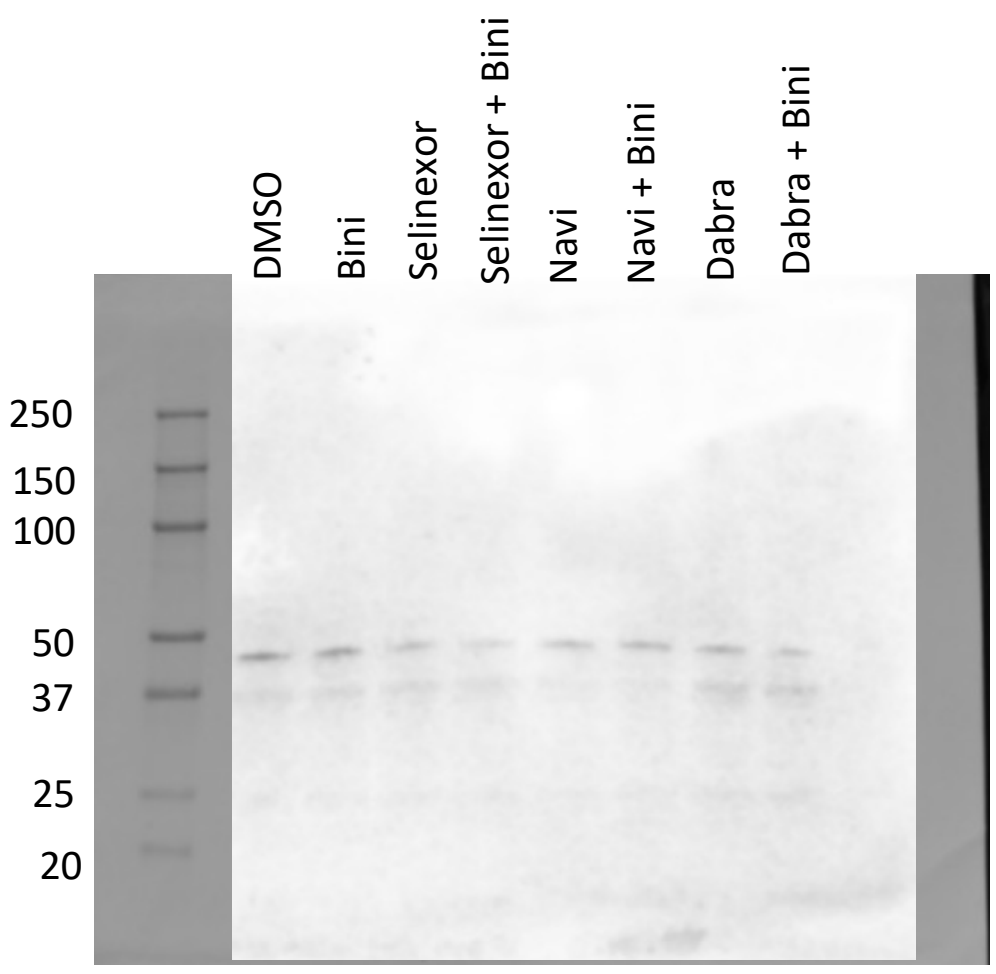

Mcl-1; 1:500 40kDa in 2%Milk in TBS-T
72h 4°C
goat anti rabbit 1:50.000; RT 1h

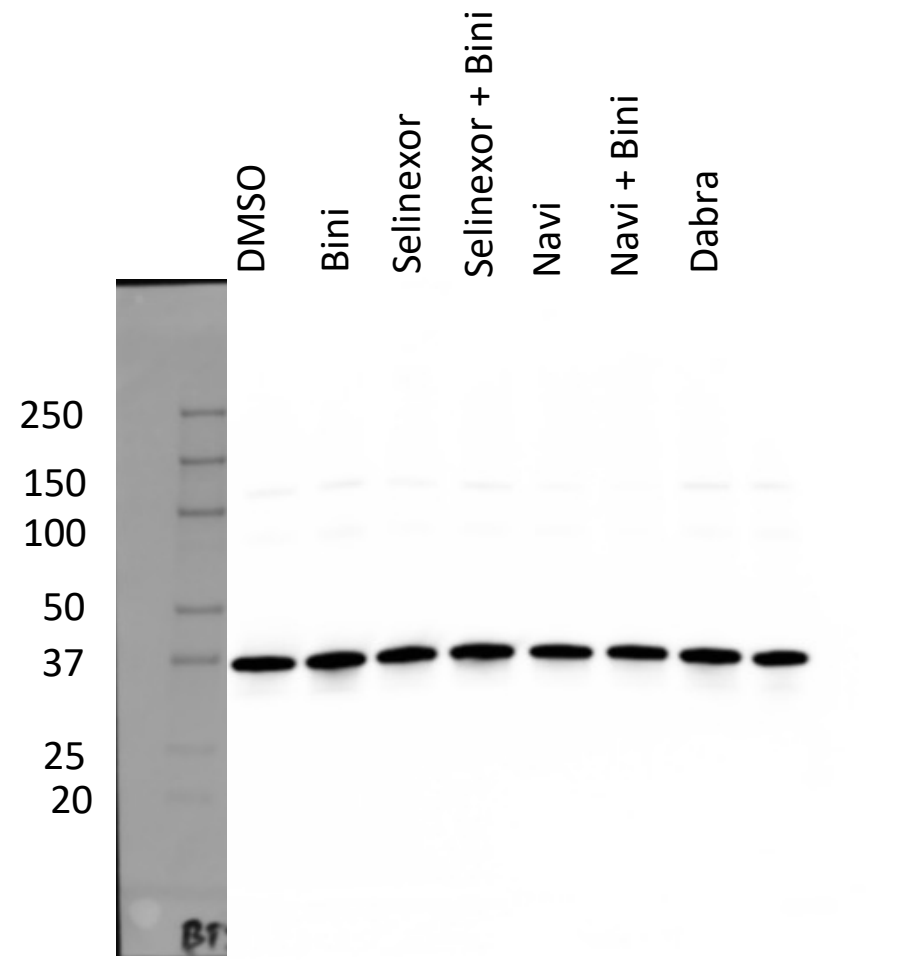

GAPDH; 1:50.000 37kDa in 2%Milk in TBS-T
o.n. 4°C
goat anti mouse 1:50.000; RT 1h

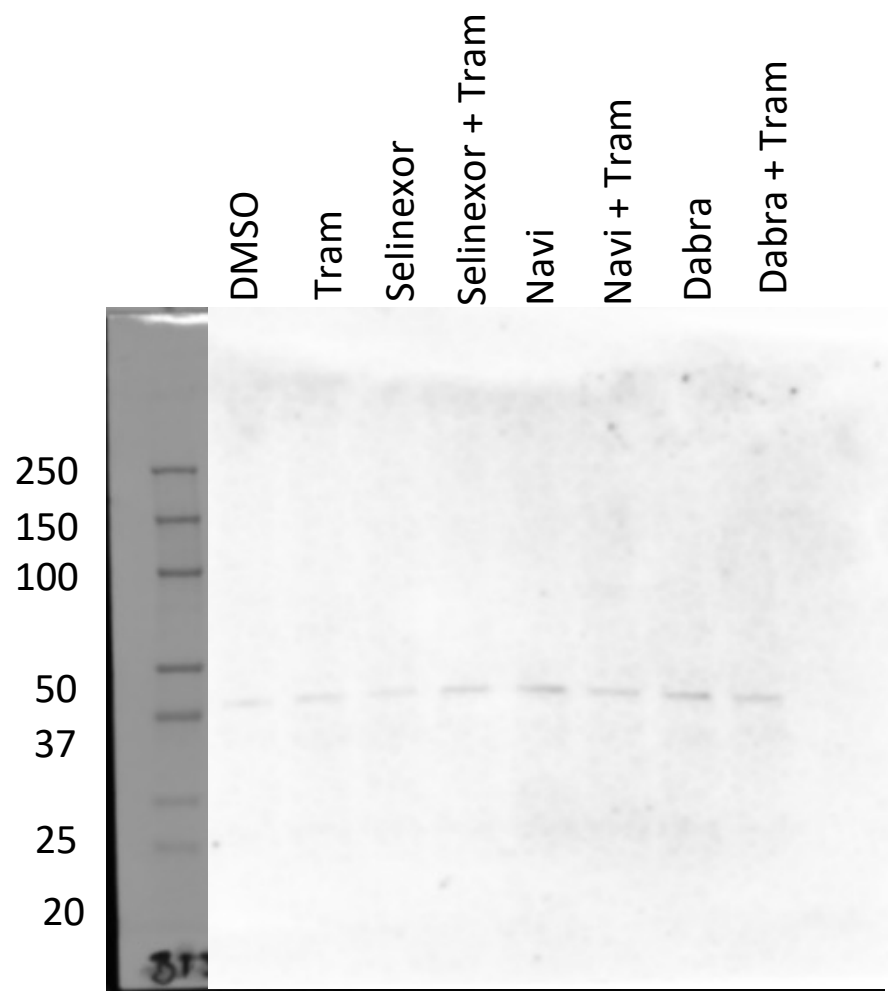

Mcl-1; 1:500 40kDa in 2%Milk in TBS-T
72h 4°C
goat anti rabbit 1:50.000; RT 1h

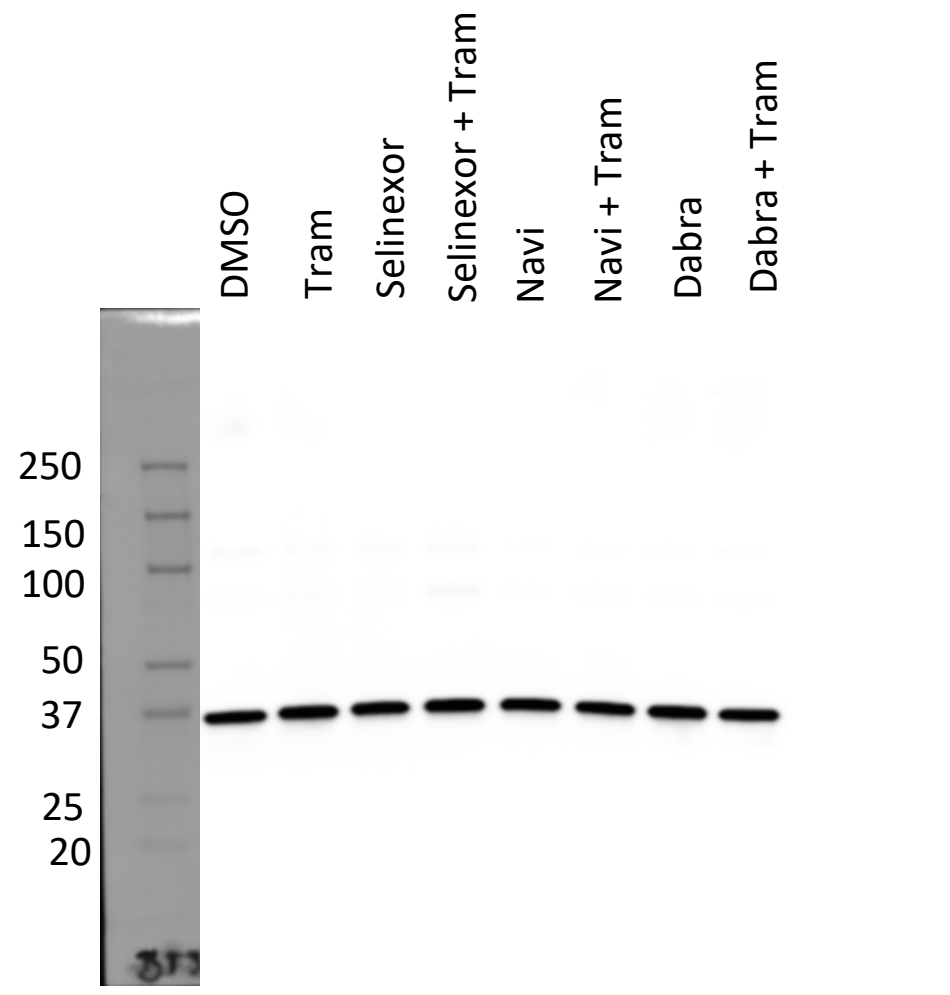

GAPDH; 1:50.000 37kDa in 2%Milk in TBS-T
o.n. 4°C
goat anti mouse 1:50.000; RT 1h

BT314off uncropped – replicate 1,2

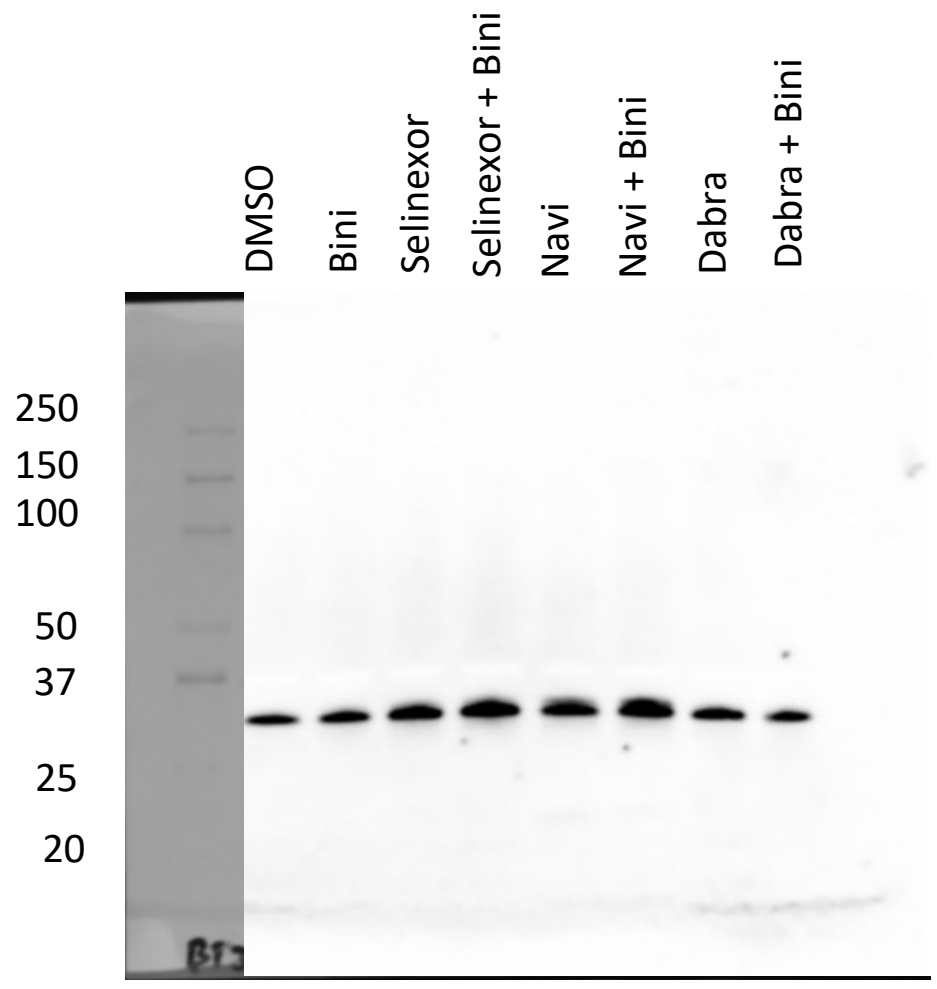

BclXL; 1:1000 30kDa in 5%BSA in TBS-T
24h 4°C
goat anti rabbit 1:20.000; RT 1h

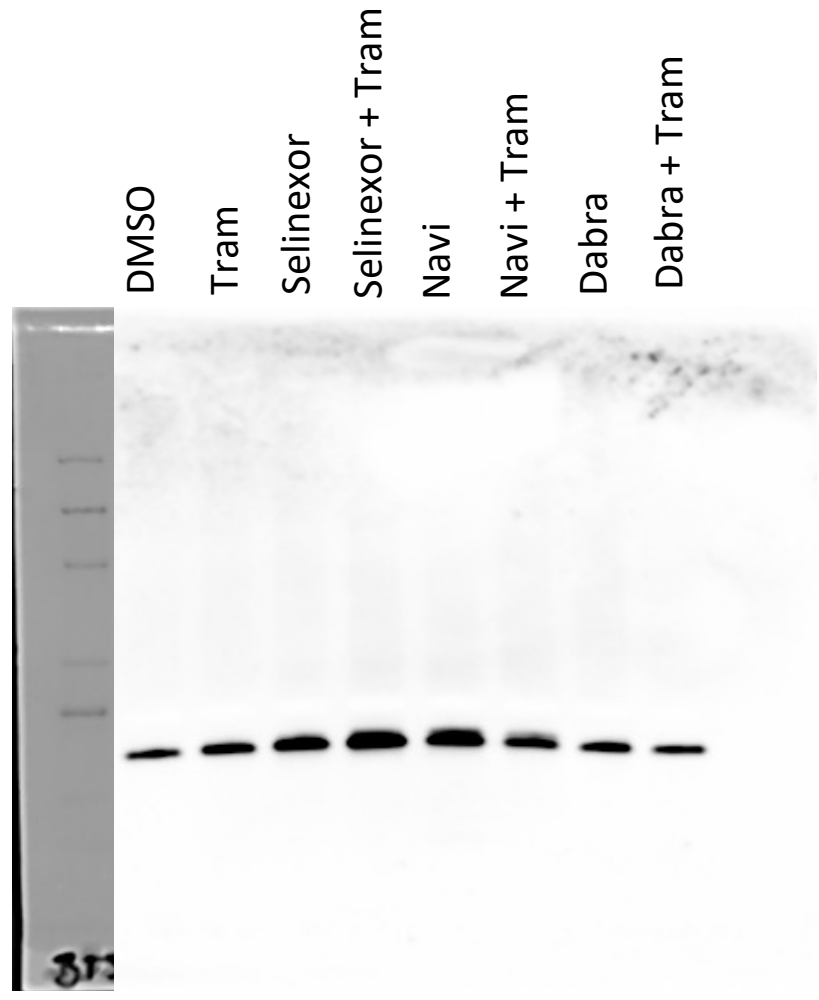

BclXL; 1:1000 30kDa in 5%BSA in TBS-T
24h 4°C
goat anti rabbit 1:20.000; RT 1h

BT314OFF
Bini 15ug protein
4-15 % TGX Gel BioRad
Fast blot Bio Rad 1A, 25V 60min

Stripping at 55°C in
ß-Mercaptho/Tris/SDS
Blocking 5%BSA in TBS-T

Navi 100 nM
Dabra 25 nM
Selinexor 100nM
Bini 58.6 nM
Trameti 1.74 nM
BT314OFF/Zeitpunkt: 48h
SDS Lysate
