# Supplementary material for: Cytotoxic activity of selinexor in BRAFV600E-driven pediatric low-grade glioma 3D cultures
Source: Acta Neuropathol Commun. 2026 Oct 22;14:220. doi: 10.1186/s40478-026-02461-1 (PMC13689195; doi:10.1186/s40478-026-02461-1)
Supplement: Supplementary file 2 [file 40478_2026_2461_MOESM3_ESM.pdf]

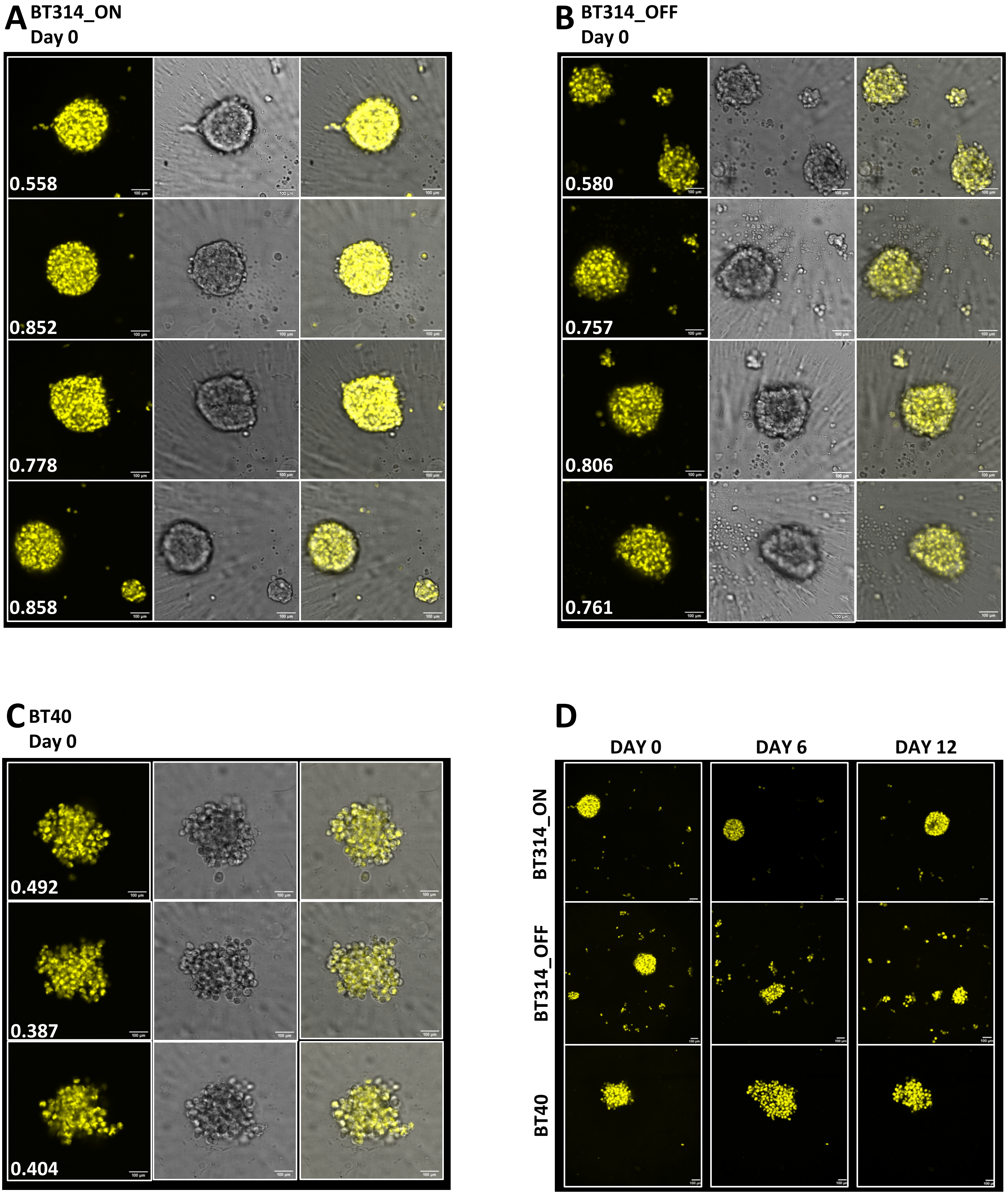

E

Model	Day 0	Day 3/4	Day 6/7	Day 12/14
**BT314_ON**	0.781 ± 0.077 (n=16)	0.802 ± 0.094 (n=16)	0.776 ± 0.136 (n=16)	0.771 ± 0.157 (n=16)
**BT314_OFF**	0.770 ± 0.084 (n=16)	0.632 ± 0.095 (n=16)	0.649 ± 0.157 (n=16)	0.690 ± 0.125 (n=16)
**BT40**	0.696 ± 0.200 (n=42)	0.563 ± 0.241 (n=42)	0.545 ± 0.226 (n=42)	0.530 ± 0.172 (n=42)

Suppl. Figure 1

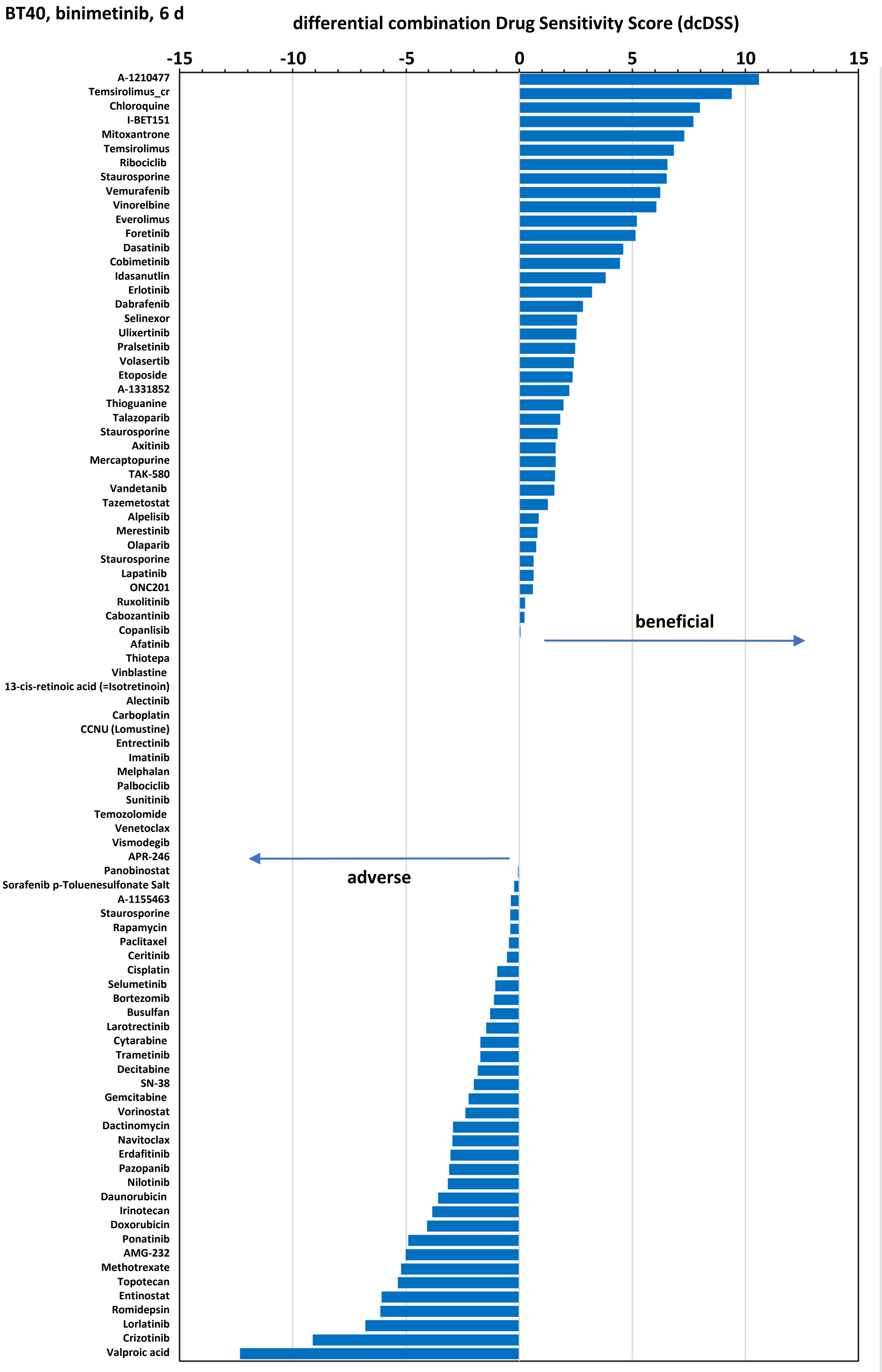

Suppl. Figure 2

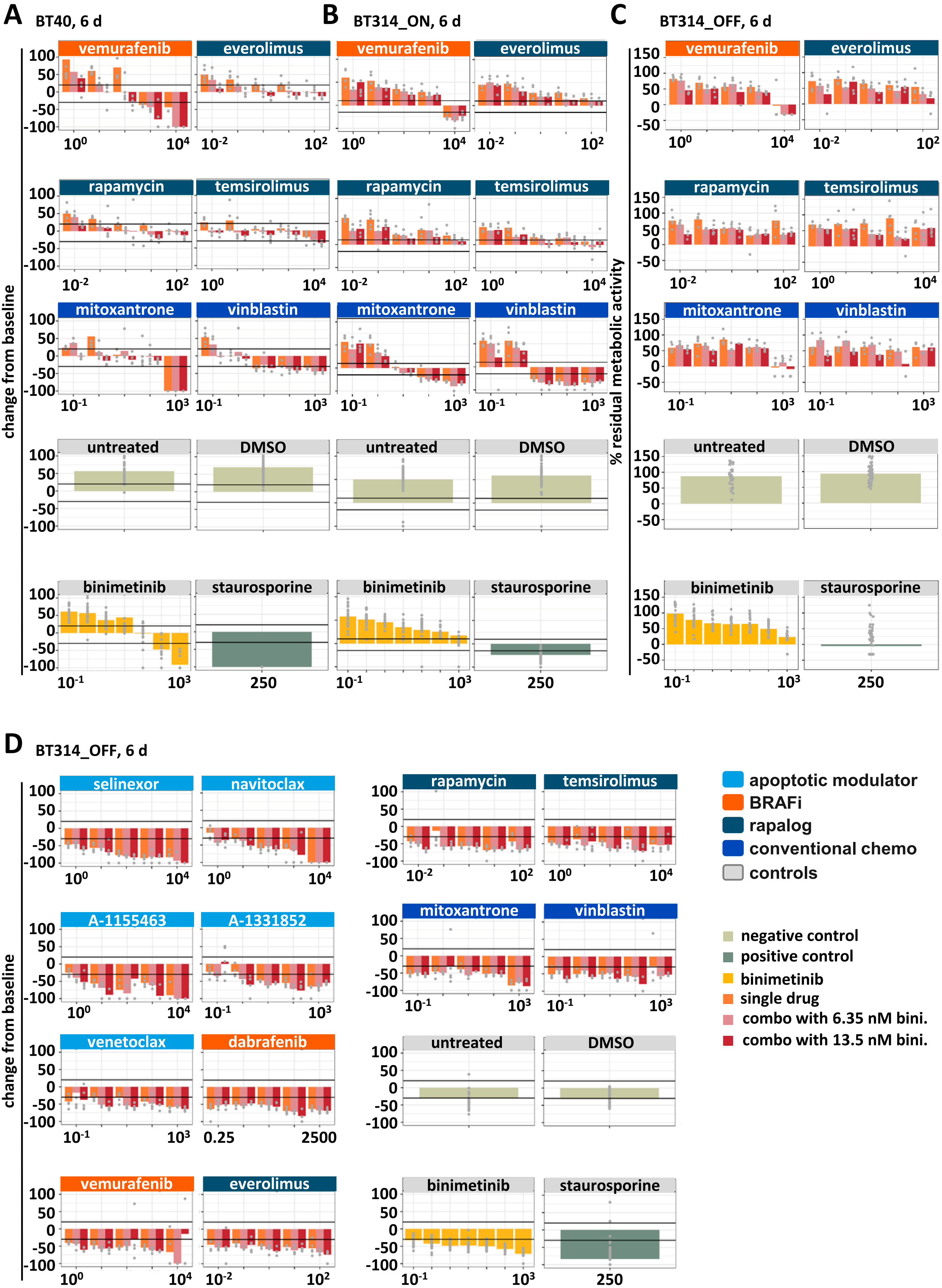

Suppl. Figure 3

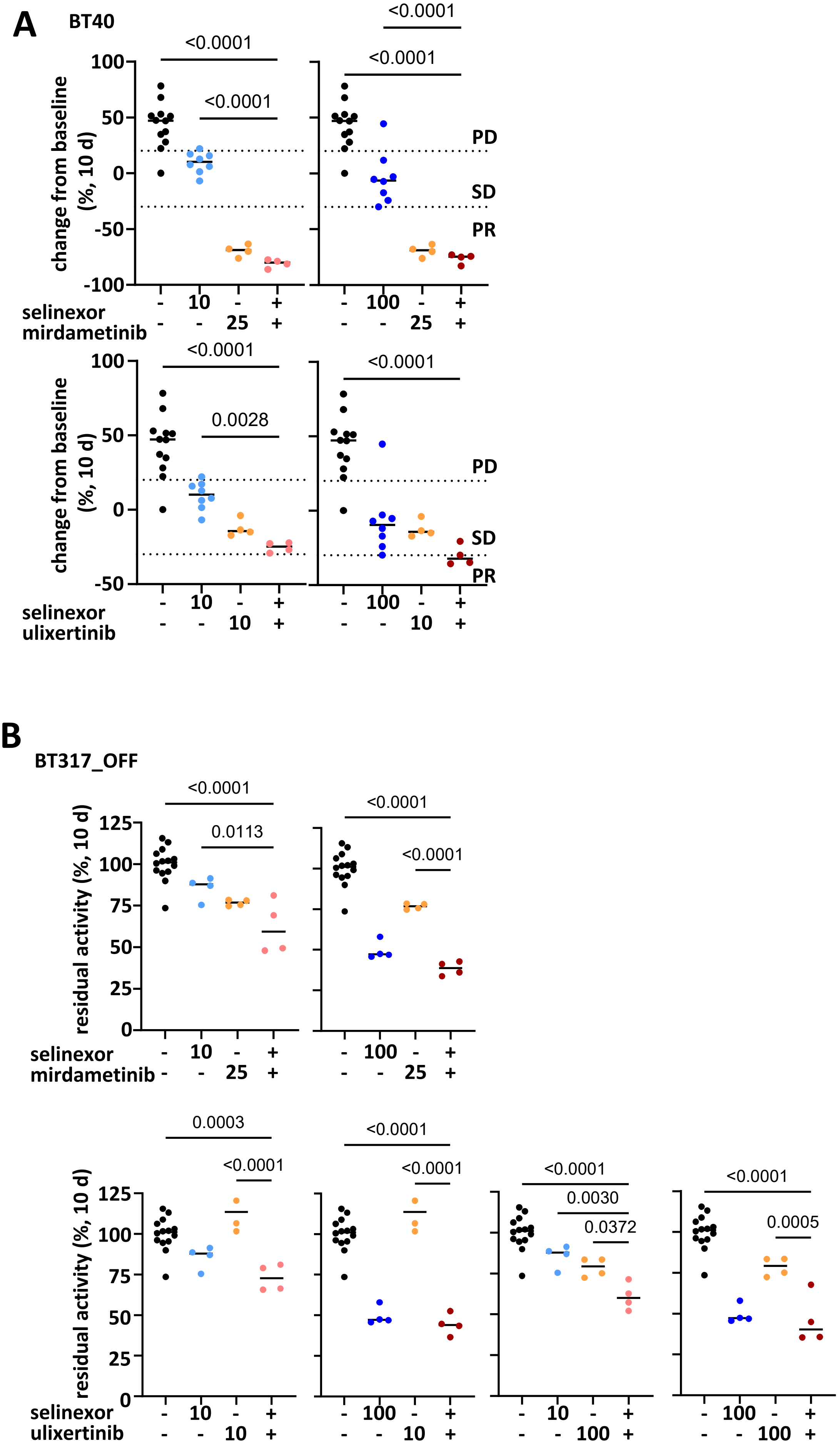

Suppl. Figure 4

Suppl. Figure 5

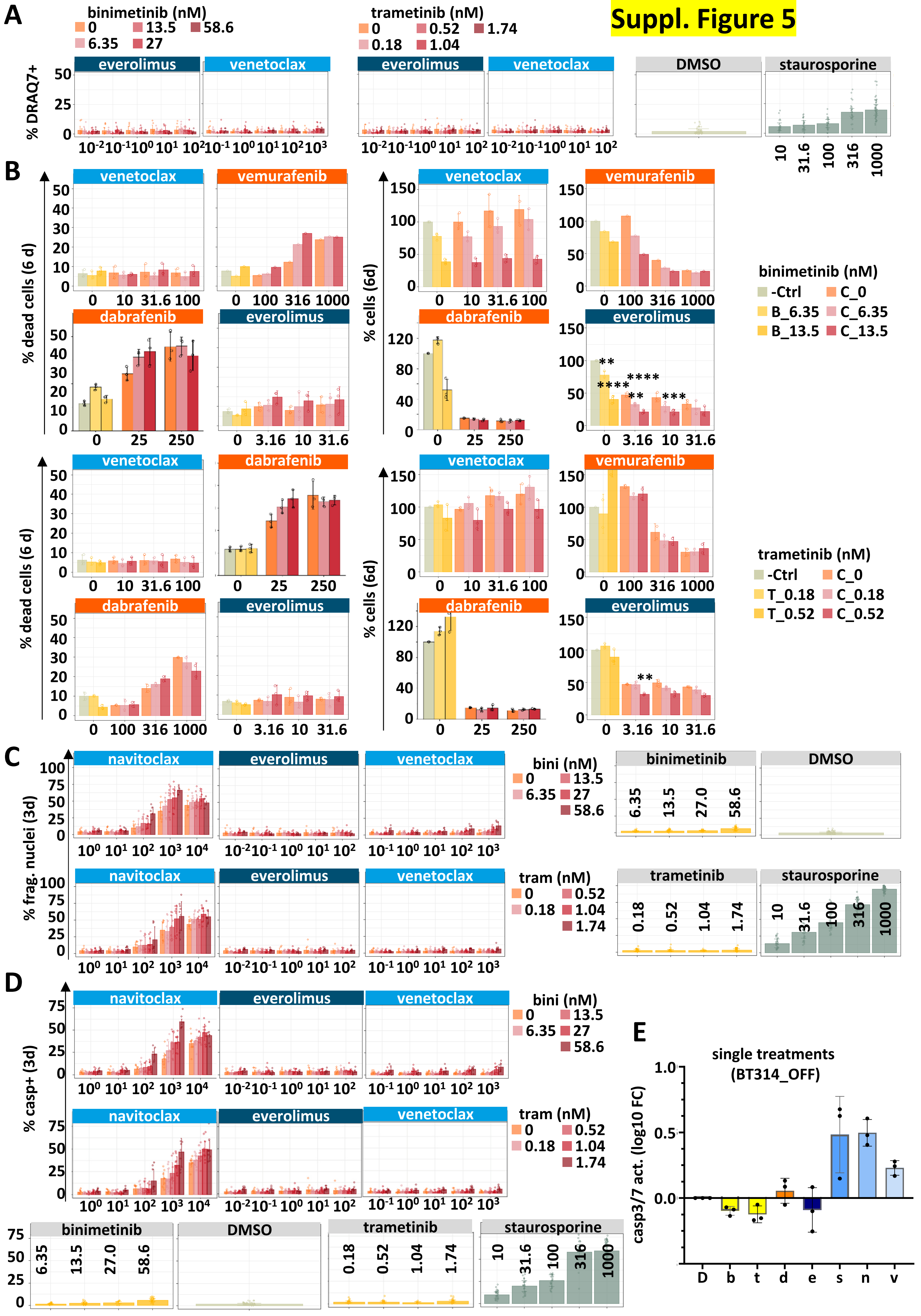

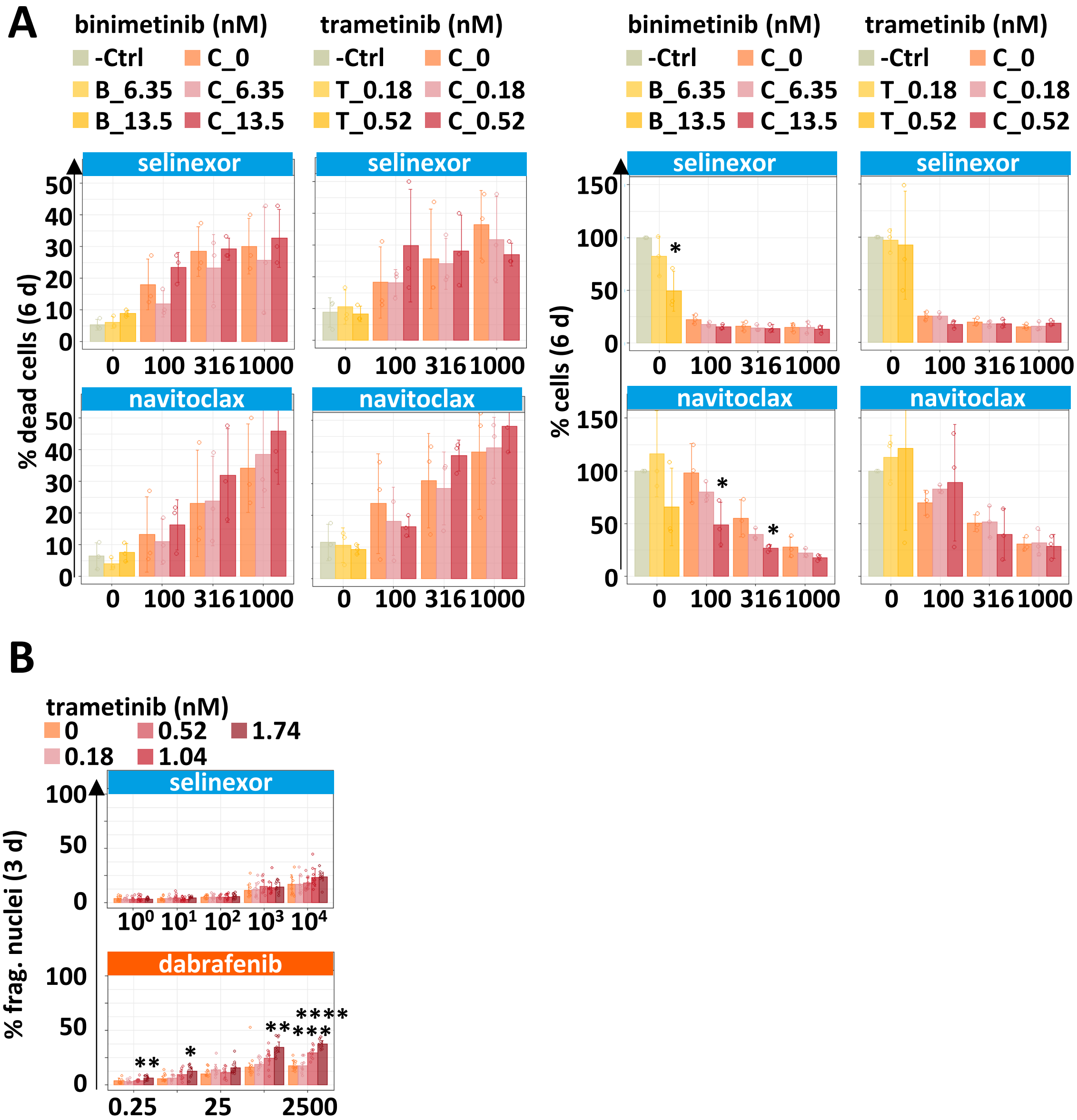

Suppl. Figure 6 – rev2

A

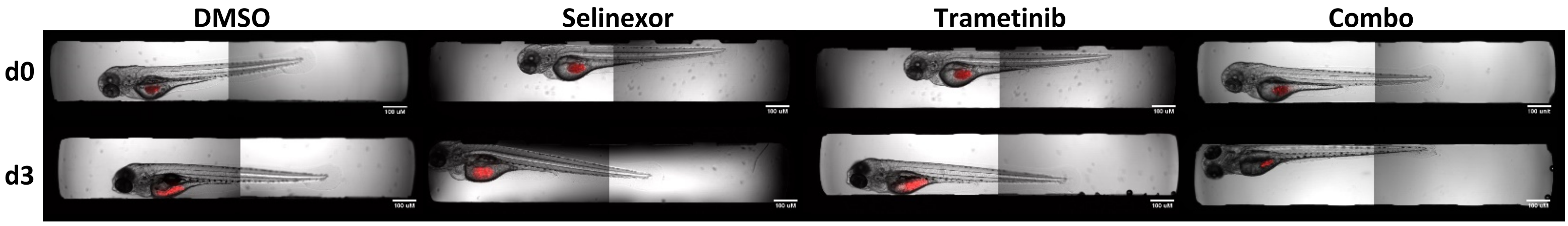

B

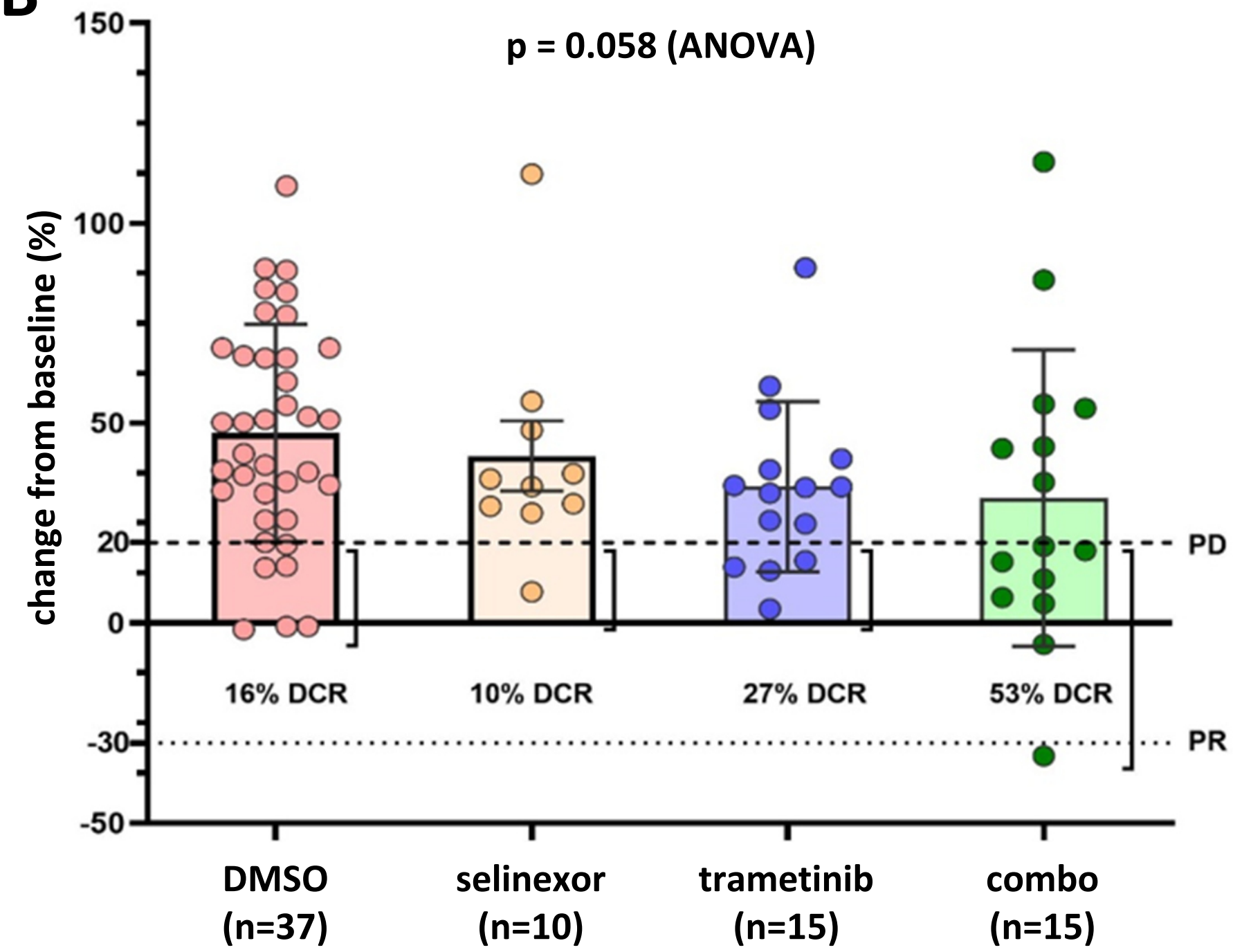

Suppl. Figure 7 –rev2

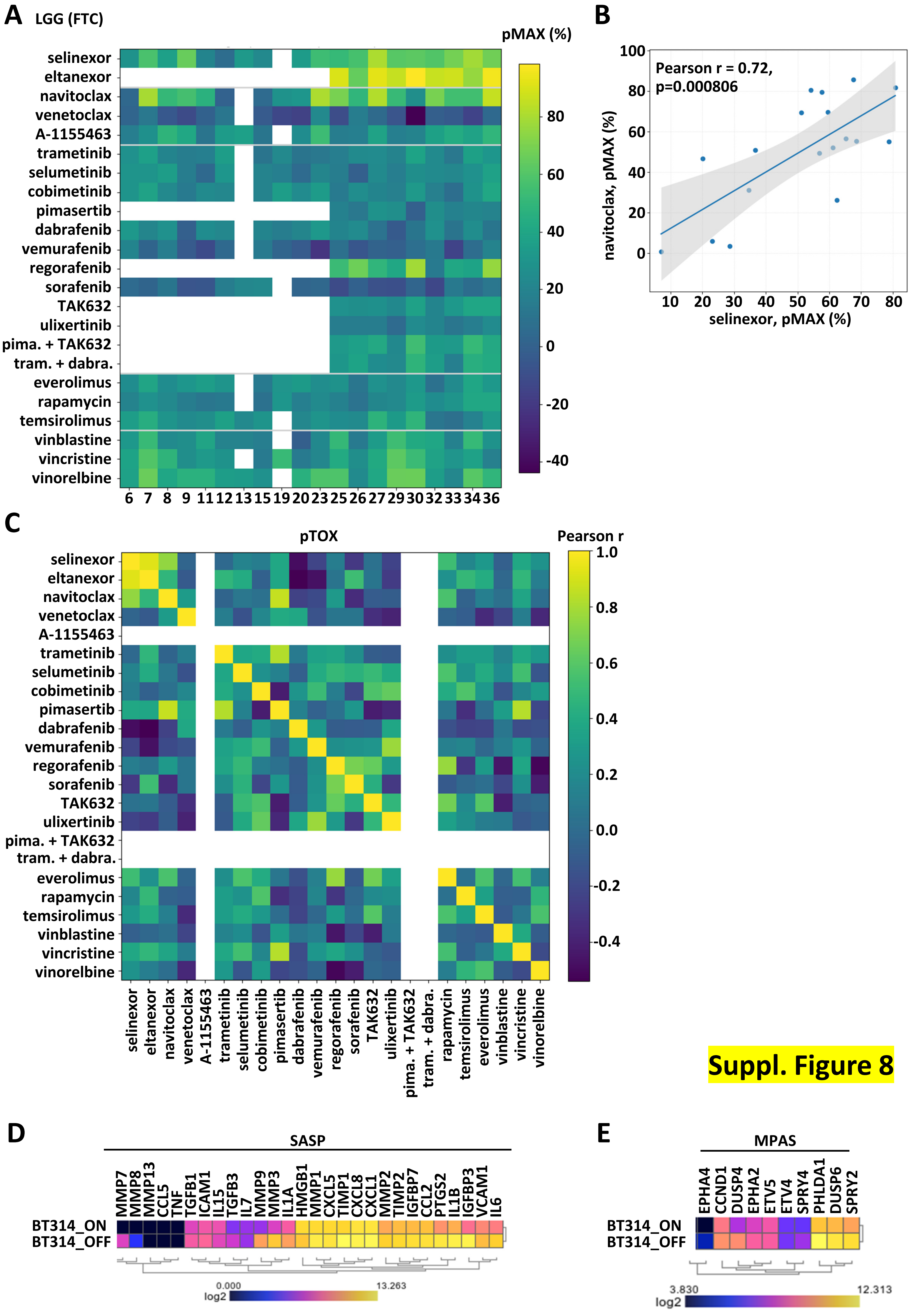

Suppl. Figure 8
