# Supplementary material for: Cytotoxic activity of selinexor in BRAFV600E-driven pediatric low-grade glioma 3D cultures
Source: Acta Neuropathol Commun. 2026 Oct 22;14:220. doi: 10.1186/s40478-026-02461-1 (PMC13689195; doi:10.1186/s40478-026-02461-1)
Supplement: Supplementary file 3 [file 40478_2026_2461_MOESM1_ESM.docx]

**Supplementary Table 1**

LOGGIC patient samples.

| **ID** | **histology** | **molecular diagnosis** | **alteration** |
| --- | --- | --- | --- |
| LOGGIC_DSP_006_TUE_DS1 | ganglioglioma (LGG WHO I) | methylation family low-grade glial/glioneuronal tumors | BRAF V600E |
| LOGGIC_DSP_007_TUE_DS1 | PA (LGG WHO I) | n.a. | n.d. |
| LOGGIC_DSP_008_TUE_DS1 | PA (LGG WHO I) | PA_INF | KIAA1549:BRAF Fusion |
| LOGGIC_DSP_009_TUE_DS1 | PA (preliminary diagnosis) | n.a. | n.d. |
| LOGGIC_DSP_011_TUE_DS1 | PA (preliminary diagnosis) | n.a. | n.d. |
| LOGGIC_DSP_010_HD_DS1 | PA (LGG WHO I) | PA_INF | KIAA1549:BRAF fusion |
| LOGGIC_DSP_012_TUE_DS1 | ganglioglioma (LGG WHO I) | Control tissue | BRAF V600E |
| LOGGIC_DSP_013_TUE_DS1 | PA (LGG WHO I) | PA_INF | KIAA1549:BRAF fusion |
| LOGGIC_DSP_015_HD_DS1 | PA (LGG WHO I) | PA_INF | KIAA1549:BRAF fusion |
| LOGGIC_DSP_019_HD_DS1 | PA (LGG WHO I) | PA_INF | KIAA1549:BRAF fusion |
| LOGGIC_DSP_020_TUE_DS1 | PA (LGG WHO I) | PA_MID | KIAA1549:BRAF fusion |
| LOGGIC_DSP_023_HD_DS1 | PA (LGG WHO I) | PA_INF | KIAA1549:BRAF fusion |
| LOGGIC_DSP_025_TUE_DS1 | PA (preliminary diagnosis) | n.a. | n.d. |
| LOGGIC_DSP_026_TUE_DS1 | PA (LGG WHO I) | PA_MID | KIAA1549:BRAF fusion |
| LOGGIC_DSP_027_TUE_DS1 | PA (LGG WHO I) | No match (best match PA_INF) | KIAA1549:BRAF fusion |
| LOGGIC_DSP_029_TUE_DS1 | PA (LGG WHO I), residual/recurrent | n.a. | n.d. |
| LOGGIC_DSP_030_GI_DS1 | astrocytoma (LGG WHO II) | n.a. | IDHmut |
| LOGGIC_DSP_032_TUE_DS1 | PA (LGG WHO I) | PA_MID | FGFR1 (p.N546K) |
| LOGGIC_DSP_033_TUE_DS1 | PA (LGG WHO I) | PA_INF | Indication from CNV for a KIAA1549::BRAF fusion |
| LOGGIC_DSP_034_TUE_DS1 | PA (LGG WHO I), with increased proliferative activity | n.a. | NF1 p.Q2513*  NF1 p.A2028P  TP53: Mutation  CDKN2A: no homozygous deletion |
| LOGGIC_DSP_036_TUE_DS1 | Low-grade diffuse neuroepithelial tumor | Low-grade diffuse neuroepithelial tumor | DNET |

n.d., not determined; n.a., not available; PA, pilocytic astrocytoma; PA_INF, infratentorial pilocytic astrocytoma; PA_MID, midline pilocytic astrocytoma; DNET, dysembryoplastic neuroepithelial tumor

**Supplementary Table 2**:

Extended version of the in-house drug library as described in Herter et al. (1) with tested concentrations (c1 – c5) [26]. All drugs were stored under nitrogen gas at room temperature.

| **Substance** | **Solvent** | **c1 [nM]** | **c2 [nM]** | **c3**  **[nM]** | **c4**  **[nM]** | **c5**  **[nM]** | **Company** | **Cat. No.** |
| --- | --- | --- | --- | --- | --- | --- | --- | --- |
| A-1155463 | DMSO | 10000 | 1000 | 100 | 10 | 1 | ChemieTek | CT-A115 |
| A-1210477 | DMSO | 50000 | 5000 | 500 | 50 | 5 | Active Biochem | A-9036 |
| A-1331852 | DMSO | 1000 | 100 | 10 | 1 | 0.1 | ChemieTek | CT-A133 |
| Afatinib | DMSO | 1000 | 100 | 10 | 1 | 0.1 | Selleck | S1011 |
| Alectinib | DMSO | 1000 | 100 | 10 | 1 | 0.1 | ChemieTek | CT-CH542 |
| Alpelisib | DMSO | 10000 | 1000 | 100 | 10 | 1 | Medchem Express | HY-15244 |
| AMG-232 | DMSO | 10000 | 1000 | 100 | 10 | 1 | ChemieTek | CT-AMG232 |
| APR-246 | DMSO | 10000 | 1000 | 100 | 10 | 1 | Tocris Biosciences | 3710 |
| APR-246  (2nd range) | DMSO | 100000 | 10000 | 1000 | 100 | 10 | Tocris Biosciences | 3710 |
| Axitinib | DMSO | 10000 | 1000 | 100 | 10 | 1 | LC Laboratories | A-1107 |
| Bortezomib | DMSO | 1000 | 100 | 10 | 1 | 0.1 | ChemieTek | CT-BZ001 |
| Busulfan | DMSO | 10000 | 1000 | 100 | 10 | 1 | Sigma-Aldrich | B2635 |
| Cabozantinib | DMSO | 1000 | 100 | 10 | 1 | 0.1 | ChemieTek | CT-XL184 |
| Carboplatin | AQ | 10000 | 1000 | 100 | 10 | 1 | Hospital pharmacy Heidelberg | Cay13112-25 |
| CCNU (Lomus-  tine) | DMSO | 10000 | 1000 | 100 | 10 | 1 | Medchem Express | HY-13669 |
| Ceritinib | DMSO | 2500 | 250 | 25 | 2.5 | 0.25 | Selleck | S7083 |
| Chloroquine | AQ | 100000 | 10000 | 1000 | 100 | 10 | Sigma-Aldrich | C6628 |
| Cisplatin | AQ | 100000 | 10000 | 1000 | 100 | 10 | Hospital pharmacy Heidelberg | - |
| Cobimetinib | DMSO | 1000 | 100 | 10 | 1 | 0.1 | Medchem Express | HY-13064 |
| Copanlisib | AQ | 1000 | 100 | 10 | 1 | 0.1 | Medchem Express | HY-15346 |
| Crizotinib | DMSO | 10000 | 1000 | 100 | 10 | 1 | Selleck | S1068-5 |
| Cytarabine | DMSO | 10000 | 1000 | 100 | 10 | 1 | Medchem Express | HY-13605 |
| Dabrafenib | DMSO | 2500 | 250 | 25 | 2.5 | 0.25 | ChemieTek | CT-DABR |
| Dactinomycin | DMSO | 1000 | 100 | 10 | 1 | 0.1 | Medchem  Express | HY-17559 |
| Dasatinib | DMSO | 1000 | 100 | 10 | 1 | 0.1 | LC Laboratories | D-3307 |
| Daunorubicin | DMSO | 1000 | 100 | 10 | 1 | 0.1 | Medchem Express | HY-13062 |
| Decitabine | DMSO | 10000 | 1000 | 100 | 10 | 1 | Selleck | S1200 |
| Doxorubicin | DMSO | 1000 | 100 | 10 | 1 | 0.1 | Sigma-Aldrich | D1515 |
| Entinostat | DMSO | 10000 | 1000 | 100 | 10 | 1 | ChemieTek | CT-MS275 |
| Entrectinib | DMSO | 1000 | 100 | 10 | 1 | 0.1 | Medchem Express | HY-12678 |
| Erdafitinib | DMSO | 10000 | 1000 | 100 | 10 | 1 | Medchem Express | HY-18708 |
| Erlotinib | DMSO | 10000 | 1000 | 100 | 10 | 1 | Medchem Express | HY-50896 |
| Etoposide | DMSO | 10000 | 1000 | 100 | 10 | 1 | Medchem Express | HY-13629 |
| Everolimus | DMSO | 100 | 10 | 1 | 0.1 | 0.01 | LC Laboratories | E-4040 |
| Foretinib | DMSO | 1000 | 100 | 10 | 1 | 0.1 | Selleck | S1111 |
| Gemcitabine | DMSO | 1000 | 100 | 10 | 1 | 0.1 | Medchem Express | 9505881-4 |
| I-BET151 | DMSO | 10000 | 1000 | 100 | 10 | 1 | ChemieTek | CTBET151 |
| Idasanutlin | DMSO | 10000 | 1000 | 100 | 10 | 1 | Medchem Express | HY-15676 |
| Imatinib | DMSO | 10000 | 1000 | 100 | 10 | 1 | Medchem Express | HY-50946 |
| Irinotecan | DMSO | 10000 | 1000 | 100 | 10 | 1 | LC Laboratories | I-4122 |
| Isotretionin | DMSO | 10000 | 1000 | 100 | 10 | 1 | Hölzel | TMO-T1611 |
| Lapatinib | DMSO | 1000 | 100 | 10 | 1 | 0.1 | LC Laboratories | L-4804 |
| Larotrectinib | DMSO | 1000 | 100 | 10 | 1 | 0.1 | Medchem Express | HY-12866 |
| Lorlatinib | DMSO | 10000 | 1000 | 100 | 10 | 1 | Medchem Express | HY-12215 |
| Melphalan | AQ | 10000 | 1000 | 100 | 10 | 1 | Sigma-Aldrich | M2011 |
| Mercaptopurine | DMSO | 10000 | 1000 | 100 | 10 | 1 | Medchem Express | HY-13677 |
| Merestinib | DMSO | 1000 | 100 | 10 | 1 | 0.1 | Medchem Express | HY15514A |
| Methothrexate | DMSO | 5000 | 500 | 50 | 5 | 0.5 | Selleck | S1210 |
| Mitoxantrone | DMSO | 1000 | 100 | 10 | 1 | 0.1 | Medchem Express | HY13502A |
| Navitoclax | DMSO | 10000 | 1000 | 100 | 10 | 1 | Medchem Express | HY-10087 |
| Nilotinib | DMSO | 10000 | 1000 | 100 | 10 | 1 | LC Laboratories | N-8207 |
| Olaparib | DMSO | 10000 | 1000 | 100 | 10 | 1 | LC Laboratories | O-9201 |
| ONC201 | DMSO | 10000 | 1000 | 100 | 10 | 1 | Selleck | S7963 |
| Paclitaxel | DMSO | 1000 | 100 | 10 | 1 | 0.1 | Medchem Express | HY-B0015 |
| Palbociclib | AQ | 10000 | 1000 | 100 | 10 | 1 | Selleck | S111610 |
| Panobinostat | DMSO | 1000 | 100 | 10 | 1 | 0.1 | LC Laboratories | P-3703 |
| Pazopanib | DMSO | 10000 | 1000 | 100 | 10 | 1 | LC Laboratories | P-6706 |
| Ponatinib | DMSO | 1000 | 100 | 10 | 1 | 0.1 | Selleck | S1490 |
| Pralsetinib | DMSO | 10000 | 1000 | 100 | 10 | 1 | BIOZOL | TMOTQ0277 |
| Rapamycin | DMSO | 100 | 10 | 1 | 0.1 | 0.01 | LC Laboratories | R-5000 |
| Ribociclib | DMSO | 10000 | 1000 | 100 | 10 | 1 | Selleck | S7440 |
| Romidepsin | DMSO | 1000 | 100 | 10 | 1 | 0.1 | Medchem Express | HY-15149 |
| Ruxolitinib | DMSO | 10000 | 1000 | 100 | 10 | 1 | ChemieTek | CT-INCB-2 |
| Selinexor | DMSO | 10000 | 1000 | 100 | 10 | 1 | Selleck | S7252 |
| Selumetinib | DMSO | 10000 | 1000 | 100 | 10 | 1 | Medchem Express | HY-50706 |
| SN-38 | DMSO | 100 | 10 | 1 | 0.1 | 0.01 | Medchem Express | HY-13704 |
| Sorafenib p-Toluenesulfonate Salt | DMSO | 1000 | 100 | 10 | 1 | 0.1 | LC Laboratories | S-8502 |
| Staurosporine | DMSO | 1000 | 100 | 10 | 1 | 0.1 | Selleck | S1421 |
| Sunitinib | DMSO | 1000 | 100 | 10 | 1 | 0.1 | LC Laboratories | S-8803 |
| TAK-580 | DMSO | 10000 | 1000 | 100 | 10 | 1 | Medchem Express | HY-15246 |
| Talazoparib | DMSO | 1000 | 100 | 10 | 1 | 0.1 | Medchem Express | HY-16106 |
| Tazemetostat | DMSO | 10000 | 1000 | 100 | 10 | 1 | ChemieTek | CTEPZ438 |
| Temozolomide | DMSO | 100000 | 10000 | 1000 | 100 | 10 | Selleck | S1237 |
| Temsirolimus | DMSO | 100 | 10 | 1 | 0.1 | 0.01 | LC Laboratories | T-8040 |
| Temsirolimus  (2nd range) | DMSO | 10000 | 1000 | 100 | 10 | 1 | LC Laboratories | T-8040 |
| Thioguanine | DMSO | 10000 | 1000 | 100 | 10 | 1 | Medchem Express | HY-13765 |
| Thiotepa | DMSO | 50000 | 5000 | 500 | 50 | 5 | Sigma-Aldrich | T6069 |
| Topotecan | DMSO | 10000 | 1000 | 100 | 10 | 1 | Medchem Express | HY13768A |
| Trametinib | DMSO | 2500 | 250 | 25 | 2.5 | 0.25 | ChemieTek | CTGSK112 |
| Ulixertinib | DMSO | 10000 | 1000 | 100 | 10 | 1 | Selleck | S7854 |
| Valproic acid | AQ | 1x10^6^ | 100000 | 10000 | 1000 | 100 | Sigma-Aldrich | P4543 |
| Vandetanib | DMSO | 1000 | 100 | 10 | 1 | 0.1 | LC Laboratories | V-9402 |
| Vemurafenib | DMSO | 10000 | 1000 | 100 | 10 | 1 | ChemieTek | CTP4032-2 |
| Venetoclax | DMSO | 1000 | 100 | 10 | 1 | 0.1 | ChemieTek | CTA199-2 |
| Vinblastine | DMSO | 1000 | 100 | 10 | 1 | 0.1 | Medchem Express | HY-13780 |
| Vincristine | DMSO | 1000 | 100 | 10 | 1 | 0.1 | Selleck | S1241 |
| Vinorelbine | DMSO | 10000 | 1000 | 100 | 10 | 1 | Selleck | S4269 |
| Vismodegib | DMSO | 10000 | 1000 | 100 | 10 | 1 | LC Laboratories | V-4050 |
| Volasertib | DMSO | 1000 | 100 | 10 | 1 | 0.1 | ChemieTek | CT-BI6727 |
| Vorinostat | DMSO | 10000 | 1000 | 100 | 10 | 1 | LC Laboratories | V-8477 |

1.	Herter S, Emperador M, Smyrilli K, Kocher D, Celikyurekli S, Zeiser C, et al. High content-imaging drug synergy screening identifies specific senescence-related vulnerabilities of mesenchymal neuroblastomas. Cell death & disease. 2025;16(1):644.

**Supplementary Table 3**

Additional drugs and substances used for in vitro assays.

| **Substance** | **Solvent** | **Company** | **Cat. No.** | **Storage** |
| --- | --- | --- | --- | --- |
| Benzethonium chloride | DMSO | Selleck | S4162 | under nitrogen gas at RT |
| Binimetinib | DMSO | Selleck | S7007 | under nitrogen gas at RT |
| Staurosporine | DMSO | Selleck | S1421 | under nitrogen gas at RT |

**Supplementary Table 4**

Fluorescence marker conditions for high content microscopy analysis.

| **Marker** | **Stained cell structure** | **Function** | **Supplier** | **Cat. No.** | **Conc.** | **Channel** |
| --- | --- | --- | --- | --- | --- | --- |
| Tetramethylrhodamine ethyl ester (TMRE) | active mitochondria | viable cells | Abcam | ab113852 | 0.05 µM | Cy3 |
| Hoechst 33342 | nucleic acid | whole nuclei (total cell number) and fragmented nuclei (dead cells) | ThermoFisher Scientific | H3570 | 0.5 µg/ml | DAPI |
| CellEvent Caspase-3/7 Green | nucleic acid | fluorescence indicates active caspases | ThermoFisher Scientific | C10423 | 1 µM | FITC |
| DRAQ7 | nucleic acid | dead cells with damaged membranes | ThermoFisher  Scientific | D15106 | 0.1 µM | Cy5 |

**Supplementary Table 5**

CellProfiler pipeline used to identify the area of the spheroids in the Bright-field images and in the maximal intensity projection (MIP) of the fluorescent images. All non-mentioned settings were maintained in default mode.

| **Order** | **Modul** | **Setting** |
| --- | --- | --- |
| 1 | Crop | Select input image: *Brightfield*  Select the cropping shape: *Rectangle*  Select the cropping method: *Coordinates* |
| 2 | Smooth | Select input image: *Image generated by step 1*  Select smoothing method: *Median filter*  Calculate artefact diameter automatically: *Yes* |
| 3 | Image Math  (only Brightfield) | Operation: *Invert*  Select the first image: *Image generated by step 2* |
| 4 | Smooth  (only Brightfield) | Select input image: *Image generated by step 3*  Select smoothing method: *Median filter*  Calculate artefact diameter automatically: Yes |
| 5 | IdentifyPrimaryObjects  (only Brightfield) | Select input image: *Image generated by step 4*  Typical diameter of the object, in pixel units: *100-400*  Discard object touching the border of the image: *No*  Threshold strategy: *Global*  Thresholding method: *Otsu*  Two-class or three-class thresholding? *Three classes*  Assign pixels in the middle intensity class to the foreground or the background? *Background*  Lower and upper bounds on threshold: *0.02-1.0*  Method to distinguish clumped objects: *Shape*  Method to draw dividing lines between clumped objects: *Shape* |
| 6 | IdentifyPrimaryObjects  (only MIP) | Select input image: *Image generated by step 2*  Typical diameter of the object, in pixel units: *100 - 400*  Discard object touching the border of the image: *No*  Threshold strategy: *Global*  Thresholding method: *Otsu*  Lower and upper bounds on threshold: *0.003 – 1.0*  Method to distinguish clumped objects: *Shape*  Method to draw dividing lines between clumped objects: *Shape* |
| 7 | MeasureObjectSizeShape | Select object sets to measure: *Objects generated by step 5 or 6* |
| 8 | OverlayOutlines | Select image on which to display outlines: *Brightfield/MIP*  Select objects to display: *Objects generated by step 5/6* |
| 9 | RescaleIntensity | Select input image: *Images generated by step 8* |
| 10 | SaveImages |  |
| 11 | ExporttoDatabase |  |

**Supplementary Table 6**

CellProfiler pipeline and Cell Profiler Analyst settings used to identify Caspase positive cells, DRAQ7 positive cells and fragmented nuclei. All non-mentioned settings were maintained in default mode.

| **Order** | **Modul** | **Setting** |
| --- | --- | --- |
| 1 | Smooth | Select input image: *Hoechst*  Select smoothing method: Median filter  Calculate artefact diameter automatically: Yes |
| 2 | IdentifyPrimaryObjects  (nuclei) | Select input image: *Image generated by step 1*  Typical diameter of the object, in pixel units: 20 - 100  Thresholding method: Global Minimum Cross-Entropy  Threshold correction factor: 1.3  Lower and upper bounds on threshold: 0.005 – 1.0  Method to distinguish clumped objects: Intensity |
| 3 | MeasureObjectIntensity | Select images to measure: *Hoechst, Caspase, DRAQ7*  Select objects to measure: *Objects identified in step 2* |
| 4 | MeasureObjectIntensity  Distribution | Select images to measure: *Hoechst, Caspase, DRAQ7*  Select objects to measure: *Objects identified in step 2* |
| 5 | MeasureObjectSizeShape | Select objects to measure: *Objects identified in step 2* |
| 6 | MeasureGranularity | Select images to measure: *Hoechst, Caspase, DRAQ7*  Measure within objects? Yes  Select objects to measure: *Objects identified in step 2* |
| 7 | ExporttoDatabase | Create a CellProfiler Analyst properties file? Yes  Which objects should be used for locations? *Objects identified in step 2* |
| 9 | CellProfiler Analyst | Open: *CellProfiler Analyst properties file generated by step 8* |
| 10 | Generating Classifier  Training set | *Objects identified in step 2 r*andomly fetched from the whole data set; manually annotated into two classes, respectively: Caspase positive and negative nuclei, DRAQ7 positive and negative nuclei or fragmented and healthy nuclei |
| 11 | Training Classifier | Use: Random Forest Classifier |
| 12 | Classifier | Apply training sets to the whole data set; Export Hit Table (grouped by Image) |
