# Supplementary material for: Cytotoxic activity of selinexor in BRAFV600E-driven pediatric low-grade glioma 3D cultures
Source: Acta Neuropathol Commun. 2026 Oct 22;14:220. doi: 10.1186/s40478-026-02461-1 (PMC13689195; doi:10.1186/s40478-026-02461-1)
Supplement: Supplementary file 4 [file 40478_2026_2461_MOESM5_ESM.docx]

**Supplementary Figure Legends**

**Supplementary Figure 1.** **Morphological characterization and long-term stability of pLGG 3D cultures.** Representative fluorescence images of BT314_ON, BT314_OFF, and BT40 3D cultures are shown together with the corresponding circularity values determined from fluorescence images. Quantitative analysis demonstrated compact spheroid morphology for BT314_ON and BT314_OFF cultures, whereas BT40 cultures displayed a more irregular morphology. Circularity was determined at multiple time points during the assay period. Data in the table are presented as mean circularity ± SD. The number of spheroids/aggregates analyzed per condition is indicated in the table. Scale bars, 100 μm.

**Supplementary Figure 2.** Median differential combination drug sensitivity scores (dcDSS) for all 87 library drugs combined with binimetinib, calculated using the iTReX application based on metabolic activity (CellTiter-Glo). Positive dcDSS values indicate beneficial combination effects, while negative values indicate adverse combination effects. dcDSS: differential combination drug sensitivity score.

**Suppl. Figure 3** Image-based analysis of drug responses in senescent BT314_OFF spheroids after 6-day treatment with single agents or in combination with 6.35 nM (IC_25_) or 13.5 nM (IC_50_) binimetinib. Values represent the relative change in TMRE-stained spheroid area compared with baseline (day 0), derived from the maximal intensity projection (MIP) images. Each grey dot represents a technical replicate (n = 5) with bars indicating the mean. Horizontal lines denote response thresholds based on RECIST-like criteria. All concentrations are given in nM; TMRE, tetramethylrhodamine ethyl ester; MIP, maximum intensity projection; RECIST, Response Evaluation Criteria in Solid Tumors.

**Suppl. Figure 4. Effect of selinexor and MAPK pathway inhibition on 3D culture growth in BT40 and BT314_OFF models.** **A** Change from baseline at day 10 following treatment with the indicated drugs and combinations. Individual data points represent replicates. Negative values indicate 3D culture size regression relative to baseline, whereas positive values indicate continued growth. Horizontal lines denote response thresholds based on RECIST-like criteria. **B** Residual metabolic activity was determined by CellTiter-Glo assay after 10 days of treatment with the indicated drugs and combinations. Data are shown for BT317_OFF spheroids. Values are presented as residual metabolic activity. Each dot represents one replicate.

**Suppl. Figure 5. A** Percentage of DRAQ7-positive cells (late-stage cell death) relative to total cell number. BT40 cells were treated as indicated for 72h. Data represents the mean ± SD of three independent biological replicates with small dots indicating individual wells. All concentrations indicated on the x-axis are expressed in nM. **B** Left: Percentage of dead cells relative to total cell number determined by trypan blue exclusion assay after 6 d of treatment of BT40. Right: The percent of cells relative to the solvent control. Data shown represents the mean ± SD of three independent biological replicates. All concentrations indicated on the x-axis are expressed in nM. **C** Percentage of fragmented nuclei relative to all nuclei. BT40 cells were treated for 72 h with single agents or in combination with binimetinib or trametinib at different concentrations (IC_25_, IC_50_, 2x IC_50_, IC_75_) before being analyzed. Data represents the mean ± SD of three independent biological replicates with small dots indicating individual wells. All concentrations indicated on the x-axis are expressed in nM. IC, inhibitory concentration. **D** Percentage of Caspase-positive cells relative to total cell number. BT40 cells were treated for 72 h with single agents or in combination with binimetinib or trametinib at different concentrations (IC_25_, IC_50_, 2x IC_50_, IC_75_) before being analyzed. Data represents the mean ± SD of three independent biological replicates with small dots indicating individual wells. All concentrations indicated on the x-axis are expressed in nM. **E** Caspase-3/7 activity measured using a fluorescent DEVD-based substrate in cell lysates of DKFZ-BT314_OFF after 48-72h of treatment (cells were collected once detachment was observed). Values are normalized to the solvent control. Bars represent the mean ± SD of three biological replicates. D, DMSO; b, binimetinib; t, trametinib; d, dabrafenib; n, navitoclax; s, selinexor; v, venetoclax.

**Suppl. Figure 6. Combination treatment with MEKi and either navitoclax or selinexor induces cell death in BT40 cells. A** Left: Percentage of dead cells relative to total cell number determined by trypan blue exclusion assay after 6 d of treatment of BT40 cells, seeded as monolayers. Right: The percent of cells relative to the solvent control. Data shown represents the mean ± SD of three independent biological replicates. All concentrations indicated on the x-axis are expressed in nM. **B** Percentage of fragmented nuclei relative to all nuclei following 72h of treatment as indicated. Data represents the mean ± SD of three independent biological replicates with small dots indicating individual wells. All concentrations indicated on the x-axis are expressed in nM.

**Suppl. Figure 7. Effect of selinexor and MEK inhibition in a zebrafish embryo xenograft model. A** Representative fluorescence images of zebrafish embryos bearing BT40 xenografts following treatment with DMSO, selinexor, trametinib, or the corresponding combination treatment. Tumor burden is visualized by red fluorescence. Images were acquired at day0 and day3. Scale bars, 100 µm. **B** Quantification of tumor response expressed as change from baseline. Each dot represents one individual zebrafish embryo. Bars indicate the mean, and error bars represent standard deviation (SD). Negative values indicate tumor regression relative to baseline, whereas positive values indicate tumor growth. Statistical significance was determined using ANOVA test.

**Suppl. Figure 8. Ex vivo DSP of pLGG patient-derived tumoroid cultures. A** Heatmap depicting % inhibition at highest concentration applied (‘pMAX’) in comparison with other tested compounds across 20 pediatric low-grade glioma samples collected within the LOGGIC Core BioClinical Data Bank. Patient-derived *ex vivo* fresh tissue tumoroid cultures (FTCs) were directly established and exposed for 72 h to the indicated drugs. Drug sensitivity was assessed by measuring metabolic activity using CellTiter-Glo 2.0. **B** Scatter plot showing the correlation between selinexor and navitoclax responses across samples for pMAX. Each dot represents one sample. The blue line indicates the linear regression fit, and the shaded grey area represents the 95% confidence interval of the regression line estimated by bootstrap resampling. Pearson correlation coefficients (r) and corresponding p-values are shown. **C** Pearson correlation matrices showing pairwise correlations between drug response profiles across all compounds included in the screening heatmaps for pTOX. Correlations were calculated using raw, non-normalized drug response values. Each cell represents the Pearson correlation coefficient (*r*) between two compounds across all samples with complete data. Compounds are displayed in a fixed order corresponding to their drug classes. Color intensity indicates the strength and direction of correlation, with positive correlations shown in yellow/green and negative correlations shown in blue/purple. Abbreviations: DSP, Drug sensitivity profiling; pLGG, pediatric low-grade glioma; INFORM, INdividualized therapy FOr high-Risk childhood Malignancies; FTC, fresh tissue tumoroid cultures**; LOGGIC, Low-Grade Glioma in Children. D-E** Heatmaps of SASP genes (**D**) and MAPK Pathway Activity Score (MPAS) genes (**E**) showing unsupervised clustering based on log₂-normalized expression values.
